# Ligand-Directed Site-Selective Cysteine Bioconjugation of the KELCH Domain of KEAP1 with Hypervalent Iodine Reagents

**DOI:** 10.1021/jacs.5c13391

**Published:** 2025-11-04

**Authors:** Christine Marty, Xinjian Ji, Stefano Nicolai, Christian Heinis, Jerome Waser

**Affiliations:** † Laboratory of Catalysis and Organic Synthesis, Institute of Chemical Sciences and Engineering, Ecole Polytechnique Fédérale de Lausanne, Ch-1015 Lausanne, Switzerland; ‡ Laboratory of Therapeutic Proteins and Peptides, 27218 Institute of Chemical Sciences and Engineering, Ecole Polytechnique Fédérale de Lausanne, CH-1015 Lausanne, Switzerland

## Abstract

Affinity-driven reactions have allowed chemists to perform site-selective modifications of native proteins. By combining the high cysteine chemoselectivity of hypervalent iodine-based ethynylbenziodoxolones (EBXs) with the site selectivity of peptide ligands known to inhibit protein–protein interactions, we achieved site-selective labeling of Cys434 in the KELCH domain of Kelch-like epichlorohydrin-associated protein 1 (KEAP1), a key protein in the regulation of oxidative stress. EBXs could be used either as traceless reagents with release of the peptide ligand to introduce reactive handles such as azides or alkynes or as covalent reagents leading to the formation of peptide–protein adducts, which could be cleaved in a separated step.

## Graphical abstract

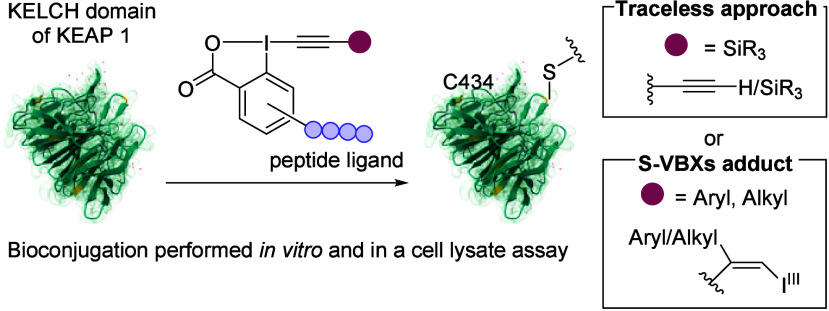

## Introduction

Site-selective chemistry for labeling proteins has been extensively studied over the last decades to develop better diagnostic and therapeutic tools.^1^ Many site-selective labeling techniques require genetical modification of the protein of interest (POI) to incorporate protein tags,^2^ peptide tags,^3^ or unnatural amino acids enabling biorthogonal reactions.^4^ Recently, affinity-driven reactions have allowed the site-selective labeling of native, nontagged proteins with chemical probes.^5–9^ In this approach, a ligand, often derived from the natural binding ligand, and carrying a reactive group, binds reversibly to the target through noncovalent interactions (Scheme 1A).^5,6^ Upon binding, the reactive group is brought in close proximity to a reactive side chain of the protein, resulting in a covalent bond. Alternatively, a catalyst is introduced onto the target, which then promotes a reaction between the protein and a reagent in solution.^7–9^ Hamachi and co-workers developed traceless methods termed ligand-directed chemistry (LD), in which the ligand is released during the labeling reaction, which prevents the loss of the native activity of the protein.^6^ Currently, most methods for LD are used with small molecules as ligands. However, developing ligands based on small molecules may be challenging for some proteins or specific surface regions of proteins.^10^

**Scheme 1:**
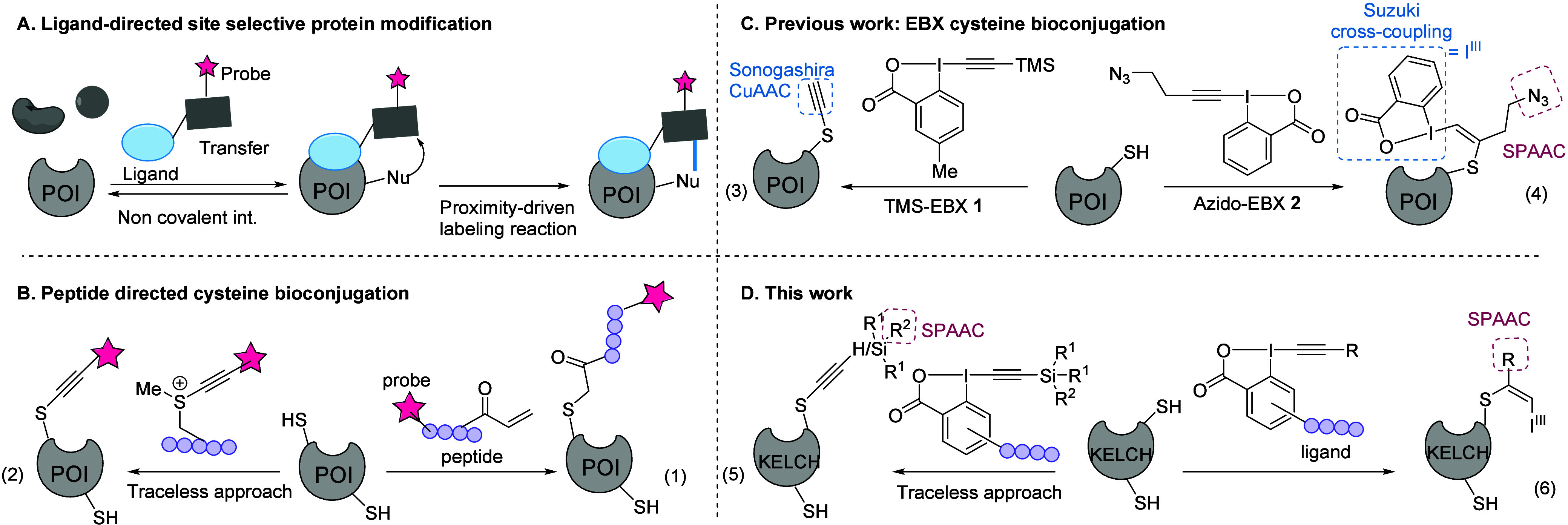
Schematic Representation of Previous Methods and the Site-Selective Cysteine Bioconjugation with EBX Reagents Used in This Work^a^

a (A) Ligand-directed site-selective protein modification; (B) peptide-directed cysteine bioconjugation; (C) modification of cysteine with EBX reagents; and (D) this work, site-selective cysteine bioconjugation with EBX reagents.

In this context, peptides and peptidomimetics have emerged as promising ligands for these challenging targets due to their larger size. Furthermore, it has been estimated that 15–40% of all PPIs are mediated by a short linear peptide,^11^ which can immediately serve as a design starting point for ligands. Examples of peptide affinity-guided conjugation using catalysis have been reported, employing a metal dirhodium metallopeptide catalyst,^7^ an Ir photocatalyst,^8^ or DMAP-based reagents.^9^

Cysteine has been dominating residue-selective bioconjugation methods due to the high nucleophilicity of thiols.^12^ However, this makes site-selective functionalization when several cysteines are present especially challenging. Only few peptide-based affinity-driven bioconjugation methods have been reported, in contrast to the well-established use of peptides bearing an electrophilic warhead for cysteine-based covalent inhibition.^13^ Xia and co-workers designed fluorescent probes containing an acrylamide to target bromodomains (BRDs) binding through cysteine functionalization (Scheme 1B, eq 1).^14a^ However, after covalent modification of the POI, the ligand remains attached to the protein.^14^ A traceless approach was developed by Li’s group using sulfonium probes to selectively label cysteine 34 on the DPZ protein containing three cysteine residues (Scheme 1B, eq 2).^15^ Therefore, methods enabling site-selective modification of more complex proteins of high biological relevance bearing multiple free cysteines would be of high interest. In this context, the KELCH domain of Kelch-like epichlorohydrin-associated protein 1 (KEAP1) is a particularly challenging target, as it contains nine free cysteines, which react with external electrophiles. For example, sulforaphane modified cysteines C489, C513, and C518, while the natural product pubescenoside A only modified C434.^16^ Modifying cysteines on KEAP1, including C434, had a strong effect on the regulation of NRF2 ubiquitination.^16d^ Furthermore, the KEAP1-NRF2 (nuclear factor erythroid 2-associated factor 2) pathway is involved in the regulation of oxidative stress, which is associated with various diseases.^17^ Based on the KEAP1-NRF2 PPI interaction, the 4-amino acid motif ETGE in Nrf2 was found to bind KEAP1 on the KELCH domain.^18^ A range of peptides containing this motif and binding with nM affinity were reported, all binding close to Cys434.^19^ They could therefore serve as a basis for a ligand-directed approach to specifically modify Cys434. Furthermore, inhibitors reacting covalently with cysteines residues were inducing conformation changes, leading to dissociation of NRF2.^19c^

Our group reported the use of hypervalent iodine-based ethynylbenziodoxolone (EBX) reagents for cysteine-selective bioconjugation on proteins. Using TMS-EBX derivative **1**, we achieved the selective ethynylation of cysteine residues under physiological conditions (Scheme 1C, eq 3).^20^ In contrast, when using AlkylN_3_-EBX **2**, vinylbenziodoxolone (VBX) adducts were formed, providing a double orthogonal handle for further protein modifications (eq 4).^21^ However, in both cases, all accessible cysteines were modified. We therefore wondered if EBX reagents bearing peptide ligands could be used for affinity-driven bioconjugation on the KELCH domain of KEAP1. Herein, we report the successful implementation of this strategy (Scheme 1D). On one hand, alkynylated Cys434 was obtained through a traceless approach via ligand release using silylated EBXs (eq 5). On the other hand, S-VBXs were formed without cleavage of the hypervalent bond and the ligand using Alkyl- and Aryl-EBXs (eq 6). The KELCH-peptide hypervalent iodine conjugated could be cleaved reductively after S-VBX modification. Finally, labeling of the KELCH domain of KEAP1 was achieved via the strain-promoted azide–alkyne click chemistry reaction (SPAAC) on both S-VBX and the alkynylated product.

## Results and Discussion

### Synthesis and Reactivity on Peptides of the Peptide-EBX Reagents

Our investigation began with the synthesis of a model peptide-EBX in order to assess the stability and reactivity of the reagent in aqueous buffers. Previously, our group developed a method for the late-stage introduction of EBXs onto peptides by a selective amidation between an activated ester located on the alkyne of the EBXs and a free amine at the N-terminus or a lysine on the peptide.^22^ However, the resulting reagents **3** would not be compatible with a traceless modification as the alkynylation product would also contain the peptide (Scheme 2A). Therefore, we designed new reagents **4** bearing the peptide on the aryl core of the EBX (Scheme 2A).

**Scheme 2:**
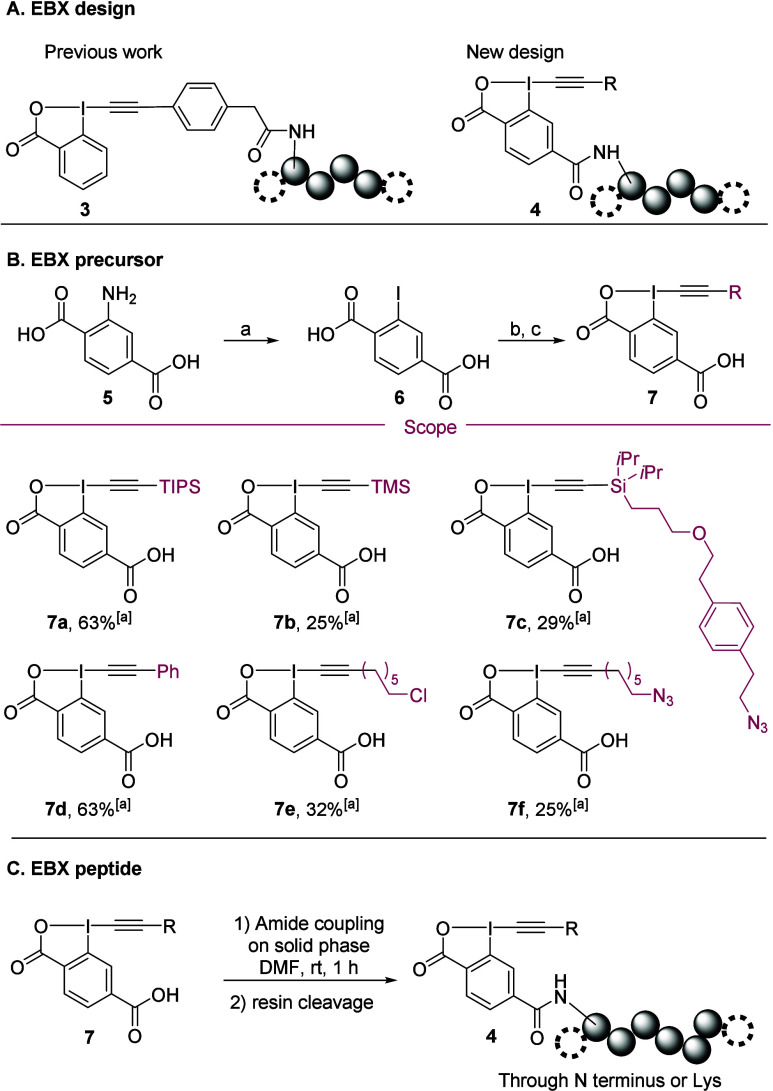
(A) EBX Design for Ligand-Directed Conjugation, (B) EBX Synthesis, and (C) EBX Peptide Formation

a Yield given for step C.
b Reaction conditions: (A) NaNO_2_, HCl, KI, 77%; (B) NaIO_4_, 72%; (C) LA, TMS-alkynyl precursor, pyridine.

The carboxylic acid EBX precursors **6** for peptide coupling were synthesized in three steps from commercially available aniline **5** via a Sandmeyer reaction, followed by oxidation of the resulting iodide **6** and reaction with TMS-alkynes (Scheme 2B). Although this procedure proceeds in moderate yield, it is general and gives rapid access to all types of alkynes: silylated (**7a**–**c**), arylated (**7d**), and alkylated (**7e**–**f**) on preparative scale (95–900 mg). Then, the EBX precursor was coupled directly on solid phase though the N-terminus or a lysine residue and released from the resin, giving access to a broad range of peptide derivatives **4** (Scheme 2C, the efficiency of the coupling varies depending on the peptide sequence, see Supporting Information for details). As a proof of concept, we first investigated the reactivity of TIPS-EBX reagent **4aa** with a 6-mer peptide. After initial screening of several buffers (Tris, PBS, HEPES at pH = 8.6), good conversion values were obtained in all cases (see the SI).^23^

### Labeling of the KELCH Domain of KEAP1

Then, we investigated the influence of four ligands on the bioconjugation of the KELCH domain of the protein KEAP1 (residues 321–609), which can be efficiently recombinantly expressed in contrast to the full protein (**8a**, Scheme 3).

**Scheme 3:**
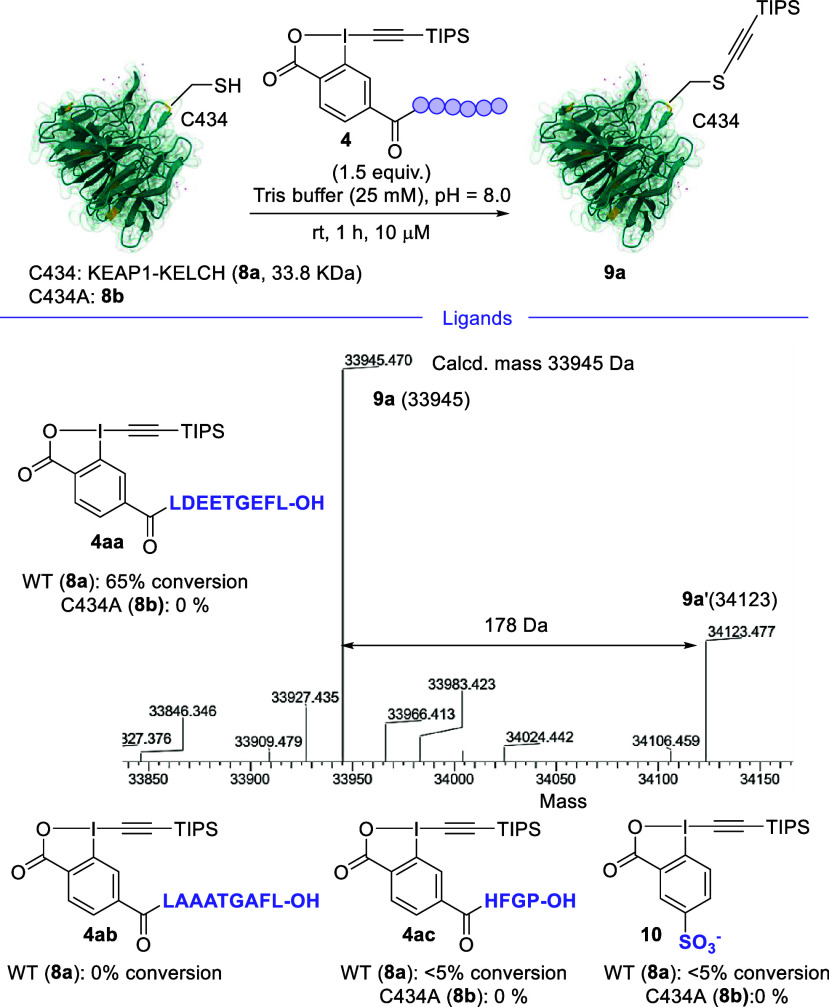
Influence of the Protein Ligand on the Labeling of the KELCH Domain of KEAP1

a Reaction conditions: KEAP1-KELCH (**8a**, 10 μM), EBX (**4**, 1.5 equiv) in Tris buffer (25 mM, pH 8.0), 21 °C, 1 h (PDB 5WFV). Peaks corresponding to the gluconoyl adduct are indicated with an apostrophe. Conversions were calculated by dividing the MS peak intensity of the product by the sum of the intensities of all peaks, including impurities already present in **8a**.

The recombinantly expressed and purified KELCH domain (**8a**) occurred as a mixture with a post-translationally modified protein (N-terminal d-gluconoyl derivative; **8a**′, +178 Da; around 55%).^24^ We incubated KEAP1-KELCH (**8a** and **8a**′) with the EBX peptide **4** (1.5 equiv, 15 μM) in Tris buffer (25 mM, pH 8.0) at RT for 1 h. When the 9-mer peptide LDEETGEFL ligand^19^ was attached to the EBX (**4aa**), 65% conversion to the alkynylation products **9a** and **9a**′ was achieved, with no starting **8a** remaining. When the same reaction was performed on the mutant KEAP1-KELCH C434A (**8b**), no conversion occurred, confirming the modification was selective for C434. Additionally, the top-down MS/MS experiment confirmed that the modification occurred on C434 (see SI). When all negatively charged amino acids were mutated to alanine (EBX **4ab**) bearing the peptide LAAATGAFL, no conversion was observed.

When the WT peptide was replaced with a random tetramer sequence (EBX **4ac**), the conversion was poor. The negatively charged water-soluble TIPS EBX **10** gave low conversion on KEAP1 WT and no conversion on KEAP1 C434A. We speculated that this reagent may still bind to the positively charged pocket around C434, preventing reaction on other cysteines. As additional control, neutral azide EBX **2** was examined, and in this case, formation of S-VBX adducts was indeed observed on both KEAP WT and KEAP C434A (see the SI). Therefore, the first LD selective Cys modification using an EBX reagent had been achieved.

We then examined other EBX reagents. TMS-EBX **4b** gave 77% conversion to free alkyne product **9b** under the same reaction conditions (Scheme 4A). However, the larger silyl-EBX **4ca** bearing an azide tag gave the alkynylated product **9c** in only 10% conversion, due mostly to cleavage of the Si–C bond to give the simple alkynylation product **9b** again. We wondered if attaching this larger reagent on a Lys side-chain rather than the N-terminus would lead to more efficient bioconjugation. We decided to install the Lys on the C-terminus of the peptide (Ac-LEDDTGEFK-NH_2_) as it is closest to C434 according to the X-ray structure (Scheme 4B, PDB 5WFV). Gratifyingly, EBX **4cb** gave alkynylated product **9c** in 57% conversion. In the case of Ph-EBX **4d**, Chloro-EBX **4e**, and Azido-EBX **4f**, the use of the standard N-terminus bound reagents resulted in 88%, 61%, and 77% conversion, respectively, to the expected S-VBXs products **11a**–**c** (Scheme 4C). Only in the cases of **11b** and **11c**, small amounts of alkynylated cysteine impurities were observed.

**Scheme 4:**
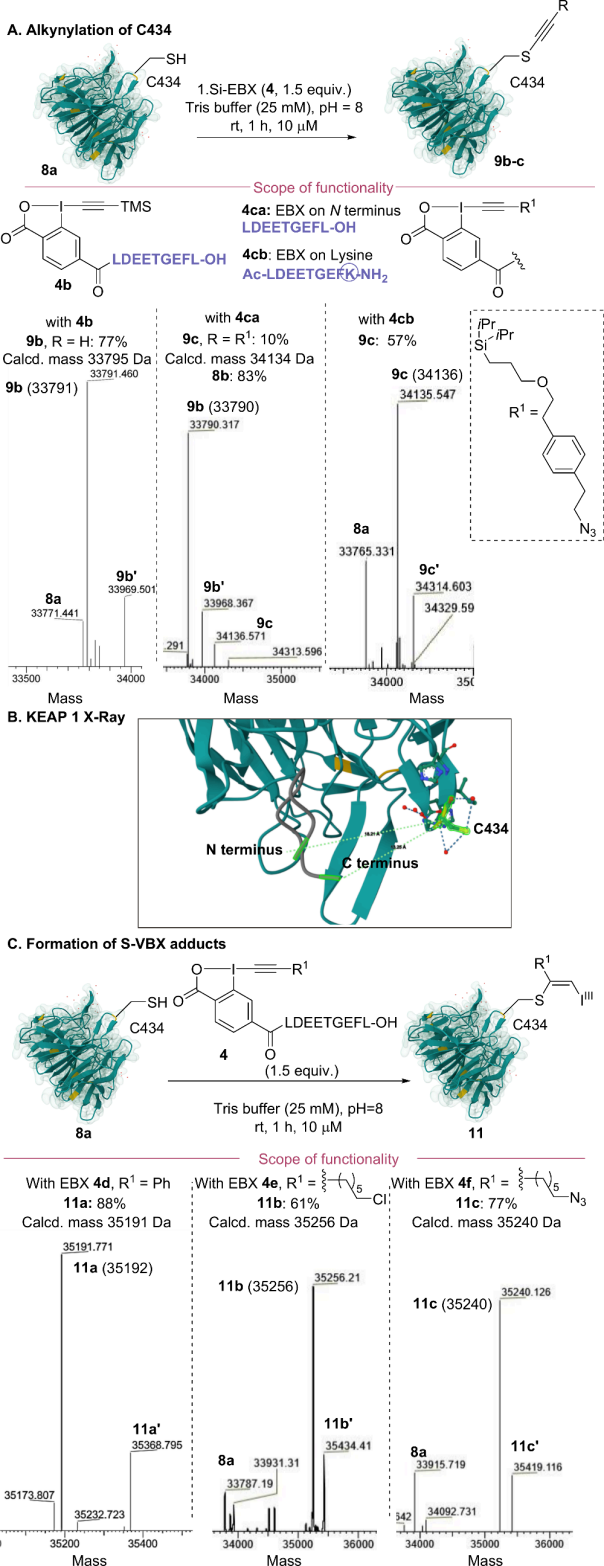
(A) Alkynylation of C434, (B) X-ray of KEAP1 and the 9-mer Ligand, and (C) Formation of S-VBX Reagents

a Reaction conditions: KEAP1-KELCH (**8a**, 10 μM), EBX (**4**, 1.5 equiv) in Tris buffer (25 mM, pH 8.0), 21 °C, 1 h (PDB 5WFV). Peaks corresponding to the gluconoyl adduct are indicated with an apostrophe. Conversions were calculated as in Scheme 3.

### Post Modification of Bioconjugates

We then attempted to further functionalize the bioconjugate via copper-free SPAAC in a one-pot two-step procedure. After incubating Azido-EBX **4f** with KEAP1-KELCH (**8a**) for 1 h, a bicyclo[6.1.0]­non-4-yne functionalized with a fluorescein fluorophore (BCN-dye **14**) was added. The labeled product **12** was obtained in 64% yield (Scheme 5A, eq 1). The reaction was very clean, as the only other observed product was intermediate S-VBX **11c**. The SPAAC was also successful using the silyl-N_3_ reagent **4cb** switching Tris buffer for PBS buffer allowing the formation of **13** (eq 2). Based on previous reports from our group, we also attempted a Suzuki–Miyaura cross-coupling between the hypervalent iodine bond of S-VBX and boronic acids.^15^ Unfortunately, cross coupling was never observed. However, we observed one product with a mass signal corresponding to the reduction of the C–I^III^ bond. After investigating several hydride sources as reductant (see the SI), the ligand could be removed to give **15** in a one-pot two-step procedure with 23% yield in tris buffer using Pd (10 equiv) and NaCOOH (20 equiv) (Scheme 5B, eq 3). Interestingly, when the buffer was changed to PB buffer and the amount of hydride source increased to 30 equiv, the C–S bond was cleaved, leading back to the WT KEAP1-KELCH (**8a**) in 69% conversion (eq 4). We also attempted to modify the free alkyne handle on the protein using copper-catalyzed azide–alkyne cycloaddition (CuAAC). However, this method was incompatible with KEAP1-KELCH, probably due to the presence of multiple free cysteines.

**Scheme 5:**
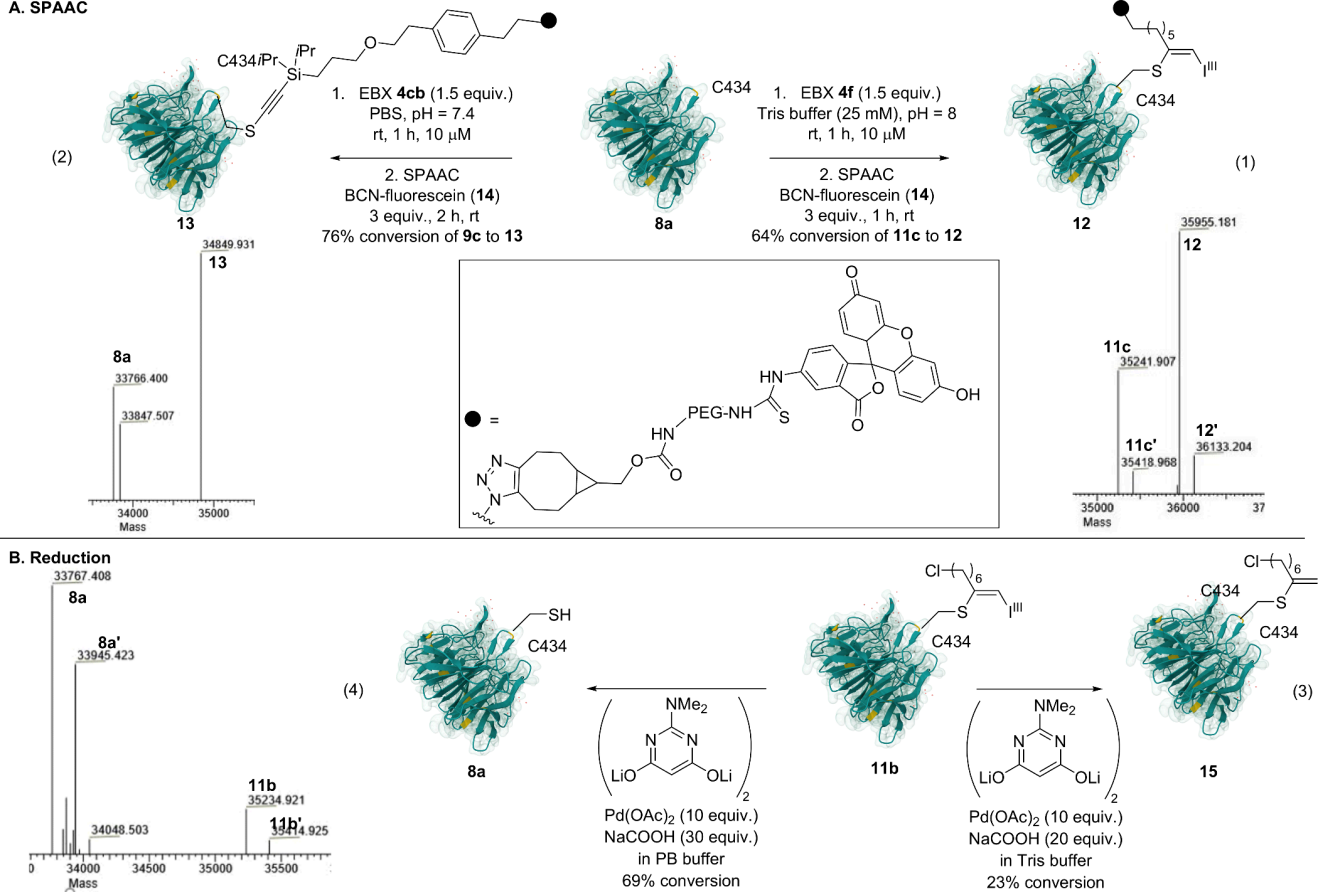
Post Modifications: (A) SPAAC Reaction and (B) Reduction

a Reaction conditions: (A) (1) KEAP1-KELCH (**8a**, 10 μM), EBX (**4**, 1.5 equiv) in Tris buffer (25 mM, pH = 8.0), 21 °C, 1 h, then BCN-dye **14** (3 equiv) for 1 h. (2) Similar conditions as (1) but in PBS (pH = 7.4). (B) Palladium complex **Pd-1** (10 equiv), NaCOOH (20 or 30 equiv) in Tris buffer (25 mM, pH = 8.0) or PB buffer (25 mM, pH = 8.0), 21 °C, 1 h. The reaction was quenched with 3-mercaptopropionic acid in water (5.00 equiv. per equiv of palladium), 10 min. Peaks corresponding to the gluconoyl adduct are indicated with an apostrophe.

### Labeling of the KELCH Domain of KEAP1 in Mixtures of Proteins

Finally, we investigated the site selectivity of the bioconjugation strategy in a more complex environment. A 1:1 mixture of BSA (**16**), containing one free cysteine, and KEAP1-KELCH (**8a**) was treated with 5 equiv of EBX **4aa**. Under these conditions, we observed 84% conversion of KEAP1-KELCH (**8a**) to thio alkyne **9a**, while BSA (**16**) remained unmodified (Scheme 6A). This result demonstrated that site-selective functionalization remains possible in the presence of one other Cys-containing protein at the same concentration. To evaluate the potential of the method in more complex settings, the recombinantly expressed KELCH domain of KEAP1 (**8a**, 0.3 mg/mL) was added to a U2OS cell lysate (0.9 mg/mL) and then treated with EBX **4cb** and the BCN-functionalized dye **14** (see the SI). SDS-PAGE analysis of the reaction mixture revealed labeling of KEAP1-KELCH (**8a**) even in the presence of an excess of multiple other proteins from the lysate, as well as other thiol-containing biomolecules such as glutathione (Scheme 6B, lane 1). The signal intensity was comparable to the one obtained in absence of cell lysate (lane 2). Without **8a**, no strong signal could be observed (lane 3). However, a weaker but significantly labeled bioconjugate coming just below in the gel was observed when **8a** was treated directly with the BCN-dye **14** in the absence of EBX **4cb** in the cell lysate (lane 4). It has been reported that a background thiol–yne reaction can take place between cyclooctynes and free cysteines,^25^ which could explain this result when considering the high reactivity of the free cysteines on KEAP1-KELCH (**8a**). Indeed, direct labeling of **8a** was observed by fluorescence when it was treated with **14** in the absence of cell lysate (lane 5). However, when lanes 2 and 5 were analyzed by MS, no signal corresponding to the thiol–yne adduct was observed, although such adducts are known to be stable in MS^25b^ (see Scheme 5A and the SI). This seems to indicate that SPAAC is more efficient than the thiol–yne reaction, which is a minor side reaction observable only with the higher sensitivity of fluorescence spectroscopy compared to MS.

**Scheme 6:**
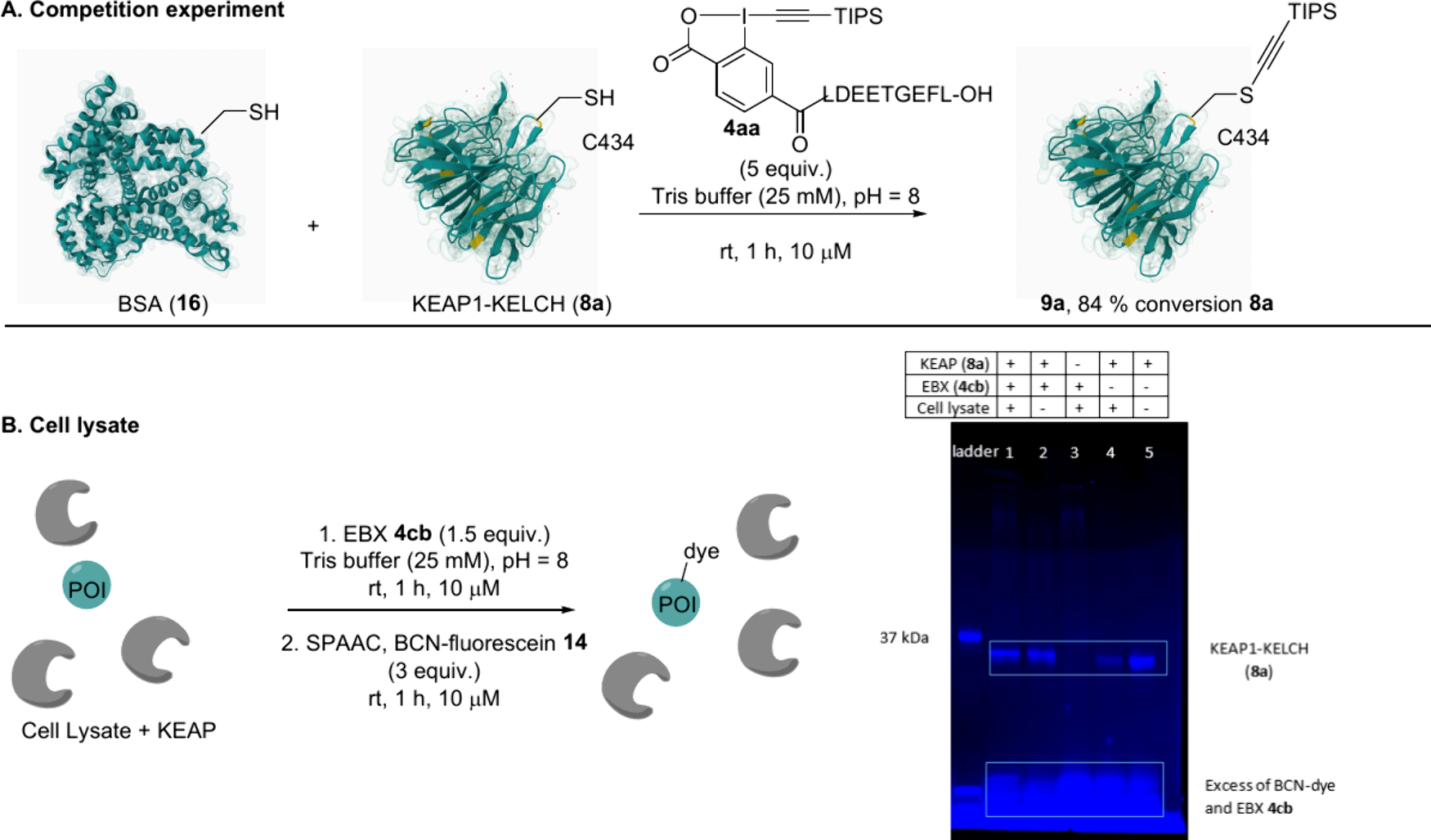
(A) Competition Experiment with BSA and (B) Cell Lysate and Fluorescence Image of SDS-PAGE

a Reaction conditions: KEAP1-KELCH (**8a**, 10 μM), BSA (**16**, 10 μM), EBX **4aa** (5 equiv) in Tris buffer (25 mM, pH = 8.0), 21 °C, 1 h.
b KEAP1-KELCH (**8a**, 10 μM), EBX **4cb** (1.5 equiv), then BCN-dye **14** (3 equiv) in 25 mM Tris buffer, pH = 8, 21 °C, 0.9 mg/mL U2OS cell lysate.

## Conclusion

In summary, we have developed an EBX-peptide conjugate capable of site-selective cysteine bioconjugation of the KELCH domain of KEAP1 under mild conditions. We successfully introduced an azide handle that enabled subsequent SPAAC reactions. Functionalization was possible both with and without release of the directing peptide. Release of the peptide ligand was possible either during the reaction or with less efficiency in a second Pd-mediated step. The selective labeling of the KELCH domain of KEAP1 in the presence of BSA and in the complex environment of a cell lysate confirmed the robustness and specificity of this bioconjugation strategy.

## Supplementary Material



## Data Availability

Raw data for NMR, MS, IR, and HPLC available free of charge from zenodo.org at 10.5281/zenodo.17264289.

## Notes

General methods, synthetic procedures, HPLC methods, HRMS spectra, characterization data for reported and new compounds, and copy of ^1^H and ^13^C NMR spectra for new compounds (PDF)

## Abbreviations

EBX: ethynylbenziodoxolones
PPIs: protein–protein interactions
KEAP1: Kelch-like epichlorohydrin-associated protein 1
POI: protein of interest
LD: ligand-directed
SPAAC: strain-promoted azide–alkyne click chemistry reaction
BCN: bicyclo­[6.1.0]­non-4-yne
