# Supplementary material for: Ligand-Directed Site-Selective Cysteine Bioconjugation of the KELCH Domain of KEAP1 with Hypervalent Iodine Reagents
Source: J Am Chem Soc. 2025 Nov 4;147(46):42524–31. doi: 10.1021/jacs.5c13391 (PMC12643532; doi:10.1021/jacs.5c13391)

Supplementary Information for:

Ligand-Directed Site Selective Cysteine Bioconjugation of the KELCH domain of KEAP1 with Hypervalent Iodine Reagents

Christine Marty[a], Xinjian Ji[b], Stefano Nicolai[a], Christian Heinis[b], and Jerome Waser*[a]

[a]Laboratory of Catalysis and Organic Synthesis, Institut des Sciences et Ingénierie Chimique, Ecole Polytechnique Fédérale de Lausanne, Ch-1015, Lausanne, Switzerland.

[b] Laboratory of Therapeutic Proteins and Peptides, Institute of Chemical Sciences and Engineering, Ecole Polytechnique Fédérale de Lausanne, CH-1015 Lausanne, Switzerland

*E-mail: Jerome.waser@epfl.ch

Contents

1. General methods ... 2
2. HPLC-MS and preparative HPLC information ... 3
3. Synthesis of hypervalent iodine reagents ... 4
   a. Synthesis of alkyne substrates ... 4
   b. Synthesis of EBX ... 9
   c. Synthesis of EBX peptide ... 14
5. Protein expression ... 27
6. Protein bioconjugation ... 33
   a. Influence of the ligand ... 34
   b. Scope of EBX ... 43
7. Protein Post Modification ... 49
8. Cell lysate ... 59
9. Top-down experiment mass spectrometry analysis ... 63
10. NMR spectrum ... 64

1. General methods

All reactions were carried out in oven dried glassware under an atmosphere of nitrogen, unless stated otherwise. For quantitative flash chromatography, technical grade solvents were used. For flash chromatography analysis, HPLC grade solvents from Sigma-Aldrich were used. THF, Et_2O, CH_3CN, toluene, hexane and CH_2Cl_2 were dried by passage over activated alumina under nitrogen atmosphere (H_2O content < 10 ppm, Karl-Fischer titration). All chemicals were purchased from Acros, Aldrich, Fluka, VWR, Aplichem, or Merck and used as such unless otherwise stated. All the Fmoc-protected amino acids and Trt-Cl resin were purchased from GL Biochem or Bachem. 1- [Bis(dimethylamino)methylene]-1H-1,2,3-triazolo[4,5-b]pyridinium 3-oxide hexafluorophosphate (HATU, Bachem) and N,N-diisopropylethylamine (DIPEA, Iris Biotech GmbH) were used as received. Chromatographic purification was performed as flash chromatography using Macherey-Nagel silica 40-63, 60 Å, with the solvents indicated as eluent under 0.1-0.5 bar pressure. TLC was performed on Merck silica gel 60 F254 TLC glass plates or aluminium plates and visualized with UV light, permanganate stain, CAN stain, or Anisaldehyde stain. ^{1}H-NMR spectra were recorded on a Brucker DPX-400 400 MHz spectrometer in $CDCl_3$, DMSO-d_6 CD_3OD, C_6D_6 and CD_2Cl_2, all signals are reported in ppm with the internal chloroform signal at 7.26 ppm, the internal DMSO signal at 2.50 ppm the internal methanol signal at 3.30 ppm, the internal dichloromethane signal at 5.30 ppm as standard. The data is being reported as (s = singlet, d = doublet, t = triplet, q = quartet, quint = quintet, m = multiplet or unresolved, br = broad signal, app = apparent, coupling constant(s) in Hz, integration, interpretation).^{13}C-NMR spectra were recorded with 1H-decoupling on a Brucker DPX-400 100 MHz spectrometer in $CDCl_3$, DMSO-d_6, CD_3OD or CD_2Cl_2, all signals are reported in ppm with the internal chloroform signal at 77.0 ppm, the internal DMSO signal at 39.5 ppm, the internal methanol signal at 49.0 ppm and the internal dichloromethane signal at 54.0 ppm as standard. Structural assignments were made with additional information from gCOSY, gHSQC, and gHMBC experiments. Infrared spectra were recorded on a JASCO FT-IR B4100 spectrophotometer with an ATR PRO410-S and a ZnSe prisma and are reported as cm-1 (w = weak, m = medium, s = strong, br = broad). High resolution mass spectrometric measurements were performed by the mass spectrometry service of ISIC at the EPFL on a MICROMASS (ESI) Q-TOF µLtima API, LTQ Orbitrap ELITE ETD (Thermo fisher), Xevo G2-S QTOF (Waters), or LTQ Orbitrap ELITE ETD (Thermo fisher).

2. HPLC-MS and preparative HPLC information

HPLC-MS analysis

HPLC-MS measurements were performed on an Agilent 1290 Infinity HPLC system with a G4226a 1290 Autosampler, a G4220A 1290 Bin Pump and a G4212A 1290 DAD detector, connected to a 6130 Quadrupole LC/MS, coupled with a Waters XBridge C18 column (250 x 4.6 mm, 5 µm). Water:acetonitrile 95:5 (solvent A) and water:acetonitrile 5:95 (solvent B), each containing 0.1% formic acid, were used as the mobile phase, at a flow rate of 0.6 mL.min^{-1}. The gradient was programmed as follows:

Method 1. 100% A to 100% B in 20 minutes then isocratic for 5 minutes.

The column temperature was set up to 25 °C. Low-resolution mass spectrometric measurements were acquired using the following parameters: positive electrospray ionization (ESI), temperature of drying gas = 350 °C, flow rate of drying gas = 12 L. min^{-1}, pressure of nebulizer gas = 60 psi, capillary voltage = 2500 V and fragmentor voltage = 70 V.

Preparative HPLC

Preparative RP-HPLC were performed on an Agilent 1260 HPLC system with a G2260A 1260 Prep ALS Autosampler, a G1361a 1260 Prep Pump, a G1365C 1260 MWD detector and a G1364B 1260 FC-PS collector, coupled with a Waters XBridge semi-preparative C18 column (19 x 150 mm, 5 µm). Water (solvent A) and water:acetonitrile 5:95 (solvent B), each containing 0.1% formic acid, were used as the mobile phase at a flow rate of 20 mL.min^{-1}.

Method 2: 100% A to 100% B in 25 minutes.

Solid-Phase Peptide Synthesis (SPPS):

Peptides were synthesized on an MultiPep RSi parallel peptide synthesizer (Intavis) using standard Fmoc SPPS-chemistry and 2-chlorotrityl chloride resin (1.38 mmol/g, 100-200 mesh). The first amino acid was loaded on the resin by incubation of the Fmoc-protected monomer (3 equiv of the number of active sites on the resin), DIPEA (4 equiv) in dichloromethane for 2 h. Each coupling cycle was initiated by Fmoc deprotection achieved by shaking the resin with 800 µL of 20% v/v 4 piperidine in dimethylformamide (DMF), over 10 minutes twice. Then the resin was washed with DMF (1500 µL x4). The coupling was carried out by shaking 2-chlorotrityl chloride resin with a Fmoc-protected monomer (5.0 equiv), HATU (5.0 equiv), N-Methylmorpholine (10 equiv), in DMF (1.3 mL), over 60 minutes. The synthesis was finished by deprotection of Fmoc using 20% v/v piperidine in dimethylformamide at 400 rpm, over 10 minutes two times. The N-terminus was either left unprotected. Next, washing steps were performed with dimethylformamide (5 x 1 mL). Finally, resin was dried with dichloromethane (5 x 3 mL).

Peptide cleavage and deprotection:

Peptides without protecting groups

Peptides were deprotected and cleaved from the resin by treatment with a 20% solution of HFIP in DCM. The resulting mixture was shaken for 1 hour at room temperature. The resin was removed by filtration and peptides were precipitated in cold diethyl ether (50 mL), followed by a 2 hour incubation

at -20 °C. Peptides were pelleted by centrifugation at 4000 rpm, for 5 minutes. Finally, the mother liquors were carefully removed.

3. Synthesis of hypervalent iodine reagents

a. Synthesis of alkyne substrates

(3-(4-(2-Azidoethyl)phenethoxy)propyl)diisopropyl((trimethylsilyl)ethynyl)silane (S1)

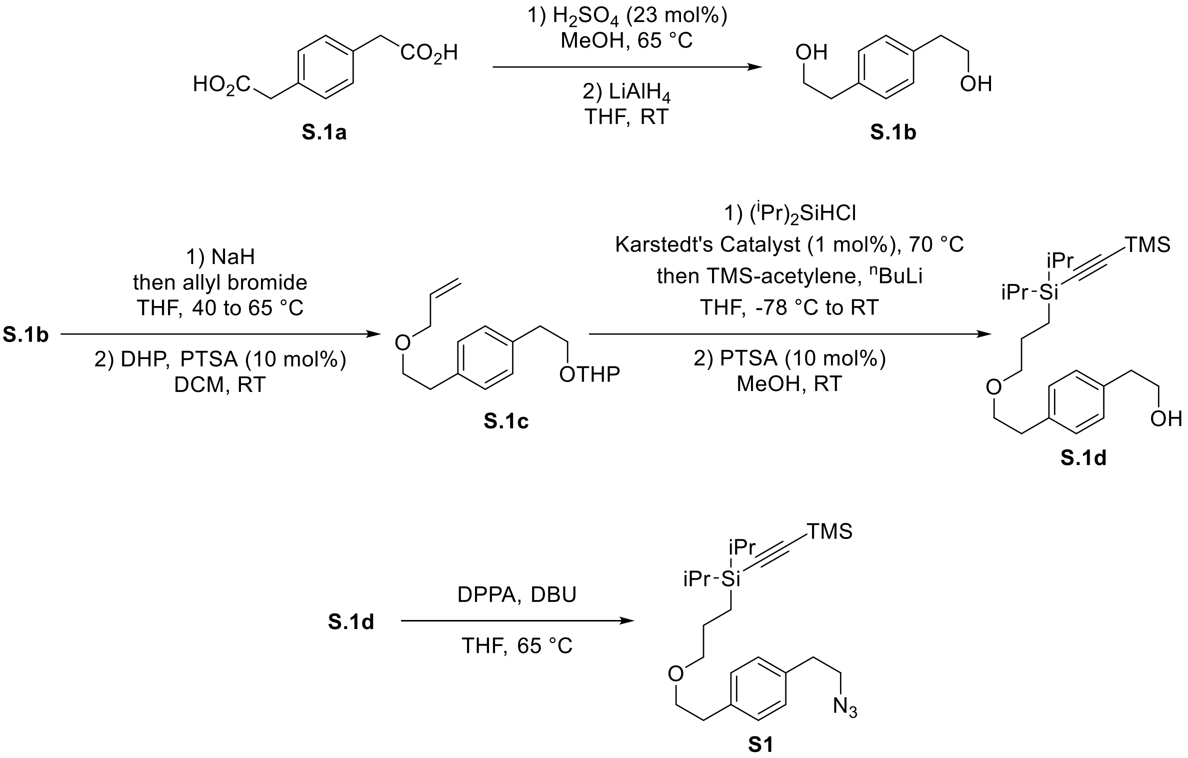

Following a reported procedure, in a 25 mL round-bottomed vial, 1,4-phenylenediacetic acid (**S.1a**) (1.55 g, 8.00 mmol, 1.0 equiv.) was suspended in MeOH (16 mL). Concentrated H_2SO_4 (0.10 mL, 1.9 mmol, 23 mol%) was added. The vial was sealed with a PTFE septum, and the mixture was stirred at 65 °C for a period of 60 hours. After this time, the mixture was poured into water (50 mL). The aqueous layer was then extracted with MTBE (4 x 30 mL). The combined organic layers were washed with sat. aq. $NaHCO_3$ (2 x 50 mL), brine, dried over Na_2SO_4, filtered and concentrated under vacuum to provide crude dimethyl 2,2'-(1,4-phenylene)diacetate (1.56 g, 7.03 mmol, 88% yield) as a pale yellow crystalline solid, which was found analytically pure and directly used for the following step without further purification.

^{1}H NMR (400 MHz, Chloroform-d) δ 7.24 (s, 4H, Ar*H*), 3.69 (s, 6H, OC*H*$_3$), 3.61 (s, 4H, C*H*$_2$). The experimentally acquired spectrum corresponded to the data reported in the literature.[1]

Inside a glovebox, a 100 mL two-necked, round-bottomed flask was charged with $LiAlH_4$ (2.03 g, 53.5 mmol, 2.0 equiv.). The flask was capped with septa and taken out of the glovebox. THF (dry; 53.5 mL) was added. To the resulting grey suspension, dimethyl 2,2'-(1,4-phenylene)diacetate (3.96 g, 17.8 mmol, 1.0 equiv.) was also added in portions (with release of gas). Once the addition was completed, the suspension was stirred at room temperature for 3 hours. After this time, TLC analysis (DCM/MeOH 9/1) showed the complete consumption of the starting diester and the formation of the diol. Upon

[1] Y. Tsukamoto, S. Itoh, M. Kobayashi, Y. Obora, *Org. Lett.* **2019**, *21*, 3299.

cooling the mixture to 0 °C (ice - water bath), the reaction was quenched by cautious addition of water (ca. 15 mL; vigorous release of gas), followed by aq. HCl (1.0 M; 100 mL).[2] The aqueous layer was extracted with MTBE (4 x 50 mL). The combined organic layers were washed with brine, dried over Na_2SO_4, filtered and concentrated under vacuum to provide crude 2,2'-(1,4-phenylene)bis(ethan-1-ol) (**S.1b**) (1.65 g, 9.93 mmol, 56% yield) as a pale yellow solid that was found analytically pure, and directly used for the following step as such.

^{1}H NMR (400 MHz, Chloroform-d) δ 7.19 (s, 4H, Ar*H*), 3.86 (t, J = 6.5 Hz, 4H, OC*H₂*), 2.85 (t, J = 6.6 Hz, 4H, ArC*H₂*), 1.42 (br s, 2H, O*H*). The experimentally acquired spectrum corresponded to the data reported in the literature. [3]

Under nitrogen, in a 100 mL two-necked, round-bottomed flask equipped with an air condenser, 2,2'-(1,4-phenylene)bis(ethan-1-ol) (**S.1b**) (1.12 g, 6.67 mmol, 1.0 equiv.) was dissolved in THF (dry; 43 mL). NaH (60% dispersion in mineral oil; 0.43 g, 11 mmol, 1.6 equiv.) was added in a single portion (moderate release of gas). The resulting suspension was stirred at 40 °C for 60 minutes, becoming a homogeneous, grey-orange mixture. Allyl bromide (0.59 mL, 6.7 mmol, 1.0 equiv.) was then added by syringe. The resulting mixture was refluxed overnight, converting into an off-white suspension. After 21 hours, it was allowed to cool down to room temperature. The reaction was quenched by cautious addition of water (25 mL; gas release!!), followed by sat. aq. NH_4Cl (25 mL). The aqueous layer was separated and extracted with EtOAc (3 x 50 mL). The combined organic layers were washed with brine, dried over Na_2SO_4, filtered and concentrated under vacuum in the presence of SiO_2. The crude product, dry-loaded on silica, was submitted to column chromatography (Biotage Isolera One flash chromatographer, 25 g SiO_2; MeOH in DCM, 0 to 10%) to provide 2-(4-(2-(allyloxy)ethyl)phenyl)ethan-1-ol (0.433 g, 2.10 mmol, 31% yield) as a pale-yellow oil.

^{1}H NMR (400 MHz, Chloroform-d; the signal corresponding to the unprotected OH was not resolved) δ 7.22–7.10 (m, 4H, Ar*H*), 5.91 (ddt, *J* = 17.2, 10.4, 5.6 Hz, 1H, C*H*=CH₂), 5.26 (dq, *J* = 17.2, 1.7 Hz, 1H, CH=C*H₂*), 5.17 (dq, *J* = 10.4, 1.4 Hz, 1H, CH=C*H₂*), 3.99 (dt, *J* = 5.6, 1.5 Hz, 2H, OC*H₂*), 3.85 (q, *J* = 6.3 Hz, 2H, OC*H₂*), 3.64 (t, *J* = 7.2 Hz, 2H, OC*H₂*), 2.89 (t, *J* = 7.3 Hz, 2H, ArC*H₂*), 2.84 (t, *J* = 6.6 Hz, 2H, ArC*H₂*).

In a 15 mL flat-bottomed vial, 2-(4-(2-(allyloxy)ethyl)phenyl)ethan-1-ol (0.87 g, 4.2 mmol, 1.0 equiv.) was dissolved in DCM (dry; 3.4 mL). 3,4-Dihydro-2H-pyran (DHP) (0.50 mL, 5.5 mmol, 1.3 equiv.) was then added. The colorless solution was cooled to 0 °C prior to the addition of PTSA monohydrate (0.040 g, 0.21 mmol, 5 mol%). The resulting clear solution was stirred at room temperature overnight, slowly becoming green-blue and, finally, orange-brown. After 15 hours, the solution was diluted with DCM (20 mL) and washed with sat. aq. $NaHCO_3$ (20 mL). The aqueous layer was then extracted with DCM (2 x 20 mL). The colorless combined organic layers were washed with brine, dried over Na_2SO_4, filtered and concentrated under vacuum. The resulting pale-yellow crude oil was submitted to column chromatography (Biotage Isolera One flash chromatographer, 25 g SiO_2; EtOAc in hexane, 0 to 20%) to provide 2-(4-(2-(allyloxy)ethyl)phenethoxy)tetrahydro-2H-pyran (**S.1c**) (1.05 g, 3.63 mmol, 86% yield) as a colorless oil.

^{1}H NMR (400 MHz, Chloroform-d) δ 7.20–7.09 (m, 4H, Ar*H*), 5.91 (ddt, *J* = 17.3, 10.4, 5.6 Hz, 1H, C*H*=CH₂), 5.25 (dq, *J* = 17.3, 1.7 Hz, 1H, CH=C*H₂*), 5.16 (dq, *J* = 10.4, 1.3 Hz, 1H, CH=C*H₂*), 4.59 (dd, *J* = 4.3, 2.9 Hz, 1H, O₂C*H*), 3.99 (dt, *J* = 5.6, 1.5 Hz, 2H, OC*H₂*), 3.93 (dt, *J* = 9.7, 7.3 Hz, 1H, OC*H₂*), 3.77 (ddd, *J* = 11.2, 7.9, 3.4 Hz, 1H, OC*H₂*), 3.66–3.56 (m, 3H, OC*H₂*), 3.46 (dddd, *J* = 10.9, 5.1, 3.5, 1.6 Hz, 1H,

[2] A large enough volume of aq. HCl (1.0 M) should be added in order to prevent the formation of an emulsion. The aqueous layer remained grey and turbid during the whole work-up.
[3] L. Bettoni, S. Gaillard, J.-L. Renaud, *Org. Lett.* **2019**, *21*, 8404.

OCH_2), 2.93–2.82 (td, J = 7.3, 4.3 Hz, 4H, ArCH_2), 1.81 (pd, J = 8.7, 3.5 Hz, 1H, THP-H), 1.69 (m, 1H, THP-H), 1.64–1.43 (m, 4H, THP-H).

Following a modified version of a reported procedure, under nitrogen, a 25 mL round-bottomed vial sealed with a PTFE vial was charged with 2-(4-(2-(allyloxy)ethyl)phenethoxy)tetrahydro-2H-pyran (**S.1c**) (1.05 g, 3.63 mmol, 1.0 equiv.) and Pt(0)-1,3-divinyl 1,1,1,3,3-tetramethyldisiloxane complex solution (Karstedt's catalyst) (0.1 M solution in vinyl terminated poly(dimethylsiloxane); ca. 0.36 mL, 0.036 mmol, 1.0 mol% compared to alkene S.X3) at room temperature. A turbid mixture was formed under stirring. Chlorodiisopropyl silane (1.2 mL, 7.3 mmol, 2.0 equiv.) was then added dropwise under stirring at room temperature. The mixture became yellow, remaining slightly turbid. It was then stirred at 70 °C overnight. After 17 hours, the volatiles were removed under reduced pressure (using a high vacuum pump, with heating at 50 °C). Pentane (dry; 3 mL) was added, and the resulting pale-yellow solution was filtered under nitrogen through activated charcoal layered on celite. Once the volatiles had been removed under reduced pressure, a colorless viscous oil was obtained, which was diluted in THF (dry; 2.0 mL) to provide a clear colorless solution (mixture A). In a 100 mL two-necked, round-bottomed flask TMS-acetylene (0.77 mL, 5.4 mmol, 1.5 equiv.) was dissolved in THF (dry; 18.5 mL). The solution was chilled to -78 °C (dry ice - acetone bath), prior to the addition of *n*BuLi (2.5 M in hexanes; 2.0 mL, 5.1 mmol, 1.0 equiv.). The resulting mixture was stirred at the same temperature for 10 minutes, and then at 0 °C (ice - water bath) for one hour. It was then chilled back to -78 °C, and mixture A was added by syringe. The resulting clear solution was stirred for 6 hours, while allowing it to warm to room temperature, slowly turning to pale yellow. The reaction was then quenched by addition of sat. aq. NH_4Cl (20 mL). The aqueous layer was separated and extracted with Et_2O (3 x 20 mL). The combined organic layers were washed with brine, dried over Na_2SO_4, filtered, and concentrated under vacuum. The resulting brown crude oil was submitted to column chromatography (Biotage, 25 g SiO_2; Et_2O in hexane, 0 to 25%). The so-obtained off-white oil was dissolved in MeOH (4.5 mL) and PTSA monohydrate (0.069 g, 0.36 mmol) was added. The resulting colorless solution was then stirred at room temperature for 3 hours, after which TLC analysis (EtOAc/hexanes 14/86) showed the complete conversion of the starting material and the formation of the desired alcohol. The solution was partition between Et_2O (20 mL) and aq. HCl (1.0 M; 20 mL). The aqueous layer was extracted with Et_2O (3 x 25 mL). The combined organic layers were dried over Na_2SO_4, filtered, and concentrated under vacuum. The resulting pale yellow crude oil was submitted to column chromatography (Biotage Isolera One flash chromatographer, 12 g SiO_2; EtOAc 0 to 30%) to provide2-(4-(2-(3-(diisopropyl((trimethylsilyl)ethynyl)silyl)propoxy)ethyl)phenyl)ethan-1-ol (**S.1d**) (0.328 g, 0.783 mmol, 22% yield) as a colorless oil.

^{1}H NMR (400 MHz, Chloroform-d) δ 7.19 (d, J = 8.1 Hz, 2H, ArH), 7.15 (d, J = 8.1 Hz, 2H, ArH), 3.85 (q, J = 6.4 Hz, 2H, OCH_2), 3.62 (t, J = 7.3 Hz, 2H, OCH_2), 3.43 (t, J = 6.8 Hz, 2H, OCH_2), 2.88 (m, 2H, ArCH_2), 2.84 (t, J = 5.7 Hz, 2H, ArCH_2), 1.77–1.58 (m, 2H, CH_2), 1.03 (dd, J = 9.2, 6.6 Hz, 12H, SiCH(CH_3)$_2$), 0.99–0.91 (m, 2H, SiCH(CH_3)$_2$), 0.63–0.54 (m, 2H, SiCH_2), 0.16 (s, 9H, Si(CH_3)$_3$).

Following a reported procedure, under nitrogen, inside a sealed 25 mL round-bottomed vial, 2-(4-(2-(3-(diisopropyl((trimethylsilyl)ethynyl)silyl)propoxy)ethyl)phenyl)ethan-1-ol (**S.1d**) (0.399 g, 0.954 mmol, 1.0 equiv.) was dissolved in THF (dry; 9.5 mL). The resulting solution was cooled to 0 °C (ice - water bath), prior to the addition of diphenyl phosphoryl azide (DPPA) (0.28 mL, 1.4 mmol, 1.5 equiv.). Finally, DBU (0.20 mL, 1.3 mmol, 1.4 equiv.) was also added, drop-wise, which made the clear solution turn to pale yellow. The mixture was heated to 60 °C under stirring, becoming turbid. Stirring was continued overnight at the same temperature. After 16 hours, the mixture looked like a clear yellow-orange solution. After being allowed to cool down to room temperature, it was partitioned between

Et_2O (30 mL) and water (30 mL). Upon separation, the aqueous layer was extracted with Et_2O (2 x 30 mL). The combined organic layers were washed with brine, dried over Na_2SO_4, filtered, and concentrated under vacuum. The yellow-orange crude oil was submitted to column chromatography (Biotage Isolera One flash chromatographer, 25 g SiO_2; EtOAc in hexane, 0 to 20%) to provide (3-(4-(2-azidoethyl)phenethoxy)propyl)diisopropyl((trimethylsilyl)ethynyl)silane (**S1**) (0.328 g, 0.739 mmol, 77% yield) as a colorless oil.

R_f = 0.3 (Hexane:Ethyl acetate 95:5).
^{1}H NMR (400 MHz, Chloroform-d) δ 7.21–7.16 (m, 2H, Ar*H*), 7.15–7.11 (m, 2H, Ar*H*), 3.62 (t, *J* = 7.3 Hz, 2H, C*H₂*), 3.48 (t, *J* = 7.3 Hz, 2H, C*H₂*), 3.42 (t, *J* = 6.9 Hz, 2H, C*H₂*), 2.92–2.80 (m, 4H, ArC*H₂*), 1.74–1.62 (m, 2H, C*H₂*), 1.03 (dd, *J* = 9.3, 6.6 Hz, 12H, SiCH(C*H₃*)₂), 1.00–0.92 (m, 2H, SiC*H*(CH₃)₂), 0.63–0.54 (m, 2H, SiC*H₂*), 0.16 (s, 9H, Si(C*H₃*)₃).
^{13}C NMR (101 MHz, Chloroform-d) δ 137.8, 135.9, 129.4, 128.8, 116.5, 110.1, 73.9, 71.8, 52.7, 36.2, 35.1, 24.6, 18.3, 18.1, 11.7, 6.1, 0.2.
IR (ν_{max}, cm^{-1}) 2949 (m), 2867 (m), 2097 (m), 1515 (w), 1462 (w), 1287 (w), 1250 (m), 1111 (m).
HRMS (APCI/QTOF) m/z: [M + Na]$^+$ Calcd for $C_{24}H_{41}N_3NaOSi_2^+$ 466.2680; Found 466.2674.

8-Chlorooct-1-ynyl(trimethyl)silane (S2)

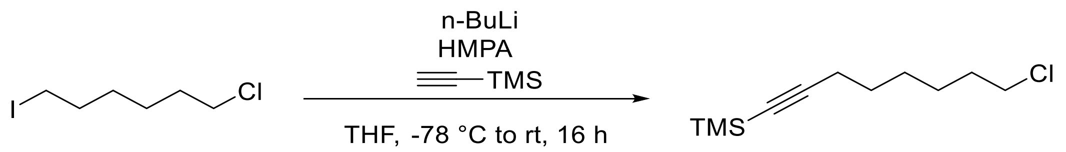

A cooled *n*BuLi solution (1.15 g, 7.20 mL, 18.0 mmol, 2.50 M, 1.20 equiv) was added dropwise to a stirred solution of TMS acetylene (1.77 g, 2.49 mL, 18.0 mmol, 1.20 equiv) in tetrahydrofuran (4 mL) at - 78 °C. After stirring for 1 hour at this temperature, a solution of 1-chloro-6-iodohexane (3.70 g, 2.28 mL, 15.0 mmol, 1.00 equiv) and HMPA (2.96 g, 2.87 mL, 16.5 mmol, 1.10 equiv) in THF (2 mL) was added dropwise. The mixture was stirred for 1 hour at - 78 °C, followed by 24 hours of stirring at room temperature. The solution was then cooled to 0 °C, quenched by a saturated aqueous solution of ammonium chloride (15 mL) and diluted with water (3 mL) and ethyl acetate (15 mL). The layers were separated and the aqueous layer extracted with ethyl acetate (3 x 15 mL). The combined organic layers were washed with water (2 x 30 mL), brine (30 mL), dried over magnesium sulfate, filtered and concentrated in vacuo. The crude liquid was purified through a plug of silica, eluting with 1% EtOAc in pentane, to afford pure 8-chlorooct-1-ynyl(trimethyl)silane (**S2**) (1.98 g, 9.12 mmol, 61% yield) as a colorless liquid.

R_f = 0.4 (Pentane).
^{1}H NMR (400 MHz, $CDCl_3$) δ 3.53 (t, *J* = 6.7 Hz, 2H, C*H₂*Cl), 2.22 (t, *J* = 7.0 Hz, 2H, CCC*H₂*), 1.78 (dq, *J* = 8.1, 6.6 Hz, 2H, CC*H₂*CH2Cl), 1.57 – 1.48 (m, 2H, C*H₂*), 1.48 – 1.35 (m, 4H, C*H₂*), 0.14 (s, 9H, TMS).
^{13}C NMR (101 MHz, $CDCl_3$) δ 107.5, 84.7, 45.1, 32.6, 28.5, 28.1, 26.5, 19.9, 0.3.
HRMS (ESI/QTOF) m/z: [M + Ag]$^+$ Calcd for $C_{11}H_{21}AgClSi^+$ 323.0146; Found 323.0152.

8-(Trimethylsilyl)oct-7-yn-1-ol (S3)

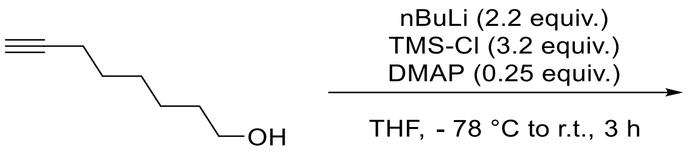

7-octyn-1-ol (3.00 g, 23.8 mmol, 1.00 equiv) was dissolved in dry tetrahydrofuran (80 mL) and the solution was cooled down at - 78 °C. A cooled solution of *n*BuLi (3.35 g, 20.9 mL, 52.3 mmol, 2.50M, 2.20 equiv) was added dropwise, followed by DMAP (726 mg, 5.94 mmol, 0.250 equiv). After stirring for 1 hour at this temperature, TMSCl (8.47 g, 9.88 mL, 78.0 mmol, 3.28 equiv) was added dropwise. The mixture was then allowed to warm to room temperature. After 2 hours of stirring, the reaction was quenched with a 1.0 N aqueous hydrochloric acid (50 mL) and vigorously stirred at room temperature over 30 minutes. The mixture was then diluted with ethyl acetate (100 mL) and the layers were separated. The aqueous layer was extracted with additional portions of ethyl acetate (3 x 50 mL). The combined organic layers were collected, washed with a solution of saturated aqueous sodium bicarbonate (100 mL), brine (50 mL), dried over magnesium sulfate, filtered and concentrated in vacuo. After purification by column chromatography (SiO_2, Pentane:Ethyl acetate 4:1), 8-(Trimethylsilyl)oct-7-yn-1-ol **(S3)** was obtained as a colorless oil.

R_f = 0.32 (Pentane:Ethyl acetate 4:1).
^{1}H NMR (400 MHz, $CDCl_3$) δ 3.64 (t, *J* = 6.6 Hz, 2H, C*H₂*OH), 2.22 (t, *J* = 7.0 Hz, 2H, CCC*H₂*), 1.66 – 1.46 (m, 4H, C*H₂*), 1.47 – 1.33 (m, 4H, C*H₂*), 0.14 (s, 9H, TMS).
^{13}C NMR (101 MHz, $CDCl_3$) δ 107.6, 84.5, 62.9, 32.7, 28.6, 28.6, 25.3, 19.9, 0.3.
HRMS (Sicrit plasma/LTQ-Orbitrap) m/z: $[M + H]^+$ Calcd for $C_{11}H_{23}OSi^+$ 199.1513; Found 199.1511.

(8-Iodooct-1-yn-1-yl)trimethylsilane (S4)

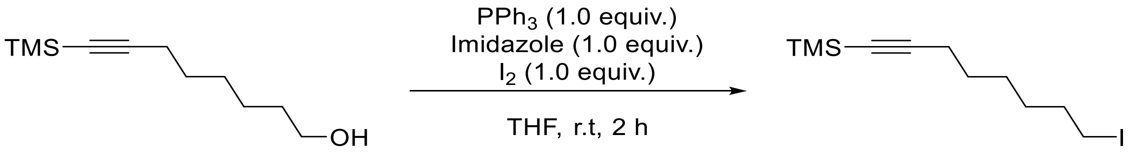

Triphenylphosphine (3.97 g, 15.1 mmol, 1.00 equiv) was added to a cooled solution of xx (3.00 g, 15.1 mmol, 1.00 equiv) in tetrahydrofuran (57 mL) at 0 °C. Upon dissolution, imidazole (1.03 g, 837 µL, 15.1 mmol, 1.00 equiv) was added, followed by I_2 (3.84 g, 15.1 mmol, 1.00 equiv). The resulting mixture was then allowed to warm to room temperature and was stirred for 2 hours. It was then diluted with diethyl ether (50 mL) and washed with 10% aqueous sodium thiosulfate (50 mL). The aqueous layer was extracted with additional portions of diethyl ether (2 x 25 mL) and the combined organic layers were washed with brine (50 mL), dried over magnesium sulfate, filtered and concentrated in vacuo. The resulting white suspension was filtered through a plug of silica, eluting with pentane (100%), to afford pure (8-Iodooct-1-yn-1-yl)trimethylsilane (**S4**)(3.06 g, 9.93 mmol, 66% yield) as a colorless oil.

R_f = 0.4 (Hexane).
^{1}H NMR (400 MHz, $CDCl_3$) δ 3.19 (t, *J* = 7.0 Hz, 2H, C*H₂*I), 2.22 (t, *J* = 7.0 Hz, 2H, CCC*H₂*), 1.83 (tdd, *J* = 7.0, 5.8, 3.2 Hz, 2H, C*H₂*), 1.52 (td, *J* = 5.9, 5.0, 3.0 Hz, 2H, C*H₂*), 1.41 (p, *J* = 3.5 Hz, 4H, C*H₂*), 0.15 (s, 9H, TMS).
^{13}C NMR (101 MHz, $CDCl_3$) δ 107.5, 84.7, 33.5, 30.1, 28.5, 27.8, 19.9, 7.1, 0.3.
HRMS (ESI/QTOF) m/z: $[M + Ag]^+$ Calcd for $C_{11}H_{21}AgISi^+$ 414.9503; Found 414.9505.

(8-Azidooct-1-yn-1-yl)trimethylsilane (S5)

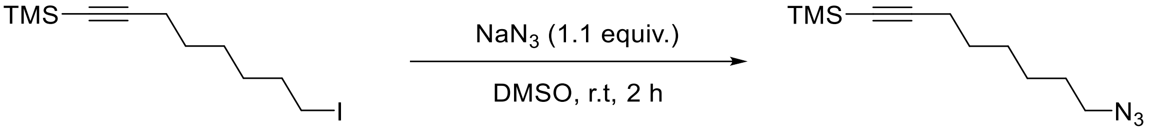

Sodium azide (738 mg, 11.4 mmol, 1.40 equiv) was partially dissolved in DMSO (22 mL). At room temperature, xx (2.50 g, 8.11 mmol, 1.00 equiv) was added to the resulting mixture. Upon stirring, the latter became slightly turbid, with apparent complete dissolution of the salt. The mixture was stirred at room temperature for 24 hours, becoming pale yellow over this time. It was then poured onto a mixture of ice and water. The aqueous layer was extracted with ether (4 x 30 mL). The combined organic layers were washed with water (2 x 30 mL), brine, dried over $MgSO_4$, filtered, and concentrated under reduced pressure. The resulting pale-yellow crude oil was submitted to silica plug (pentane) to provide (8-Azidooct-1-yn-1-yl)trimethylsilane **(S5)** (1.33 g, 5.96 mmol, 74% yield) as a colorless liquid.

$\mathbf{R_f}$ = 0.3 (Pentane).
^{1}H NMR (400 MHz, $CDCl_3$) δ 3.27 (t, *J* = 6.9 Hz, 2H), 2.23 (t, *J* = 7.0 Hz, 2H), 1.67 – 1.56 (m, 2H), 1.52 (q, *J* = 7.1 Hz, 2H), 1.47 – 1.32 (m, 4H), 0.15 (s, 9H).
^{13}C NMR (101 MHz, $CDCl_3$) δ 107.5, 84.7, 51.5, 28.9, 28.5, 28.4, 26.4, 19.9, 0.3.
IR (ν_{max}, cm^{-1}) 2939 (m), 2867 (m), 2097 (m), 1517 (w), 1462 (w), 1250 (m), 1112 (m).
HRMS (ESI/QTOF) m/z: $[M + Ag]^+$ Calcd for $C_{11}H_{21}AgN_3Si^+$ 330.0550; Found 330.0556.

b. Synthesis of EBX

(4-Azidobut-1-ynyl)-1,2-benziodoxol-3(1H)-one (2)

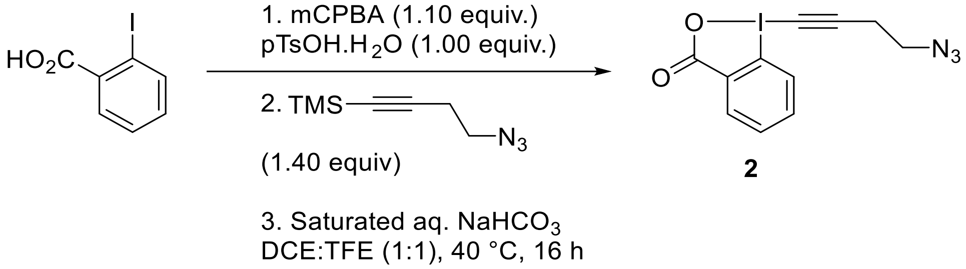

Following a reported procedure,[4] 2-iodobenzoic acid (5) (24.1 g, 97.0 mmol, 1.00 equiv.), paratoluene sulfonic acid monohydrate (pTsOH.H2O, 18.5 g, 97.0 mmol, 1.00 equiv.) and metachloroperoxybenzoic acid (mCPBA-77%, 23.9 g, 107 mmol, 1.10 equiv.) were dissolved in a mixture of dichloroethane (81 mL) and 2,2,2-trifluoroethanol (81 mL). After 1 hour stirring at 40 °C, (4-azidobut-1-yn-1-yl)trimethylsilane (S3) (22.7 g, 136 mmol, 1.40 equiv.) was added in one portion. The reaction mixture was stirred for an additional 14 hours at the same temperature, then the resulting suspension was filtered and the volatiles were removed under reduced pressure. The resultant residue was dissolved in dichloromethane (1000 mL) and treated with a solution of saturated aqueous sodium bicarbonate (1000 mL). The mixture was vigorously stirred for 1 hour, then the two layers were separated and the aqueous layer was extracted with additional portions of dichloromethane (3 x 500 mL). The organic layers were combined, dried over magnesium sulfate; filtered and concentrated under reduced

[4] Frei, R.; Wodrich, M. D.; Hari, D. P.; Borin, P.-A.; Chauvier, C.; Waser J. J. Am. Chem. Soc. 2014, 136, 16563.

pressure. Purification by column chromatography (SiO2, Ethyl acetate) afforded (4-azidobut-1-ynyl)-1,2benziodoxol-3(1H)-one (2a) (5.23 g, 15.3 mmol, 16% yield) as a white solid.

R$_f$ = 0.47 (Ethyl acetate:Methanol 9:1).
^{1}H NMR (400 MHz, $CDCl_3$) δ 8.37 (d, *J* = 7.5 Hz, 1H, ArH), 8.21 (d, *J* = 7.5 Hz, 1H, ArH), 7.80-7.70 (m, 2H, ArH), 3.56 (t, *J* = 6.5 Hz, 2H, CH2CH2N3), 2.86 (t, *J* = 6.5 Hz, 2H, CH2CH2N3).
^{13}C NMR (101 MHz, $CDCl_3$) δ 167.2, 134.9, 132.3, 131.6, 131.4, 126.8, 115.8, 104.5, 49.4, 42.7, 21.5.
Spectroscopic data was consistent with the values reported in literature.[5]

2-Iodoterephthalic acid (6)

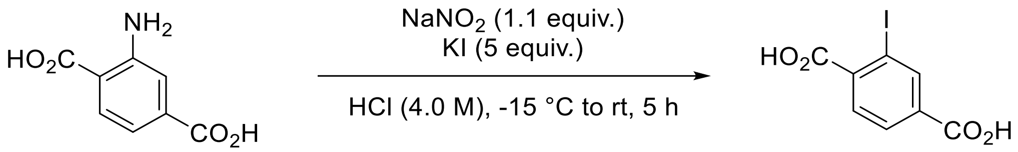

5 **6**

Following a reported procedure[6], a solution of 2-aminoterephthalic acid (**5**) (5.43 g, 30.0 mmol, 1.00 equiv) in HCl (22.8 g, 156 mL, 625 mmol, 4.00M, 20.8 equiv) was cooled in an ice-salt bath, to which was slowly added $NaNO_2$ (2.34 g, 33.9 mmol, 1.13 equiv) in 50 mL of water over 60 min with the syringe pump. The mixture was stirred for another 30 min. Then, an aqueous solution of potassium iodide (24.9 g, 150 mmol, 5.00 equiv) in 100 mL of water was cooled to -15 °C and added to the rm. The mixture was stirred at rt for 18 h. After addition of sat Na_2SO_3 (100 mL), the precipitate was collected by filtration and washed with a large amount of H_2O to obtain a 2-Iodoterephthalic acid (**6**) (6.74 g, 23.1 mmol, 77% yield) as a yellowish solid.

^{1}H NMR (400 MHz, DMSO) δ 8.42 (d, J = 1.6 Hz, 1H, Ar*H*), 7.99 (dd, J = 8.0, 1.6 Hz, 1H, Ar*H*), 7.76 (d, *J* = 8.0 Hz, 1H, Ar*H*).
HRMS (ESI/QTOF) m/z: $[M + H_{-1}]^-$ Calcd for $C_8H_4IO_4^-$ 290.9160; Found 290.9168.
Spectroscopic data was consistent with the values reported in literature.[7]

4-Oxo-2-iodosylbenzoic acid (16)

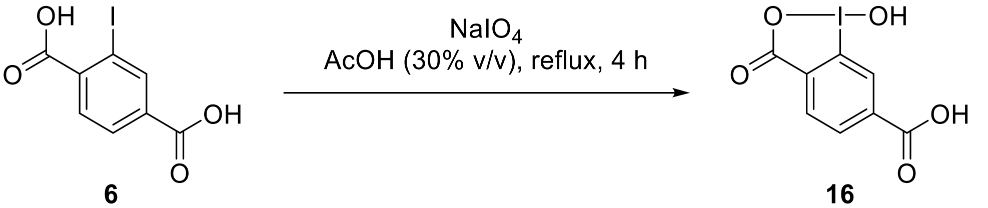

6 **16**

Following a reported procedure[6], a flask was charged with 2-iodoterephthalic acid (**6**) (2.92 g, 9.98 mmol, 1.00 equiv) and sodium periodate (2.24 g, 10.5 mmol, 1.05 equiv), acetic acid (34 mL, 30 % v:v aq.) was added under N_2 atmosphere. The resulting suspension was refluxed (110 °C) under N_2 atmosphere during 4 h with a vigorously stirring. After 4 h, the reaction was allowed to reach room temperature and transferred into an Erlenmeyer. At this point, the reaction was quenched with cold H_2O (30 mL). The obtained white solid was filtered and washed with cold H_2O (3 x 10 mL), followed by cold acetone (3 x 10 mL). The solid was dried under air overnight to obtain 4-Oxo-2-iodosylbenzoic acid

[5] Abegg, D.; Frei, R.; Cerato, L.; Hari, D. P.; Wang, C.; Waser, J.; Adibekian, A. Angew. Chem. Int. Ed. 2015, 54, 10852.
[6] A. Kommreddy, M. S. Bowsher, M. R. Gunna, K. Botha, T. K. Vinod, Tetrahedron Letters 2008, 49, 4378-4382.
[7] M. Kalaj, M. R. Momeni, K.C. Bentz, K. S. Barcus, J. M. Palomba, F. Paesani, and S. M. Cohen, Chem. Commun., 2019, 55, 3481-3484

(**16**) (2.21 g, 7.18 mmol, 72% yield) as a yellowish solid. The crude product was engaged in the next step without further purification.

^{1}H NMR (400 MHz, DMSO) δ 8.37 (d, *J* = 1.4 Hz, 1H, Ar*H*), 8.28 (s, 1H, O*H*), 8.21 (dd, *J* = 7.8, 1.4 Hz, 1H, Ar*H*), 8.10 (d, *J* = 7.9 Hz, 1H, Ar*H*).
Spectroscopic data was consistent with the values reported in literature.[6]

3-Oxo-1-((tri*iso*propylsilyl)ethynyl)-1,3-dihydro-1λ^3-benzo[*d*][1,2]iodaoxole-6-carboxylic (7a)

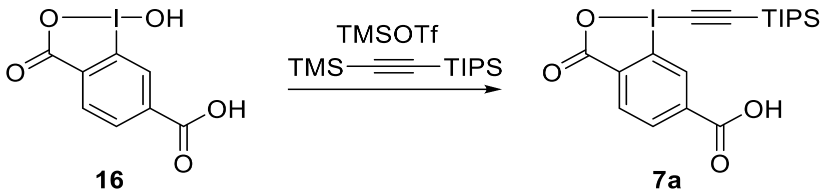

16 **7a**

Following a reported procedure[8], trimethylsilyl trifluoromethanesulfonate (3.33 g, 2.72 mL, 15.0 mmol, 5.00 equiv) was added dropwise at 0°C to a solution of **16** (924 mg, 3.00 mmol, 1.00 equiv) in dry DCM (15 mL). After 2h, trimethyl-[2-tri(propan-2-yl)silylethynyl]silane (1.15 g, 4.50 mmol, 1.50 equiv) was added dropwise. The rm was stirred at RT overnight. Then, sat. $NaHCO_3$ was added (100 mL) and the reaction mixture was stirred vigorously until pH=7, the solid washed with cold water (2 x 15 mL) and cold acetone (2 x 40 mL) and dried to obtain 3-Oxo-1-((triisopropylsilyl)ethynyl)-1,3-dihydro-1λ3-benzo[d][1,2]iodaoxole-6-carboxylic **(7a)** (893 mg, 1.89 mmol, 63% yield) as yellowish solid.

^{1}H NMR (400 MHz, MeOD) δ 9.04 (s, 1H, Ar*H*), 8.35 (d, *J* = 7.9 Hz, 1H, Ar*H*), 8.29 (d, *J* = 7.4 Hz, 1H, Ar*H*), 1.20 (d, *J* = 6.4 Hz, 21H, TIPS).
^{13}C NMR (101 MHz, DMSO) δ 166.3, 166.2, 134.2, 131.9, 130.9, 128.2, 116.2, 111.1, 66.5, 18.4, 10.7.(1C not resolved)
HRMS (ESI/QTOF) m/z: $[M + H_{-1}]^-$ Calcd for $C_{19}H_{24}IO_4Si^-$ 471.0494; Found 471.0499.

3-Oxo-1-((trimethylsilyl)ethynyl)-1,3-dihydro-1λ^3-benzo[*d*][1,2]iodaoxole-6-carboxylic (7b)

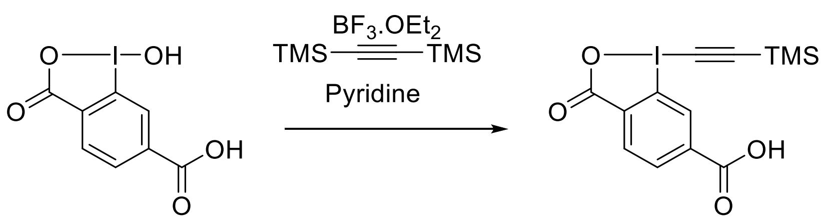

16 **7b**

$BF_3.OEt_2$ (766 mg, 570 µL, 5.40 mmol, 2.70 equiv) was added dropwise at 0°C to a solution of **16** (616 mg, 2.00 mmol, 1.00 equiv) in dry acetonitrile (28 mL). After 2 h, trimethyl(2-trimethylsilylethynyl)silane (750 mg, 4.40 mmol, 2.20 equiv) was added. The rm was stirred at RT overnight. Then, pyridine (174 mg, 177 µL, 2.20 mmol, 1.10 equiv) was added and the reaction mixture was stirred vigorously, for 2h. The solid was washed with cold water (2 x 5 mL) to obtain a white solid, 3-Oxo-1-((trimethylsilyl)ethynyl)-1,3-dihydro-1λ3-benzo[d][1,2]iodaoxole-6-carboxylic **(7b)** (192 mg, 495 µmol, 25% yield).

[8] E. Le Du, E., Ramirez, N. P., Nicolai, S., Scopelliti, R., Fadaei-Tirani, F., Wodrich, M. D., ... & Waser, J. (2023). X-Ray and NMR Structural Data of Ethynylbenziodoxolones (EBXs) Reagents and Their Analogues. Helvetica Chimica Acta, 106(3), e202200175.

^{1}H NMR (400 MHz, DMSO) δ 8.91 (d, *J* = 1.3 Hz, 1H, Ar*H*), 8.27 (dd, *J* = 7.7, 1.4 Hz, 1H, Ar*H*), 8.18 (d, *J* = 7.8 Hz, 1H, Ar*H*), 0.32 (s, 9H, TMS).
^{13}C NMR (101 MHz, DMSO) δ 165.6, 165.5, 136.5, 135.6, 132.0, 131.3, 128.4, 116.4, 114.7, 66.1, -0.6.
HRMS (ESI/QTOF) m/z: [M + H]$^+$ Calcd for $C_{13}H_{14}IO_4Si^+$ 388.9701; Found 388.9695.

1-(((3-(4-(2-Azidoethyl)phenethoxy)propyl)diisopropylsilyl)ethynyl)-3-oxo-1,3-dihydro-1λ^3-benzo[*d*][1,2]iodaoxole-6-carboxylic acid (7c)

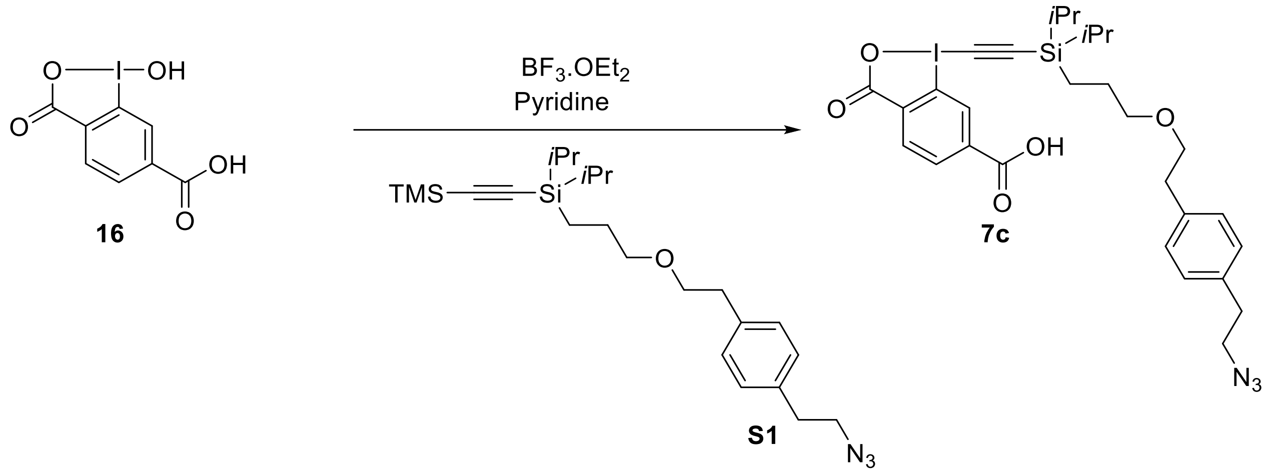

BF$_3$.OEt$_2$ (156 mg, 116 µL, 1.10 mmol, 2.20 equiv) was added dropwise at 0 °C to a solution of **16** (154 mg, 500 µmol, 1.00 equiv) in dry acetonitrile (6.0 mL). After 2h, **S1** (244 mg, 550 µmol, 1.10 equiv) was added. The rm was stirred at RT overnight. Then, pyridine (39.5 mg, 40.3 µL, 500 µmol, 1.00 equiv) was added and the reaction mixture was stirred vigorously for 2h. Then, water was added (3.0 mL) and the solid was filtered off. The filtrate was then extracted with DCM (2 mL), washed with brine and dried over MgSO$_4$. After concentration in vacuo of the organic layer, the solid was crystallized in pentane obtain a brown solid, 1-(((3-(4-(2-Azidoethyl)phenethoxy)propyl)diisopropylsilyl)ethynyl)-3-oxo-1,3-dihydro-1λ3-benzo[d][1,2]iodaoxole-6-carboxylic acid **(7c)** (95.0 mg, 144 µmol, 29% yield).

^{1}H NMR (400 MHz, DMSO) δ 8.90 (s, 1H, Ar*H*), 8.28 (d, J = 7.8 Hz, 1H, Ar*H*), 8.18 (d, J = 7.9 Hz, 1H, Ar*H*), 7.12 (s, 4H, Ar*H*), 3.51 (q, J = 6.6 Hz, 6H,C*H$_2$*-O, C*H$_2$*-N$_3$), 2.75 (dt, J = 16.1, 7.0 Hz, 4H, C*H$_2$*,benzylic), 1.70 – 1.60 (m, 2H, Si-CH$_2$-C*H$_2$*), 1.17 – 1.03 (m, 14H, Si-C*H$_2$*, iPr), 0.77 – 0.70 (m, 2H, SiC*H$_2$*).
^{13}C NMR (101 MHz, DMSO) δ 165.6, 165.5, 137.3, 136.6, 135.9, 135.6, 132.1, 131.4, 128.9, 128.6, 128.3, 116.5, 111.4, 72.3, 70.8, 66.8, 51.6, 35.2, 34.0, 24.0, 18.0, 17.8, 11.0, 5.5. (1C missing)
IR (ν_{max}, cm^{-1}) 3395 (s), 2257 (w), 2130 (w), 2098 (w), 1655 (w), 1047 (m), 1025 (s), 995 (s).
HRMS (ESI/QTOF) m/z: [M + Na]$^+$ Calcd for $C_{29}H_{36}IN_3NaO_5Si^+$ 684.1361; Found 684.1371.

3-Oxo-1-((phenylethynyl)-1,3-dihydro-1λ^3-benzo[*d*][1,2]iodaoxole-6-carboxylic (7d)

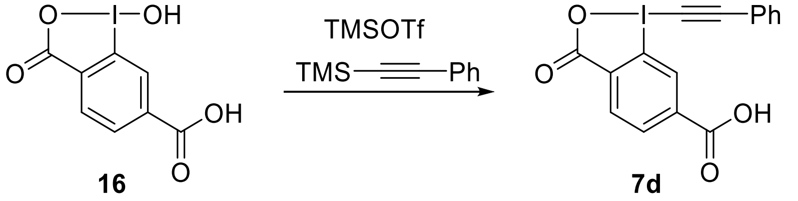

Trimethylsilyl trifluoromethanesulfonate (1.80 g, 1.47 mL, 8.12 mmol, 5.00 equiv) was added dropwise at 0°C to a solution of **16** (500 mg, 1.62 mmol, 1.00 equiv) in dry DCM (8 mL). After 2h, trimethyl(2-phenylethynyl)silane (622 mg, 703 µL, 3.57 mmol, 2.20 equiv) was added dropwise. The mixture was stirred at RT overnight. Then, 5 mL of sat. NaHCO$_3$ was added and the reaction mixture was stirred vigorously for 2h. The solid was washed with cold water (2 x 7 mL) and dried to obtain 3-Oxo-1-((phenylethynyl)-1,3-dihydro-1λ3-benzo[d][1,2]iodaoxole-6-carboxylic **(7d)** (403 mg, 1.03 mmol, 63% yield) as a yellowish solid.

^{1}**H NMR** (400 MHz, DMSO) δ 8.99 (s, 1H, Ar*H*), 8.17 (d, *J* = 7.6 Hz, 1H, Ar*H*), 8.02 (d, *J* = 7.6 Hz, 1H, Ar*H*), 7.79 – 7.70 (m, 2H, Ar*H*), 7.59 – 7.46 (m, 3H, Ar*H*).
^{13}**C NMR** (101 MHz, DMSO) δ 165.6, 136.5, 135.5, 132.3, 132.0, 131.3, 130.7, 129.1, 128.5, 120.4, 117.0, 104.6, 52.3. (1C unresolved)
HRMS (ESI/QTOF) m/z: [M + Na]$^+$ Calcd for $C_{16}H_9INaO_4^+$ 414.9438; Found 414.9431.

1-(8-Chlorooct-1-yn-1-yl)-3-oxo-1,3-dihydro-1λ^3-benzo[*d*][1,2]iodaoxole-6-carboxylic acid (7e)

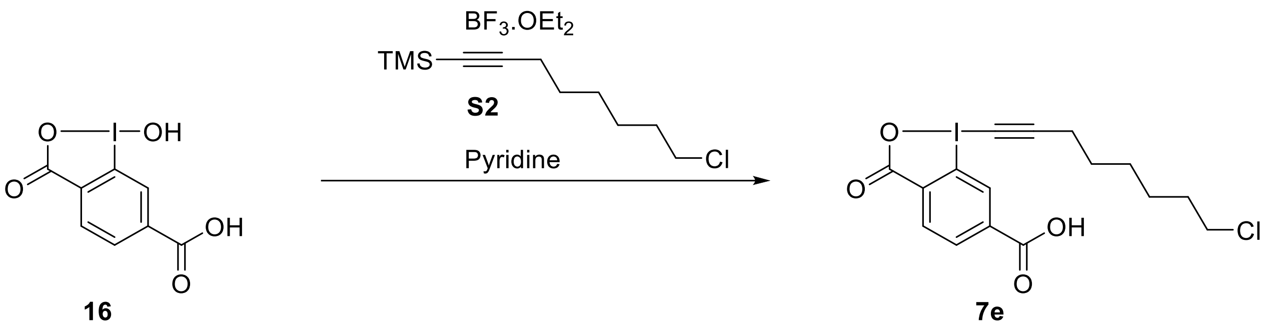

$BF_3.OEt_2$ (575 mg, 428 µL, 4.05 mmol, 2.70 equiv) was added dropwise at 0°C to a solution of **16** (462 mg, 1.50 mmol, 1.00 equiv) in dry acetonitrile (21 mL). After 2 h, **S2** (716 mg, 3.30 mmol, 2.20 equiv) was added dropwise. The rm was stirred at RT overnight. pyridine (131 mg, 133 µL, 1.65 mmol, 1.10 equiv) was added and the reaction mixture was stirred vigorously for 2h. The solid was filtered off and washed with cold water (2 x 5 mL). The filtrate was kept in a fridge after which some solid was formed. The solid was filtered off the offer 1-(8-Chlorooct-1-yn-1-yl)-3-oxo-1,3-dihydro-1λ3-benzo[d][1,2]iodaoxole-6-carboxylic acid **(7e)** (211 mg, 485 µmol, 32% yield) as a yellowish powder.

^{1}**H NMR** (400 MHz, DMSO) δ 8.82 (d, *J* = 1.3 Hz, 1H, Ar*H*), 8.27 (dd, *J* = 7.8, 1.4 Hz, 1H, Ar*H*), 8.17 (d, *J* = 7.7 Hz, 1H, Ar*H*), 3.62 (t, *J* = 6.6 Hz, 2H, C*H*$_2$Cl), 2.69 (t, *J* = 6.9 Hz, 2H, CCC*H*$_2$), 1.86 – 1.58 (m, 4H, C*H*$_2$), 1.45 (tdd, *J* = 13.5, 6.5, 2.6 Hz, 4H, C*H*$_2$).
^{13}**C NMR** (101 MHz, DMSO) δ 165.6, 165.4, 136.4, 135.7, 132.0, 131.2, 128.3, 116.4, 108.4, 45.3, 31.9, 27.5, 27.5, 25.7, 19.6. (1C not resolved)
HRMS (Sicrit plasma/LTQ-Orbitrap) m/z: [M + H]$^+$ Calcd for $C_{16}H_{17}ClIO_4^+$ 434.9855; Found 434.9853.

1-(8-Azidooct-1-yn-1-yl)-3-oxo-1,3-dihydro-1λ^3-benzo[*d*][1,2]iodaoxole-6-carboxylic acid (7f)

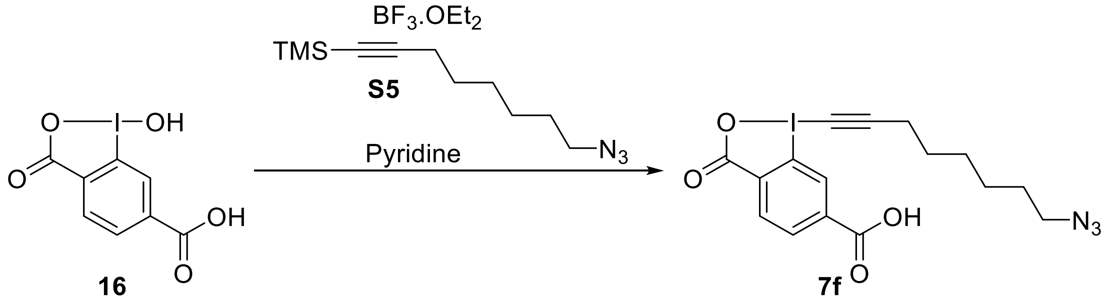

$BF_3.OEt_2$ (575 mg, 428 µL, 4.05 mmol, 2.70 equiv) was added dropwise at 0 °C to a solution of **16** (462 mg, 1.50 mmol, 1.00 equiv) in dry acetonitrile (21 mL). After 2 h, **S5** (737 mg, 3.30 mmol, 2.20 equiv) was added dropwise. The rm was stirred at RT overnight. Then, pyridine (131 mg, 133 µL, 1.65 mmol, 1.10 equiv) was added and the reaction mixture was stirred vigorously for 2h. The solid was filtered off and washed with cold water (2 x 5 mL). The filtrate was kept in a fridge after which some solid was

formed. The solid was filtered off the offer 1-(8-Azidooct-1-yn-1-yl)-3-oxo-1,3-dihydro-1λ3-benzo[d][1,2]iodaoxole-6-carboxylic acid (**7f**) (163 mg, 369 µmol, 25% yield) as a yellowish powder.

^{1}H NMR (400 MHz, DMSO) δ 8.81 (s, 1H, Ar*H*), 8.27 (d, *J* = 7.8 Hz, 1H, Ar*H*), 8.17 (d, *J* = 7.7 Hz, 1H, Ar*H*), 3.30 (t, *J* = 6.8 Hz, 2H, C$H_2$$N_3$), 2.68 (t, *J* = 6.9 Hz, 2H, CCCH_2), 1.64 (p, *J* = 7.0 Hz, 2H, CH_2), 1.51 (ddt, *J* = 23.1, 14.8, 6.9 Hz, 4H, CH_2), 1.37 (p, *J* = 7.4, 7.0 Hz, 2H, CH_2).
^{13}C NMR (101 MHz, DMSO) δ 165.6, 165.4, 136.5, 135.7, 132.0, 131.2, 128.2, 116.3, 108.3, 50.6, 28.0, 27.7, 27.5, 25.6, 19.6. (1C not resolved)
IR (ν_{max}, cm^{-1}) 3392 (s), 2261 (w), 2128 (w), 2098 (w), 1644 (w), 1043 (m), 1025 (s), 995 (s).
HRMS (ESI/QTOF) m/z: $[M + H]^+$ Calcd for $C_{16}H_{17}IN_3O_4^+$ 442.0258; Found 442.0260.

c. Synthesis of EBX peptide

General procedure A:

In a test tube, EBX (50 µmol, 1 equiv.) and T3P (1 equiv.) were dissolved in DMF (2 mL). Then, DIPEA (250 µmol, 5 equiv.) was added and the mixture was added to the Trt-resin (on 100 µmol scale, loading of the resin 1.4 mmol/g). The syringe was shaken for 2 h. Then, the EBX was cleaved off the resin with a mixture TFA/H_2O/TIPS: 95:2.5:2.5 for 2 h on the shaker. The solution was poured into a falcon filled with 45mL of cold ether. Some precipitation was observed. The falcon was centrifuged to remove the ether, and the residues were dried with N_2 flow.

General procedure A1:

In a test tube, EBX (150 µmol, 1.50 equiv.) and HATU (1.10 equiv) were dissolved in DMF (2 mL). Then, DIPEA (500 µmol, 5 equiv) was added and the mixture was added to the Trt-resin (on 100 µmol scale, loading of the resin 1.4 mmol/g). The syringe was shaken for 2 h. Then, the EBX was cleaved off the resin with a mixture TFA/H_2O/TIPS: 95:2.5:2.5 for 2 h on the shaker. The solution was poured into a falcon filled with 45mL of cold ether. Some precipitation was observed. The falcon was centrifuged to remove the ether, and the residues were dried with N_2 flow.

General procedure B:

Removal of Mtt:
The resin was treated with 2 mL of DCM/TFA/TIPS (93:2:5) for 8 times each time 2 min (until the solution is colourless). Then, the resin was washed with DCM (3 x 2 mL) and a 5% DIPEA solution in DMF (2 mL).

Coupling:
In a test tube, EBX (100 µmol, 1.00 equiv) and coupling reagent were dissolved in DMF (2 mL). Then, DIPEA (500 µmol, 5 equiv) was added and the mixture was added to TentaGel-resin (on 100 µmol scale). The syringe was shaken for 2 h. Then, the EBX was cleaved off the resin with a mixture TFA/H_2O/TIPS: 95:2.5:2.5 for 2 h on the shaker. The solution was poured into a falcon filled with 45 mL of cold ether. Some precipitation was observed. The falcon was centrifuged to remove the ether, and the residues were dried with a N_2 flow.

EBX **4aa**

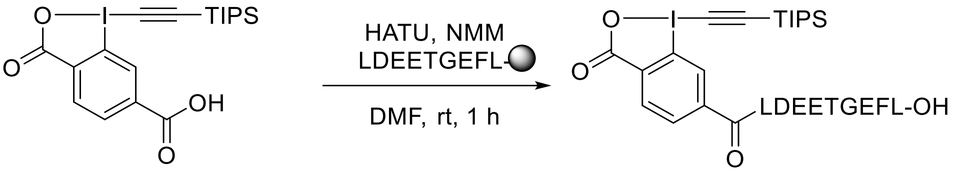

Following the general procedure A1 using HATU (41.8 mg, 110 μmol, 1.10 equiv) with 2Cl-Trt resin on 100 μmol scale, the desired product (TIPS)EBX-LDEETGEFL-OH (**4aa**) (16.3 mg, 10.6 μmol, 11% based on resin loading) was isolated by **Method 2**.

HRMS (nanochip-ESI/LTQ-Orbitrap) m/z: $[M + H_2]^{+2}$ Calcd for $C_{65}H_{94}IN_9O_{22}Si^{+2}$ 753.7658; Found 753.7642.

MS/MS characterization:

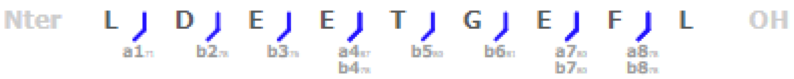

Nter = C19H24IO3Si

Sequence	Type	MF	MF Mass	m/z	Similarity	Qty
LDEE	a4	C38H54IN4O12Si(+1)	913.2552	913.2547	87.24%	0.07%
LDEETG	b6	C45H64IN6O16Si(+1)	1099.319	1099.319	80.88%	1.72%
LDEETGE	a7	C49H71IN7O18Si(+1)	1200.367	1200.366	80.49%	3.73%
LDEETGE	b7	C50H71IN7O19Si(+1)	1228.362	1228.361	80.33%	27.84%
LDEET	b5	C43H61IN5O15Si(+1)	1042.298	1042.297	79.78%	2.58%
LDEE	b4	C39H54IN4O13Si(+1)	941.2501	941.2496	78.30%	3.89%
LD	b2	C29H40IN2O7Si(+1)	683.165	683.1644	78.28%	0.23%
LDEETGEF	a8	C58H80IN8O19Si(+1)	1347.435	1347.435	78.26%	3.77%
LDEETGEF	b8	C59H80IN8O20Si(+1)	1375.43	1375.43	78.07%	21.58%
LDE	b3	C34H47IN3O10Si(+1)	812.2075	812.207	76.24%	0.87%
L	a1	C24H35INO3Si(+1)	540.1431	540.1425	71.07%	0.12%

HPLC-UV chromatogram (254nm)

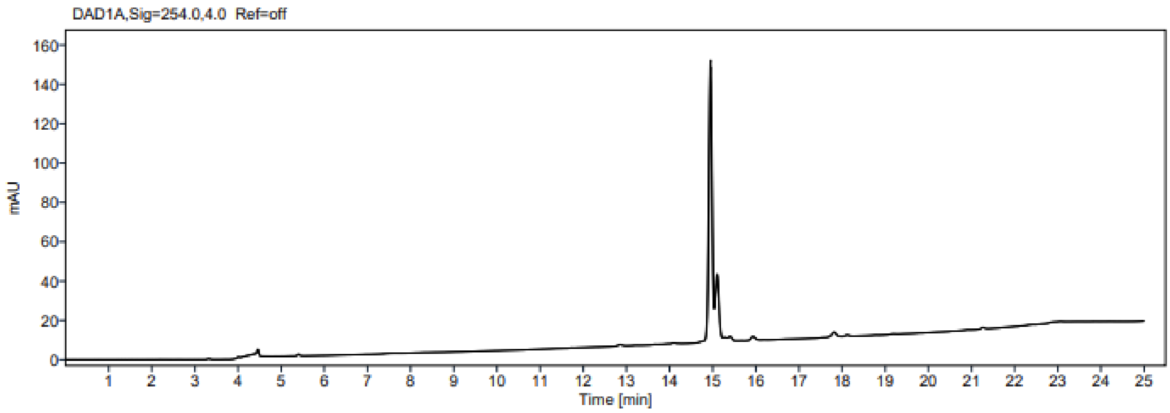

HPLC-MS chromatograms, MS1 -TIC scan

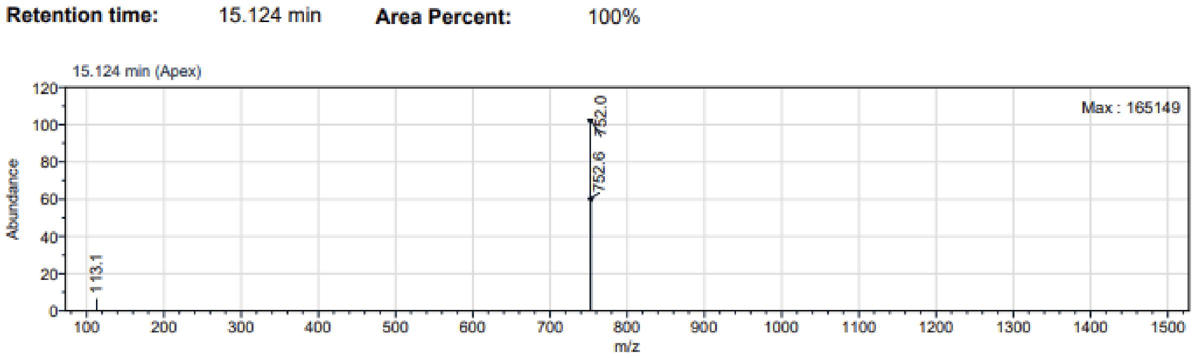

*Figure S1. HPLC and MS analysis of EBX **4aa***

EBX **4ab**

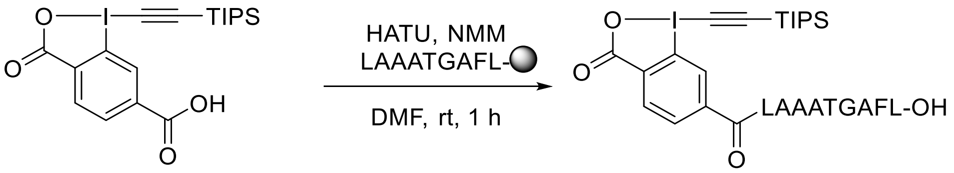

Following the general procedure A1 using HATU (41.8 mg, 110 µmol, 1.10 equiv) with 2Cl-Trt resin on 100 µmol scale, the desired product (TIPS)EBX-LAAATGAFL-OH (**4ab**) (12 mg, 9.3 µmol, 9% based on resin loading) was isolated by **Method 2**.

HRMS (nanochip-ESI/LTQ-Orbitrap) m/z: $[M + H]^+$ Calcd for $C_{58}H_{87}IN_9O_{14}Si^+$ 1288.5181; Found 1288.5206.

MS/MS characterization:

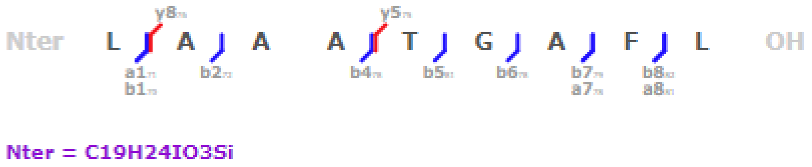

Sequence	Type	MF	MF Mass	m/z	Similarity	Qty
LAAATGAF	b8	C52H74IN8O12Si(+1)	1157.424	1157.424	81.80%	18.34%
LAAAT	b5	C38H57IN5O9Si(+1)	882.297	882.2965	81.14%	1.07%
LAAATGAF	a8	C51H74IN8O11Si(+1)	1129.429	1129.429	80.52%	10.58%
LAAATGA	b7	C43H65IN7O11Si(+1)	1010.356	1010.355	78.86%	22.40%
LAAA	b4	C34H50IN4O7Si(+1)	781.2493	781.2488	77.87%	1.85%
LAAATGA	a7	C42H65IN7O10Si(+1)	982.3607	982.3601	77.69%	4.65%
LAAATG	b6	C40H60IN6O10Si(+1)	939.3185	939.3179	77.56%	8.34%
AAATGAFL	y8	C33H53N8O10(+1)	721.3885	721.3879	75.68%	0.01%
TGAFL	y5	C24H38N5O7(+1)	508.2771	508.2766	74.72%	0.01%
LA	b2	C28H40IN2O5Si(+1)	639.1751	639.1746	71.61%	0.19%
L	a1	C24H35INO3Si(+1)	540.1431	540.1425	71.07%	0.04%
L	b1	C25H35INO4Si(+1)	568.138	568.1375	70.47%	0.07%

HPLC-UV chromatogram (254nm)

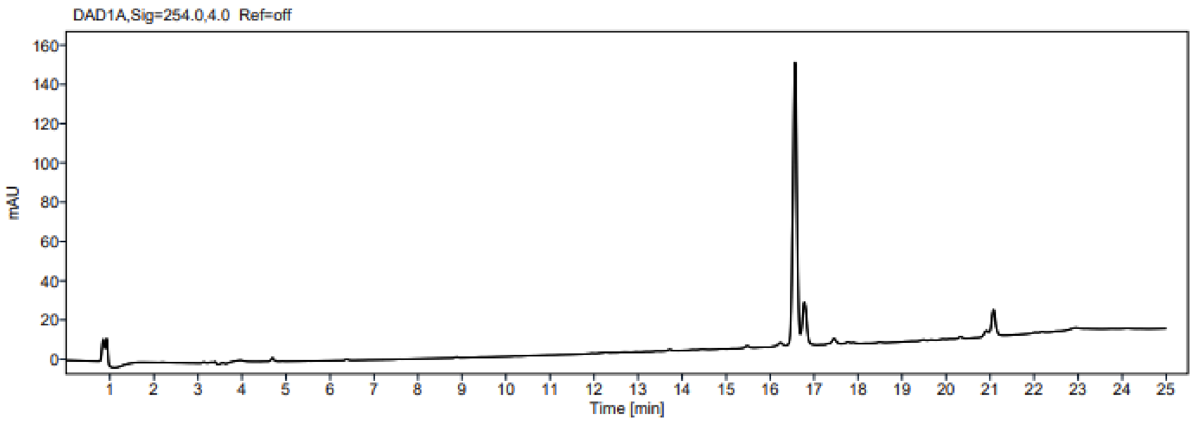

HPLC-MS chromatograms, MS1 +TIC scan

Retention time: 16.626 min **Area Percent:** 100%

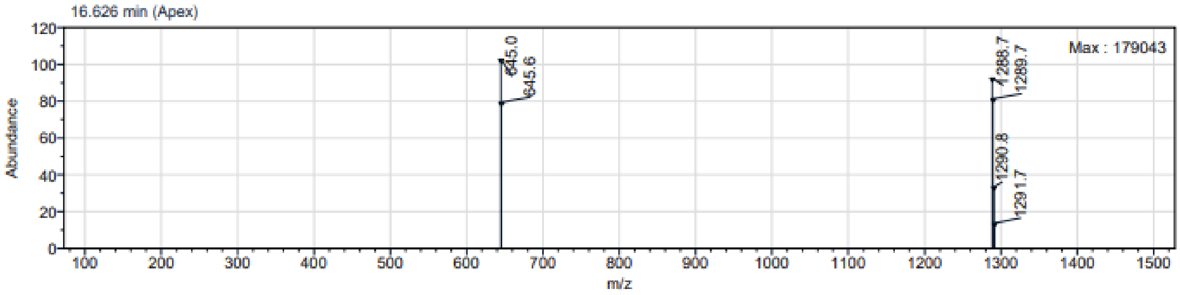

Figure S2. HPLC and MS analysis of EBX ***4ab***

EBX **4ac**

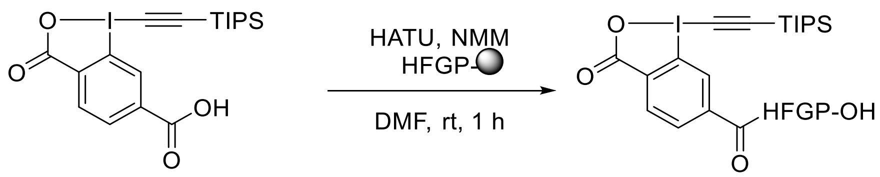

Following the general procedure A1 using HATU (83.6 mg, 220 µmol, 1.10 equiv) with 2Cl-Trt resin on 200 µmol scale, the desired product (TIPS)EBX-HFGP-OH (**4ac**) (14 mg, 15 µmol, 8% based on resin loading) was isolated by **Method 2**.

HRMS (ESI/QTOF) m/z: $[M + H_{-1}]^-$ Calcd for $C_{41}H_{50}IN_6O_8Si^-$ 909.2510; Found 909.2509.
HPLC-UV chromatogram (254nm)

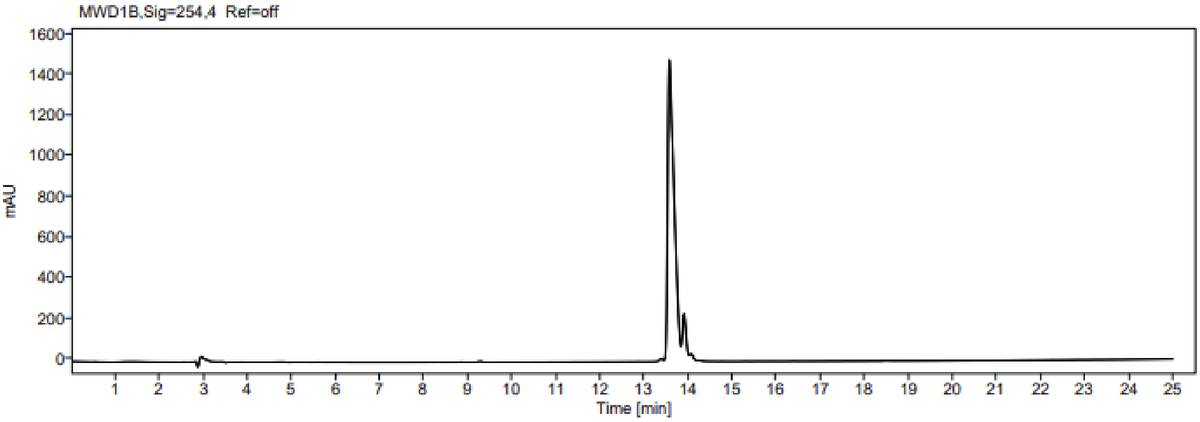

HPLC-MS chromatograms, MS1 +TIC scan

Retention time: 13.812 min **Area Percent:** 100%

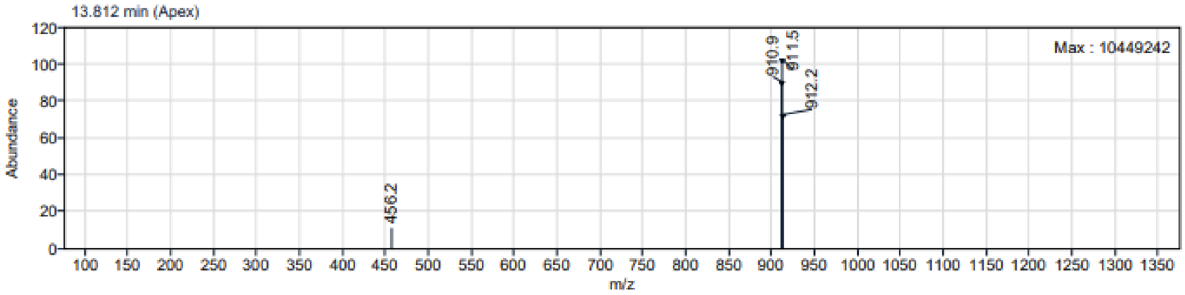

*Figure S3. HPLC and MS analysis of EBX **4ac***

EBX **4b**

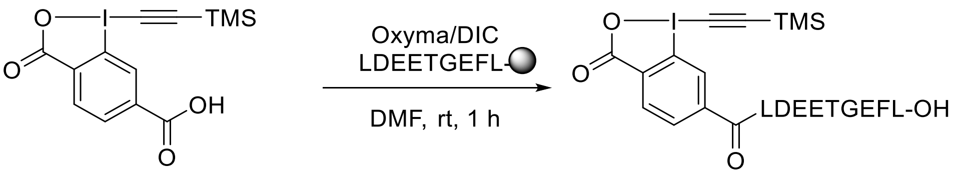

Following the general procedure A using Oxyma (14.2 mg, 100 µmol, 1.00 equiv) and DIC (12.6 mg, 15.7 µL, 100 µmol, 1.00 equiv), without base, with 2-Cl-Trt resin on 100 µmol scale, the desired product (TMS)EBX-LDEETGEFL-OH (**4b**) (7.1 mg, 4.9 µmol, 5% based on resin loading) was isolated by **Method 2**.

HRMS (nanochip-ESI/LTQ-Orbitrap) m/z: $[M + H]^+$ Calcd for $C_{59}H_{81}IN_9O_{22}Si^+$ 1422.4305; Found 1422.4322.

MS/MS characterization:

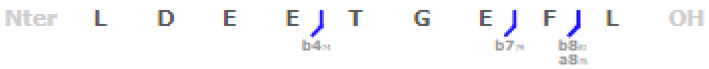

Nter = C13H12IO3Si

Sequence	Type	MF	MF Mass	m/z	Similarity	Qty
LDEETGEF	b8	C53H68IN8O20Si(+1)	1291.336	1291.336	80.53%	17.96%
LDEETGE	b7	C44H59IN7O19Si(+1)	1144.268	1144.267	78.56%	15.75%
LDEETGEF	a8	C52H68IN8O19Si(+1)	1263.342	1263.341	75.40%	3.23%
LDEE	b4	C33H42IN4O13Si(+1)	857.1562	857.1557	73.66%	1.21%

HPLC-UV chromatogram (254nm)

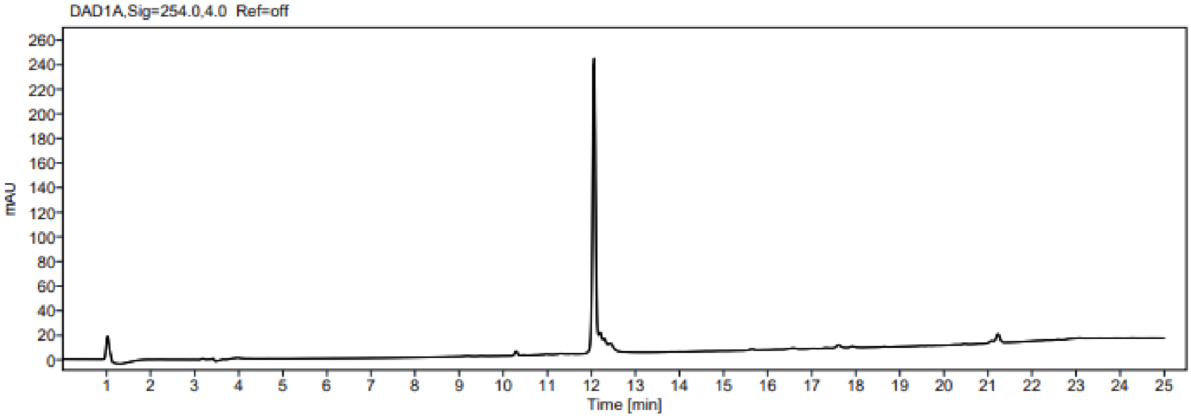

HPLC-MS chromatograms, MS1 +TIC

Retention time: 12.126 min **Area Percent:** 100%

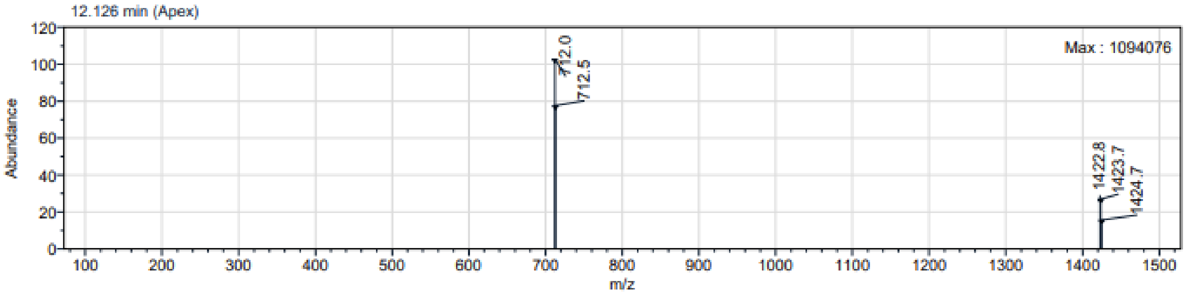

*Figure S4. HPLC and MS analysis of EBX **4b***

EBX **4ca**

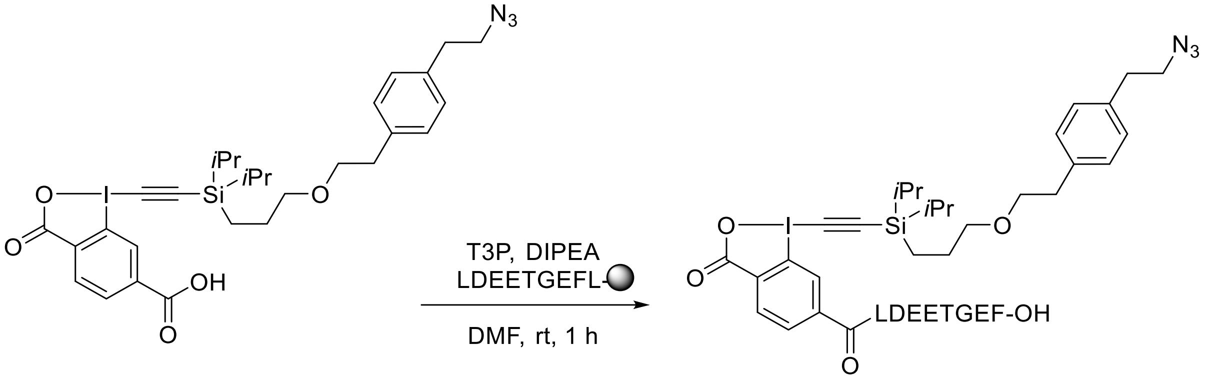

Following the general procedure A using T3P (31.8 mg, 29.2 µL, 1.00 equiv) with 2Cl-Trt resin on 100 µmol scale, the desired product (TIPS-N_3)EBX-LDEETGEFL-OH (**4ca**) (5.3 mg, 3.1 µmol, 6% based on EBX) was isolated by **Method 2**.

HRMS (nanochip-ESI/LTQ-Orbitrap) m/z: $[M + H]^+$ Calcd for $C_{75}H_{104}IN_{12}O_{23}Si^+$ 1695.6146; Found 1695.6175.

MS/MS characterization:

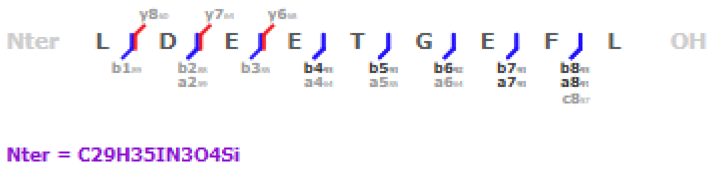

Sequence	Type	MF	MF Mass	m/z	Similarity	Qty
LDEETGEF	b8	C69H91IN11O21Si(+1)	1564.521	1564.52	93.45%	26.68%
LDEET	b5	C53H72IN8O16Si(+1)	1231.388	1231.388	93.29%	2.47%
LDEETGE	b7	C60H82IN10O20Si(+1)	1417.452	1417.452	93.20%	30.33%
LDEETGE	a7	C59H82IN10O19Si(+1)	1389.457	1389.457	92.92%	3.57%
LDEE	b4	C49H65IN7O14Si(+1)	1130.34	1130.34	92.69%	3.13%
LDEETG	b6	C55H75IN9O17Si(+1)	1288.41	1288.409	91.64%	1.59%
LDEETGEF	a8	C68H91IN11O20Si(+1)	1536.526	1536.525	91.05%	5.57%
L	b1	C35H46IN4O5Si(+1)	757.2282	757.2277	89.29%	0.07%
LD	b2	C39H51IN5O8Si(+1)	872.2552	872.2546	87.53%	0.15%
LDEET	a5	C52H72IN8O15Si(+1)	1203.393	1203.393	85.41%	0.11%
LDE	b3	C44H58IN6O11Si(+1)	1001.298	1001.297	84.89%	0.59%
ETGEFL	y6	C31H47N6O12(+1)	695.3252	695.3246	67.84%	0.01%
LDEE	a4	C48H65IN7O13Si(+1)	1102.345	1102.345	63.90%	0.05%
EETGEFL	y7	C36H54N7O15(+1)	824.3678	824.3672	63.62%	0.01%
LDEETG	a6	C54H75IN9O16Si(+1)	1260.415	1260.414	63.51%	0.06%
DEETGEFL	y8	C40H59N8O18(+1)	939.3947	939.3942	60.33%	0.01%
LD	a2	C38H51IN5O7Si(+1)	844.2602	844.2597	59.11%	0.03%
LDEETGEF	c8	C69H94IN12O21Si(+1)	1581.547	791.2769	56.98%	0.03%
LDEETGEF	c8	C69H94IN12O21Si(+1)	1581.547	1581.547	56.20%	0.14%

HPLC-UV chromatogram (254nm)

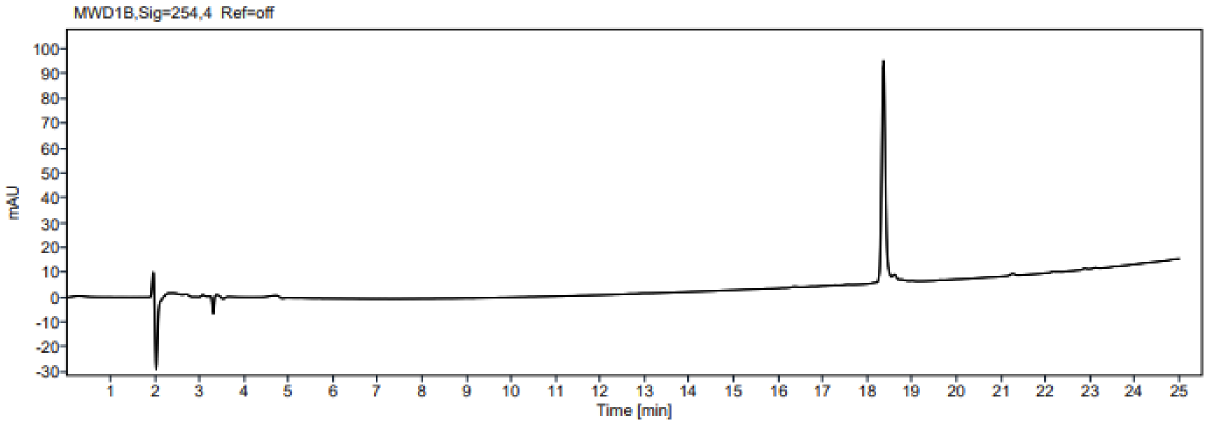

HPLC-MS chromatograms, MS1 +TIC

Retention time: 18.388 min **Area Percent:** 100%

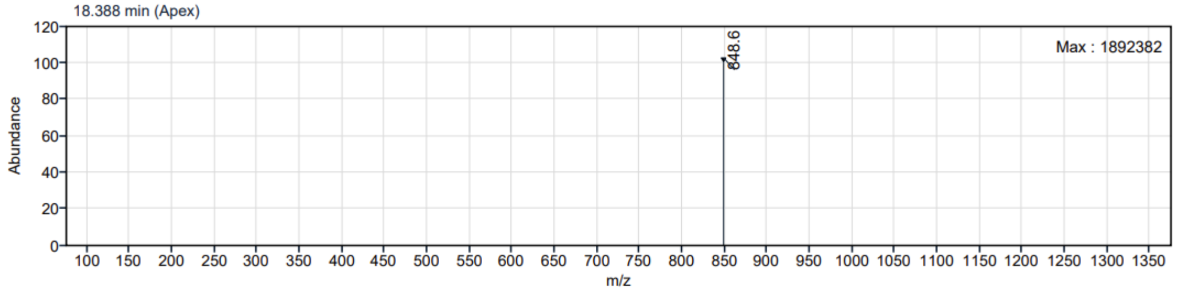

Figure S5. HPLC and MS analysis of EBX ***4ca***

EBX **4cb**

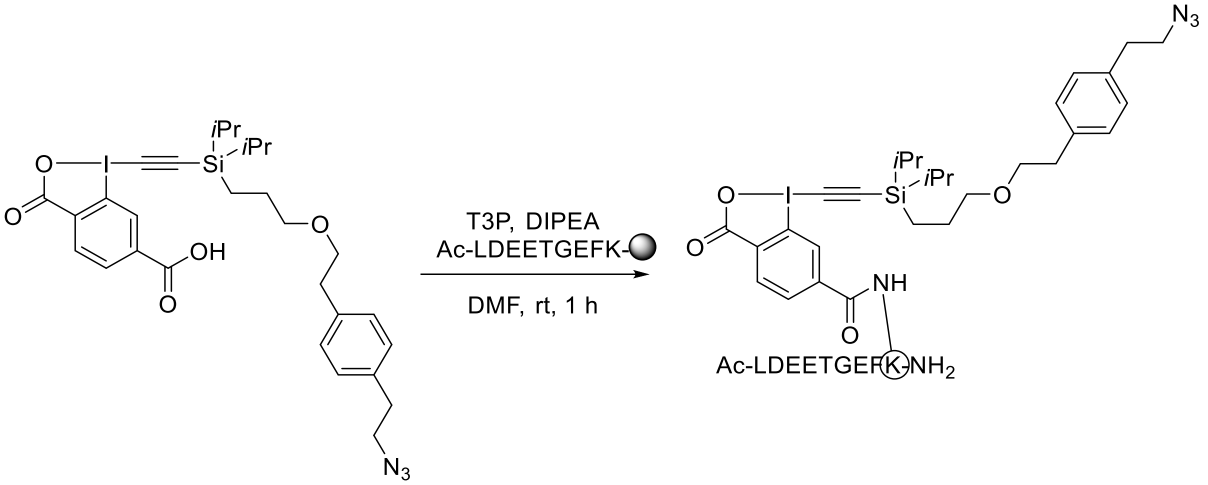

Following the general procedure B using T3P (31.8 mg, 29.2 µL, 1.00 equiv) with TentaGel resin on 100 µmol scale, the desired product Ac-LDEETGEFK(TIPS-N_3)EBX-NH_2 (**4cb**) (1.3 mg, 0.57 µmol, 1% based on EBX) was isolated by **Method 2**.

HRMS (nanochip-ESI/LTQ-Orbitrap) m/z: $[M + H]^+$ Calcd for $C_{77}H_{108}IN_{14}O_{23}Si^+$ 1751.6520; Found 1751.6544.

HPLC-UV chromatogram (254nm)

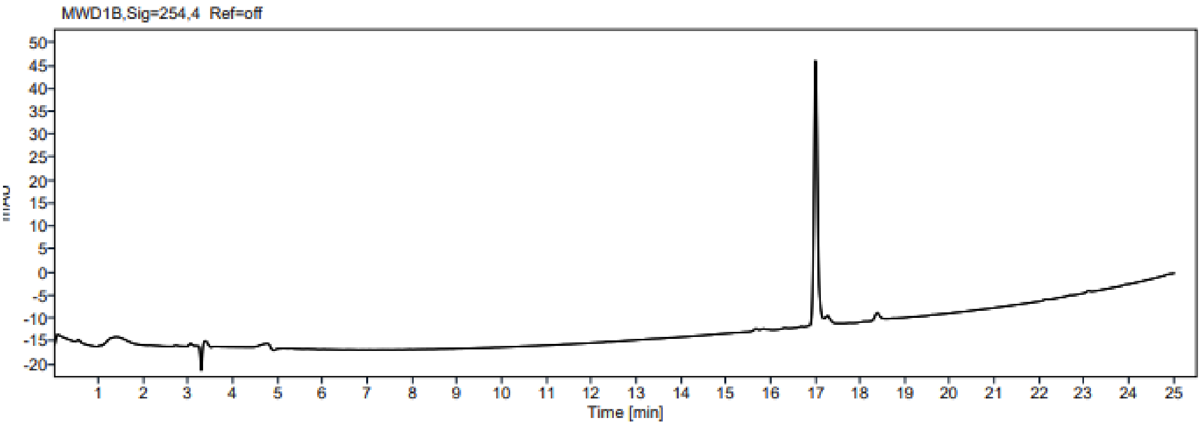

HPLC-MS chromatograms, MS1 +TIC scan

Retention time: 17.045 min **Area Percent:** 100%

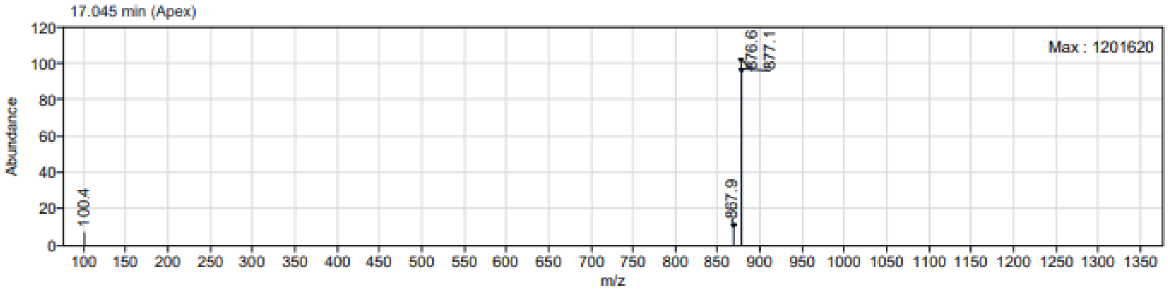

Figure S6. HPLC and MS analysis of EBX ***4cb***

EBX **4d**

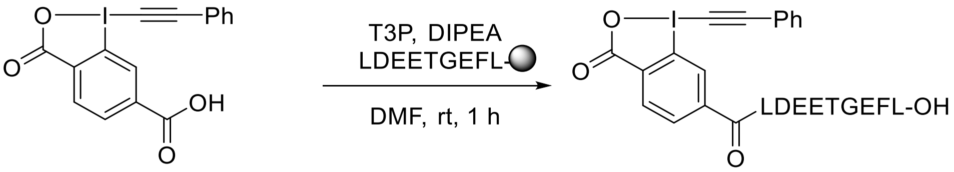

Following the general procedure A using T3P (31.8 mg, 29.2 µL, 1.00 equiv) with 2Cl-Trt resin on 100 µmol scale, the desired product (Ph)EBX-LDEETGEFL-OH (**4d**) (3.2 mg, 2.1 µmol, 2% based on EBX) was isolated by **Method 2**.

HRMS (nanochip-ESI/LTQ-Orbitrap) m/z: $[M + H]^+$ Calcd for $C_{62}H_{77}IN_9O_{22}^+$ 1426.4222; Found 1426.4265.

MS/MS characterization:

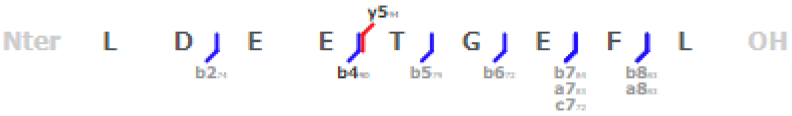

Nter = C16H8IO3

Sequence	Type	MF	MF Mass	m/z	Similarity	Qty
TGEFL	y5	C26H40N5O9(+1)	566.2826	566.2821	93.77%	0.07%
LDEE	b4	C36H38IN4O13(+1)	861.148	861.1475	90.06%	3.68%
LDEETGE	b7	C47H55IN7O19(+1)	1148.2597	1148.2592	83.77%	19.99%
LDEETGE	a7	C46H55IN7O18(+1)	1120.2648	1120.2643	83.34%	2.11%
LDEETGEF	b8	C56H64IN8O20(+1)	1295.3282	1295.3276	83.11%	19.18%
LDEETGEF	a8	C55H64IN8O19(+1)	1267.3332	1267.3327	82.59%	4.12%
LDEET	b5	C40H45IN5O15(+1)	962.1957	962.1951	79.13%	1.54%
LD	b2	C26H24IN2O7(+1)	603.0628	603.0623	74.48%	0.02%
LDEETGE	c7	C47H58IN8O19(+1)	1165.2863	1165.2857	72.15%	0.10%
LDEETG	b6	C42H48IN6O16(+1)	1019.2172	1019.2166	71.96%	1.12%

HPLC-UV chromatogram (254nm)

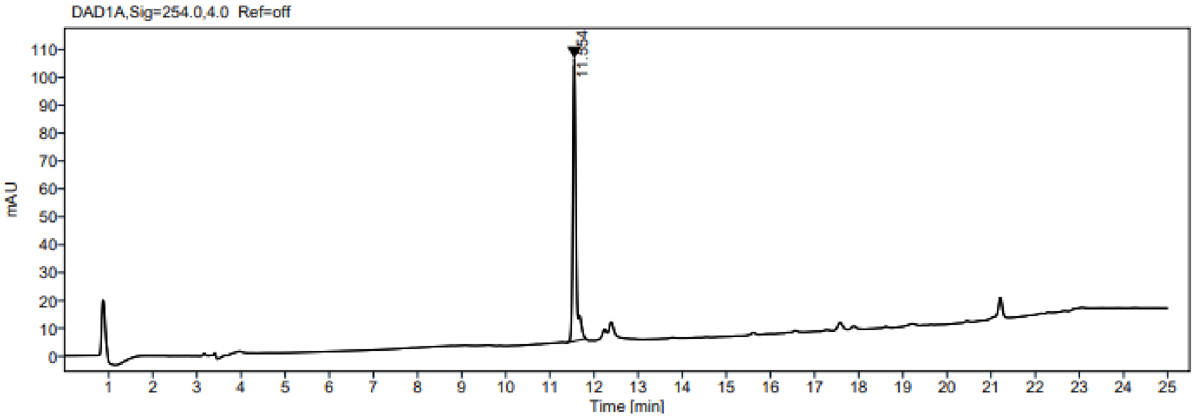

HPLC-MS chromatograms, MS1 +TIC scan

Retention time: 11.593 min **Area Percent:** 100%

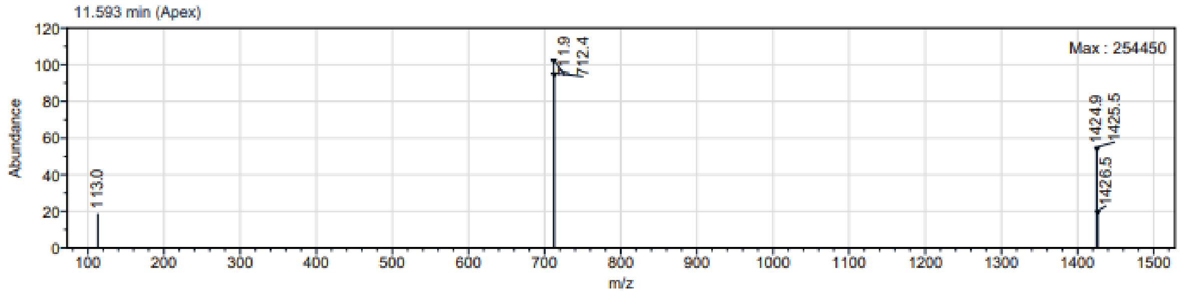

Figure S7. HPLC and MS analysis of EBX ***4d***

EBX **4e**

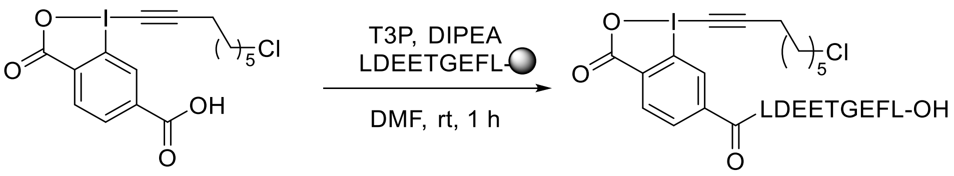

Following the general procedure A using T3P (31.8 mg, 29.2 µL, 1.00 equiv) with 2Cl-Trt resin on 100 µmol scale, the desired product (Chloro)EBX-LDEETGEFL-OH (**4e**) (7.3 mg, 4.8 µmol, 5% based on EBX) was isolated by **Method 2**.

HRMS (nanochip-ESI/LTQ-Orbitrap) m/z: $[M + H]^+$ Calcd for $C_{62}H_{84}ClIN_9O_{22}^+$ 1468.4459; Found 1468.4468.

MS/MS characterization:

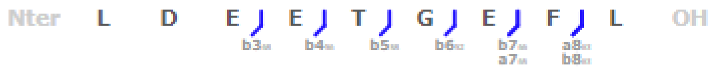

Nter = C16H15IO3Cl

Sequence	Type	MF	MF Mass	m/z	Similarity	Qty
LDEETGE	b7	C47H62ClIN7O19(+1)	1190.283	1190.283	65.89%	15.65%
LDEETGE	a7	C46H62ClIN7O18(+1)	1162.289	1162.288	65.44%	1.58%
LDEETGEF	a8	C55H71ClIN8O19(+1)	1309.357	1309.356	63.38%	3.42%
LDEETGEF	b8	C56H71ClIN8O20(+1)	1337.352	1337.351	62.99%	15.16%
LDE	b3	C31H38ClIN3O10(+1)	774.129	774.1285	61.19%	0.21%
LDEET	b5	C40H52ClIN5O15(+1)	1004.219	1004.219	57.62%	1.26%
LDEE	b4	C36H45ClIN4O13(+1)	903.1716	903.1711	55.62%	1.12%
LDEETG	b6	C42H55ClIN6O16(+1)	1061.241	1061.24	52.36%	0.60%

HPLC-UV chromatogram (210nm)

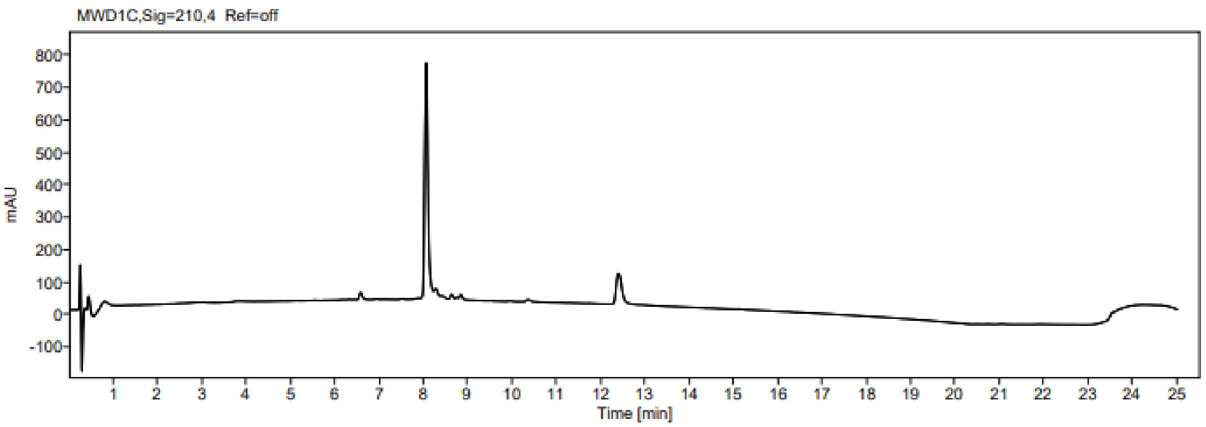

HPLC-MS chromatograms, MS1 +TIC scan

Retention time: 8.099 min **Area Percent:** 100%

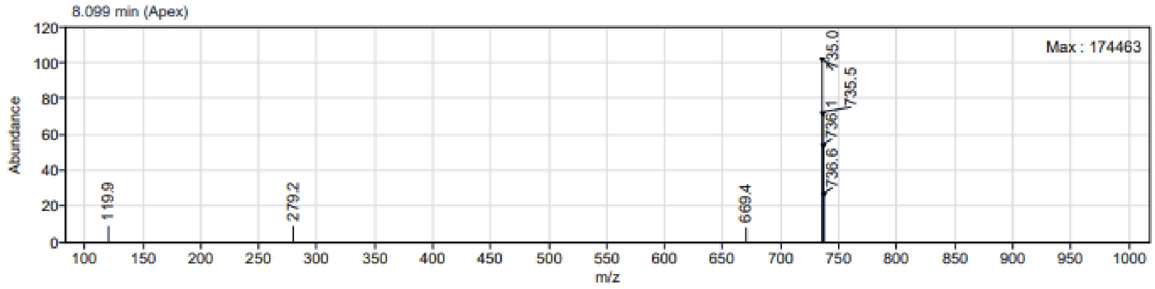

*Figure S8. HPLC and MS analysis of EBX **4e***

EBX **4f**

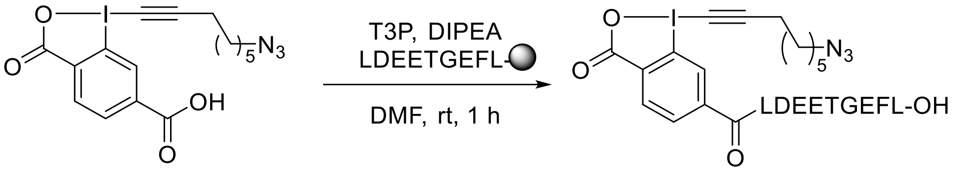

Following the general procedure A using T3P (31.8 mg, 29.2 µL, 0.5 equiv) with 2Cl-Trt resin on 100 µmol scale, the desired product (Azido)EBX-LDEETGEFL-OH (**4f**) (7.2 mg, 4.7 µmol, 5% based on EBX) was isolated by **Method 2**.

HRMS (nanochip-ESI/LTQ-Orbitrap) m/z: $[M + H_2]^{+2}$ Calcd for $C_{62}H_{85}IN_{12}O_{22}^{+2}$ 738.2468; Found 738.2500.

MS/MS characterization:

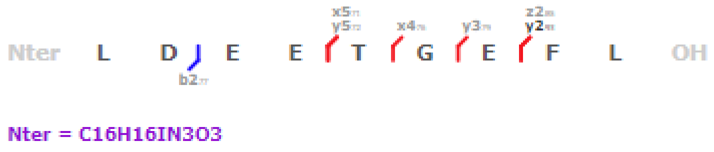

Sequence	Type	MF	MF Mass	m/z	Similarity	Qty
FL	y2	C15H23N2O3(+1)	279.1709	279.1703	93.01%	3.38%
FL	z2	C15H20NO3(+1)	262.1443	131.5755	85.05%	0.03%
EFL	y3	C20H30N3O6(+1)	408.2135	408.2129	78.81%	0.03%
L	b2	C22H27IN4O4(+1)	538.1077	135.2822	77.07%	0.02%
GEFL	x4	C23H31N4O8(+1)	491.2142	246.1105	75.72%	0.03%
LDEETGEFL		C62H84IN12O22	1475.487	738.7507	73.51%	1.54%
TGEFL	y5	C26H40N5O9(+1)	566.2826	566.2821	72.38%	0.03%
TGEFL	x5	C27H38N5O10(+1)	592.2619	99.5496	71.49%	0.02%

HPLC-UV chromatogram (254nm)

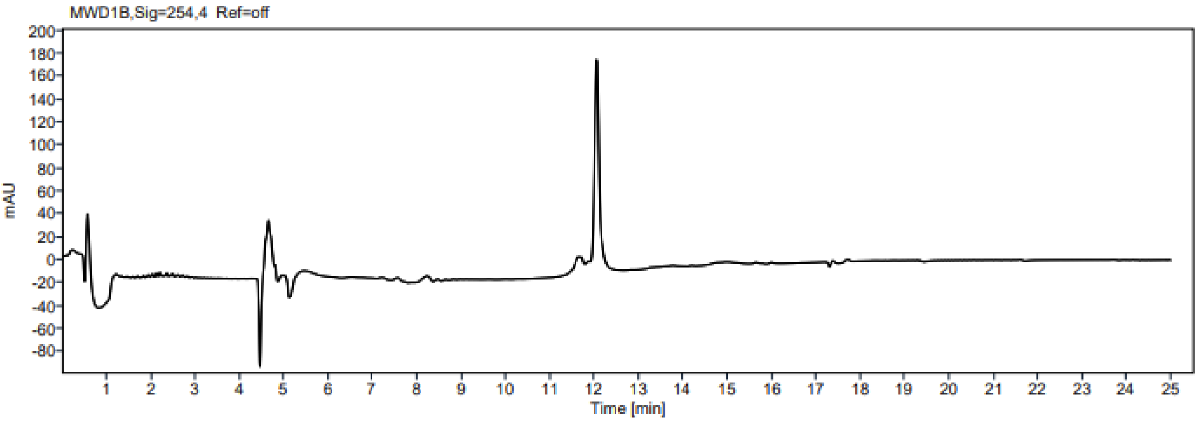

HPLC-MS chromatograms, MS1 -TIC scan

Retention time: 12.13 min **Area Percent:** 89%

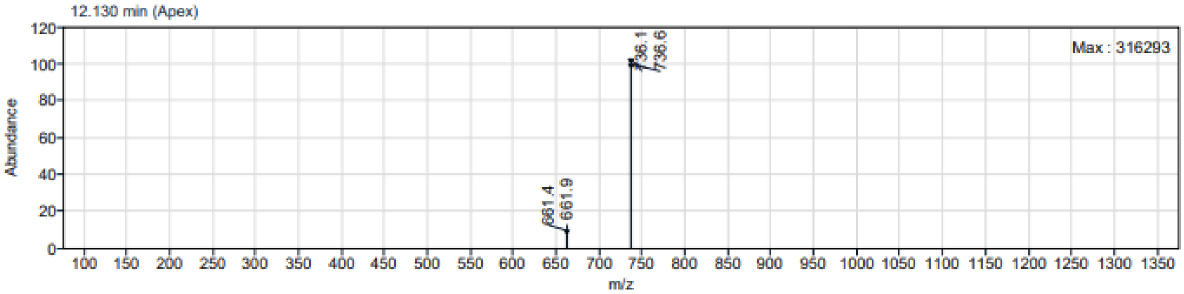

*Figure S9. HPLC and MS analysis of EBX **4f***

4. Peptide modification

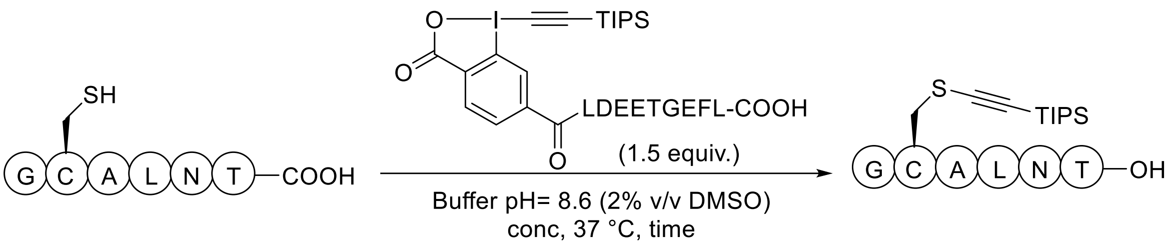

Table 1. Bioconjugation on peptide

Buffer	[Peptide] mM	Time	Conversion
**Tris 0.2 M**	3.5	15 min, RT	82
		1h, RT	80
**Tris 0.2 M**	1.7	15 min, RT	85
		1h, RT+ TCEP	85
**PB 0.2 M**	3.5	15 min 37 °C	83
		1 h 37 °C	81
**PBS**	3.5	15 min 37 °C	58
		1 h 37 °C	53
**HEPES 0.2 M**	3.5	15 min, 37 °C	65
		Then 1 h, 37 °C	65

5. Protein expression

Expression and purification:

Recombinant human Kelch domain of Keap1 (321–609) with an N-terminal addition of 6×His-tag and tobacco etch virus (TEV) cleavage site for tag removal was cloned into the vector pET29b(+) (Novagen) for bacterial expression. The DNA coding these elements was synthesized (GenScript) and inserted using the restriction enzymes NdeI and XhoI. The ligated vector was transformed into NEB® 5-alpha competent E. coli cells, and the cells plated on kanamycin-selective 2xYT agar plates. The DNA coding for the Keap1 protein was sequenced to verify the correctness of the sequence and the plasmid was transformed into BL21(DE3) competent E. coli cells (New England Biolabs) for expression. The plasmids encoding Keap1 mutants (Keap1-C434A) were prepared following the same procedure.

For expression, cells from colonies grown on 2xYT agar plates with 50 µg mL-1 of kanamycin were used to inoculate a 20 mL starter culture of 2xYT with 50 µg mL-1 kanamycin and grown over night at 37°C while being shaken. A volume of 800 mL 2xYT in a 2.5 L shaking flask was inoculated with the starter cultures by 1:100 dilution and grown at 37°C under shaking at 200 rpm until the logarithmic growth phase was reached. Protein expression was induced by adding a final concentration of 0.5 mM IPTG and the culture incubated at 20°C while being shaken overnight (200 rpm). Cells were pelleted by centrifugation at 4,000 g for 10 minutes. Cells were lysed by sonication in 80 mL lysis buffer (50 mM Tris, 500 mM NaCl, 20 mM imidazole, 10% ethylene glycol, pH 8.0). The cell lysate was clarified by centrifugation at 16,000 g for 60 min at 4 °C. The desired protein was purified from the culture supernatant by Ni-NTA affinity chromatography. Imidazole in the eluted protein was removed by PD-10 column (Cytiva) using Tris buffer (25 mM Tris, 20 mM NaCl, pH 8.0) and the protein purified by size exclusion chromatography (Cytiva Superdex 75 10/300 GL) using the same buffer. Purified protein was

concentrated using a spin filter (Microsep Advance Centrifugal Device with Omega Membrane 10k, Pall) and the concentration quantified by absorbance measurement at 280 nm using a NanoDrop 8000 Spectrophotometer. Concentrated protein was aliquoted and flash-frozen in liquid nitrogen and stored at -80°C for further use.

a

Protein sequence:

*GSSHHHHHH SSG*ENLYFQS

321
APKVGRLIYT AGGYFRQSLS YLEAYNPSDG TWLRLADLQV PRSGLAGCVV
GGLLYAVGGR NNSPDGNTDS SALDCYNPMT NQWSPCAPMS VPRNRIGVGV
IDGHIYAVGG SHGCIHHNSV ERYEPERDEW HLVAPMLTRR IGVGVAVLNR
LLYAVGGFDG TNRLNSAECY YPERNEWRMI TAMNTIRSGA GVCVLHNCIY
AAGGYDGQDQ LNSVERYDVE TETWTFVAPM KHRRSALGIT VHQGRIYVLG
GYDGHTFLDS VECYDPDTDT WSEVTRMTSG RSGVGVAVT 609

b

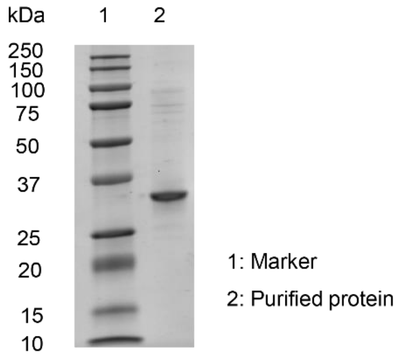

Figure S10. Keap 1 cloning and expression: a. Amino acid sequence of the Kelch domain of humain Keap 1 protein. Amino acids appended at the N-terminus containing His-tag are shown in italics, the TEV cleavage site is underlined and the Kelch domain of Keap 1 (amino acids 321-609) is in bold. B. SDS-PAGE analysis of the purified Keap1 protein

LC-MS and Gel

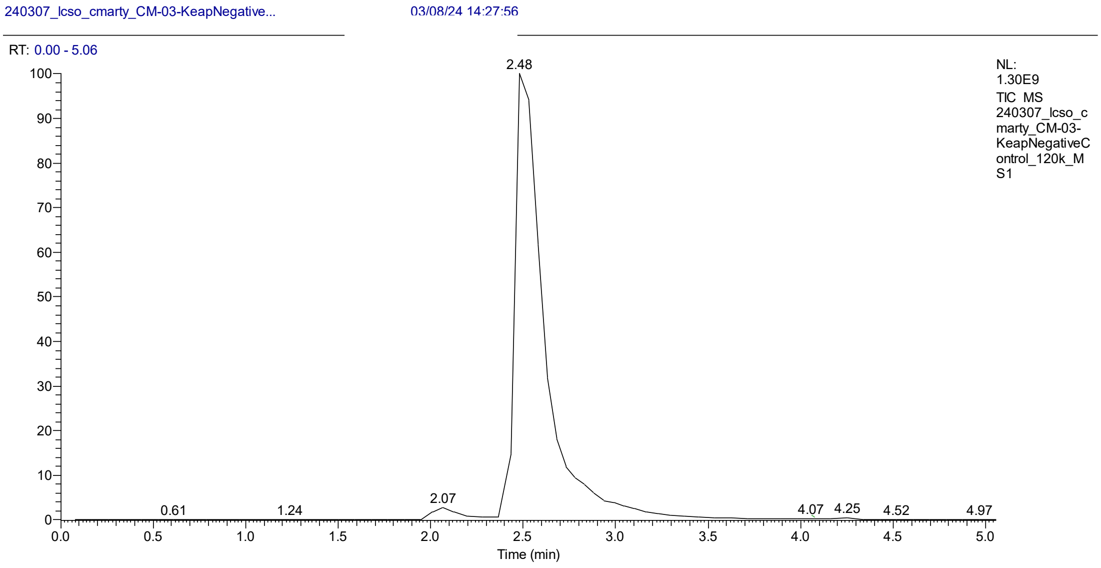
240307_lcso_cmarty_CM-03-KeapNegative...
03/08/24 14:27:56
RT: 0.00 - 5.06
NL: 1.30E9
TIC MS 240307_lcso_cmarty_CM-03-KeapNegativeControl_120k_MS1
2.48
2.07
0.61
1.24
4.07
4.25
4.52
4.97
Time (min)

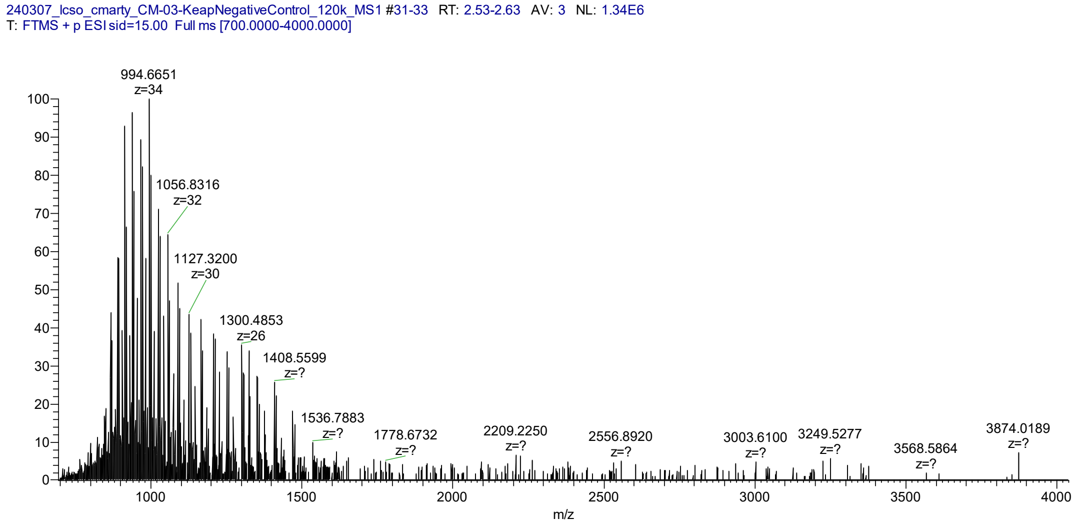
240307_lcso_cmarty_CM-03-KeapNegativeControl_120k_MS1 #31-33 RT: 2.53-2.63 AV: 3 NL: 1.34E6
T: FTMS + p ESI sid=15.00 Full ms [700.0000-4000.0000]
994.6651 z=34
1056.8316 z=32
1127.3200 z=30
1300.4853 z=26
1408.5599 z=?
1536.7883 z=?
1778.6732 z=?
2209.2250 z=?
2556.8920 z=?
3003.6100 z=?
3249.5277 z=?
3568.5864 z=?
3874.0189 z=?
m/z

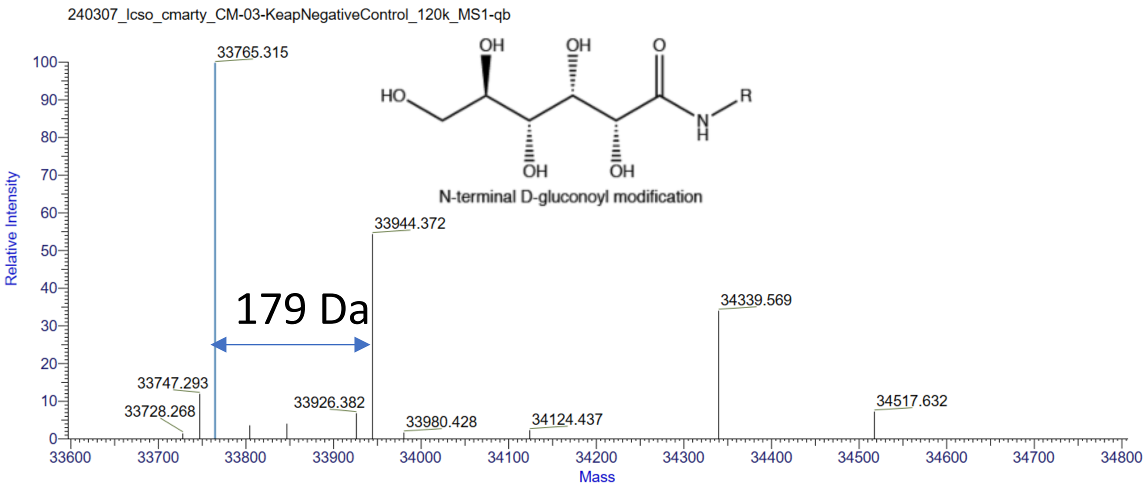
240307_lcso_cmarty_CM-03-KeapNegativeControl_120k_MS1-qb
Relative Intensity
33765.315
33944.372
34339.569
33747.293
33728.268
33926.382
33980.428
34124.437
34517.632
179 Da
N-terminal D-gluconoyl modification
Mass

Figure S11. Combined chromatogram, ions series and deconvoluted mass spectra of KEAP WT. The second peak correspond to the modification of the N terminus occurring during the expression. (Monoisotopic mass)[9]

Note: It was showed in ref 7 that several proteins expressed in Escherichia coli with the *N*-terminus Gly-Ser-Ser-[His]$_6$- consisted partly of material with 178 Da of excess mass corresponding to synthetic α-N-d-gluconoyl-His tag according to NMR spectra. A plausible mechanism described by the authors was acylation by 6-phosphoglucono-1,5-lactone, produced from glucose 6-phosphate by glucose-6-phosphate dehydrogenase (EC 1.1.1.49).

[9] Geoghegan, K.F.; Dixon, H.B.; Rosner, P.J., Hoth, L.R.; Lanzetti, A.J.; Borzilleri, K.A., Marr, E.S.; Pezzullo, L.H.; Martin, L.B.; LeMotte, P.K.; McColl, A.S. Spontaneous α-*N*-6-Phosphogluconoylation of a “His Tag” in *Escherichia coli:* The Cause of Extra Mass of 258 or 178 Da in Fusion Proteins, *Anal. Biochem.* **1999**, *267*, 169-184.

Similar method was used to expressed the C434A mutant

a

Protein sequence:

GSSHHHHHH SSG<u>ENLYFQS</u>

321
APKVGRLIYT AGGYFRQSLS YLEAYNPSDG TWLRLADLQV PRSGLAGCVV
GGLLYAVGGR NNSPDGNTDS SALDCYNPMT NQWSPCAPMS VPRNRIGVGV
IDGHIYAVGG SHGAIHHNSV ERYEPERDEW HLVAPMLTRR IGVGVAVLNR
LLYAVGGFDG TNRLNSAECY YPERNEWRMI TAMNTIRSGA GVCVLHNCIY
AAGGYDGQDQ LNSVERYDVE TETWTFVAPM KHRRSALGIT VHQGRIYVLG
GYDGHTFLDS VECYDPDTDT WSEVTRMTSG RSGVGVAVT 609

b

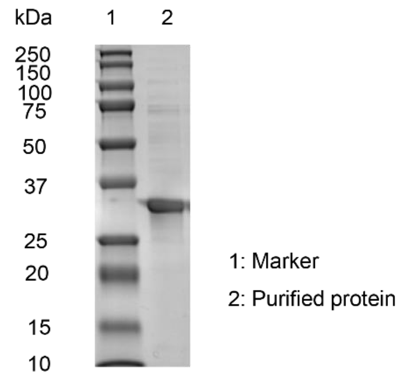

Figure S12. Keap 1 cloning and expression: a. Amino acid sequence of the Kelch domain of humain Keap 1 protein with C434A mutation. Amino acids appended at the N-terminus containing His-tag are shown in italics, the TEV cleavage site is underlined and the Kelch domain of Keap 1 (amino acids 321-609) is in bold. The mutation site is highlighted in red. B. SDS-PAGE analysis of the purified Keap1 protein

LC-MS and Gel

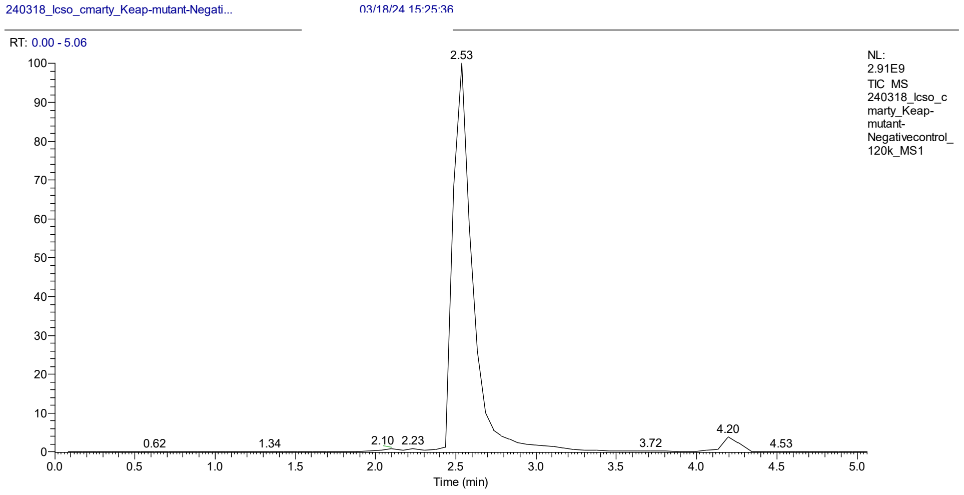

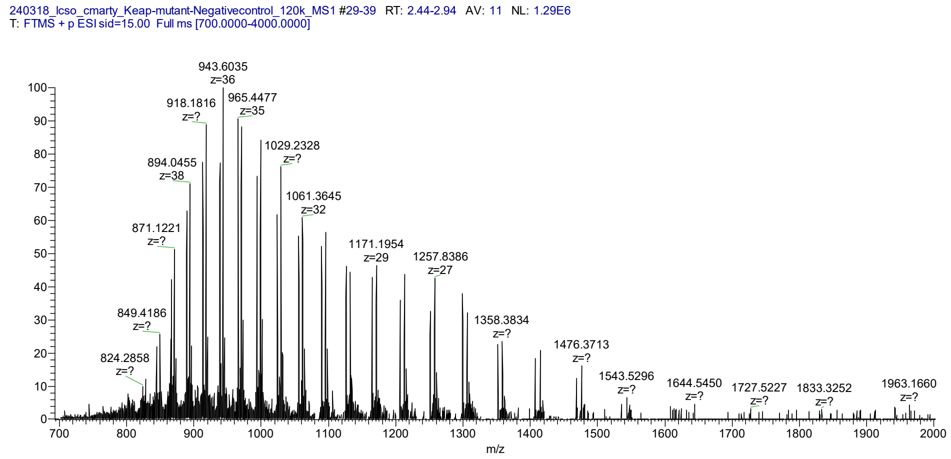

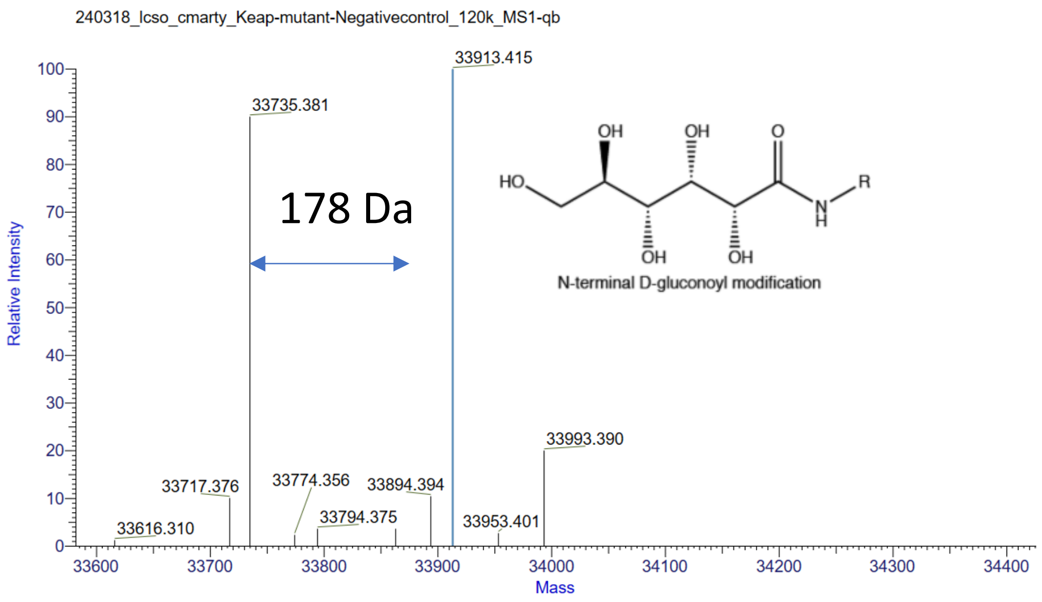

Figure S13. Combined chromatogram, ions series and deconvoluted mass spectra of KEAP C434A. The second peak correspond to the modification of the N terminus occurring during the expression. (Monoisotopic mass)

6. Protein bioconjugation

General procedure for Cys-selective bioconjugation to proteins:

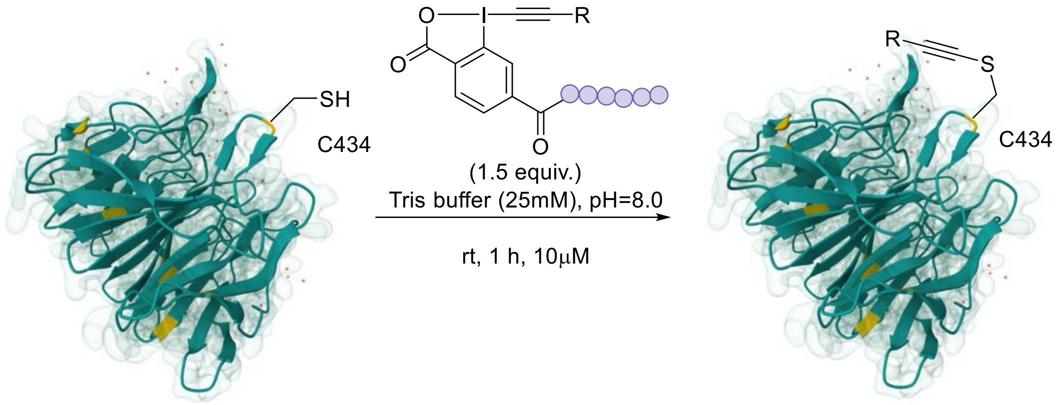

In a 1.5 mL Eppendorf Safe-Lock microcentrifuge, KEAP (32 μM in PBS stock solution, 25 μL) was diluted in Tris buffer (25 mM, pH 8, 55 μL). Then a solution of EBX (1 mM) in Tris buffer (25 mM, pH 8, 1.2 μL) was added in one portion. The resulting solution was vortexed few seconds to ensure proper reagent mixing and incubated at RT for 1 h. No effort was made to exclude oxygen.

Procedure for LC-MS/MS analysis:

Intact protein mass spectrometry analysis:

After reaction, samples were diluted to a final protein concentration of 5 μM with milliQ water containing 0.1% formic acid and were injected into an Acquity UPLC Protein column BEH C4 VanGuard (300 Å, 1.7 μm, 2.1 x 5 mm, Waters, Milford, MA, U.S.A.) using a Vanquish analytical LC system (Thermo Fisher Scientific, Germany) coupled to an OptaMax NG ion source (Thermo Fisher Scientific, Bremen, Germany). The sample desalting was performed with a flow rate of 400 μl/min by applying a gradient of solvent B from 15 to 60 % in 2.5 min, followed by column washing and re-equilibration steps. Solvent A was composed of milliQ water with 0.1 % formic acid, while solvent B consisted of acetonitrile with 0.1 % formic acid. Eluting proteoforms were analyzed on an Exploris 240 Orbitrap-FT-MS benchtop instrument (Thermo Fisher Scientific, Bremen, Germany) using Intact Protein mode with low pressure settings, positive polarity, standard AGC, maximum injection time (IT) set to auto, 120'000 resolution at 200 m/z and averaging 10 microscans. Intact mass measurement data were analyzed with BioPharma Finder 4.1 software (Thermo Fisher Scientific, Sunnyvale, CA, U.S.A.) using a Xtract algorithm with 90% fit factor.

The conversion was calculated by HRMS based on the following equation:

Conversion = [intensity of (Product peak) + intensity (Product peak + 178 Da)]/Sum intensity(All peaks).

a. Influence of the ligand

KEAP-EBX **4aa**

a. With KEAP WT, Full conversion

Ion series:

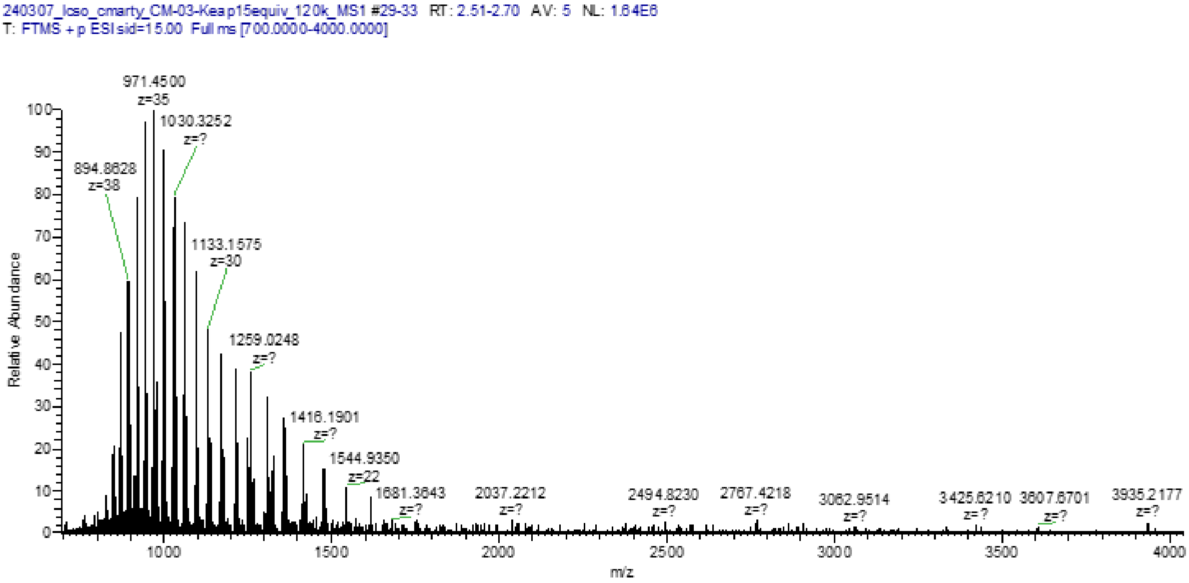

Deconvoluted mass spectrum:

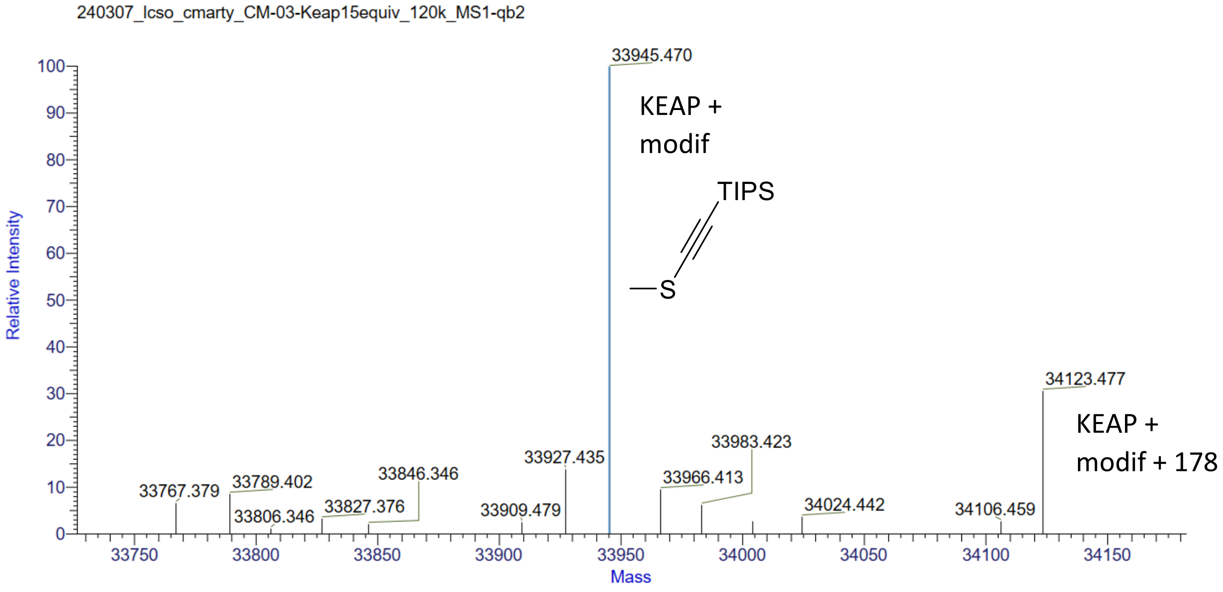

*Figure S14. Combined ions series and deconvoluted mass spectra of the modification with EBX **4aa** on KEAP WT. Calcd. Mass, 33946 Da, found: 33945 Da. Conversion 65%. (Monoisotopic mass).*

b. With KEAP C434A, No conversion

Ion series:

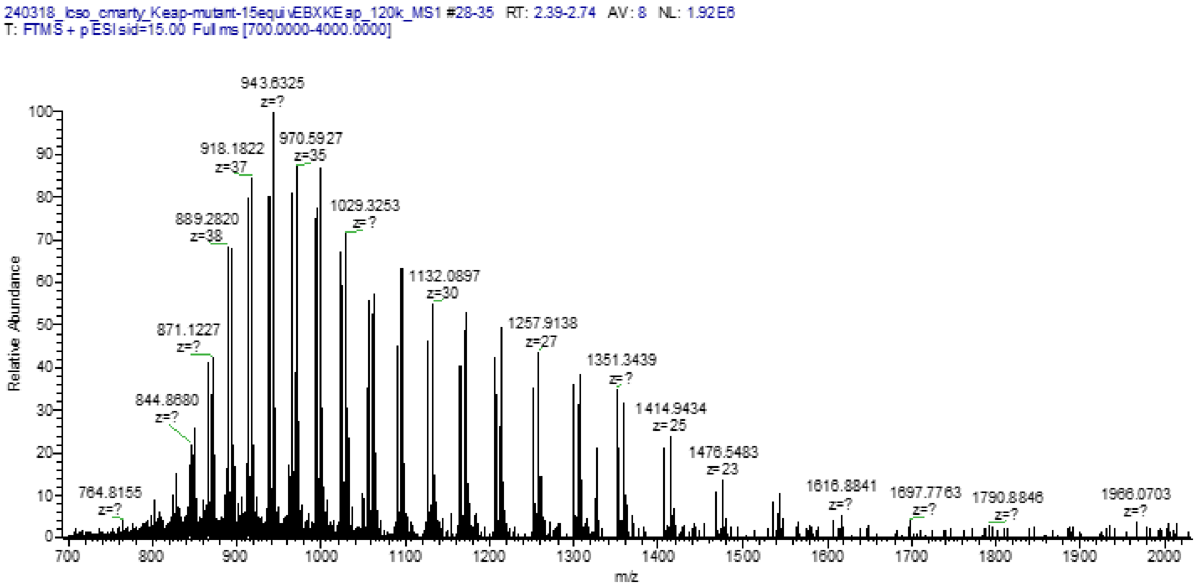

Deconvoluted mass spectrum:

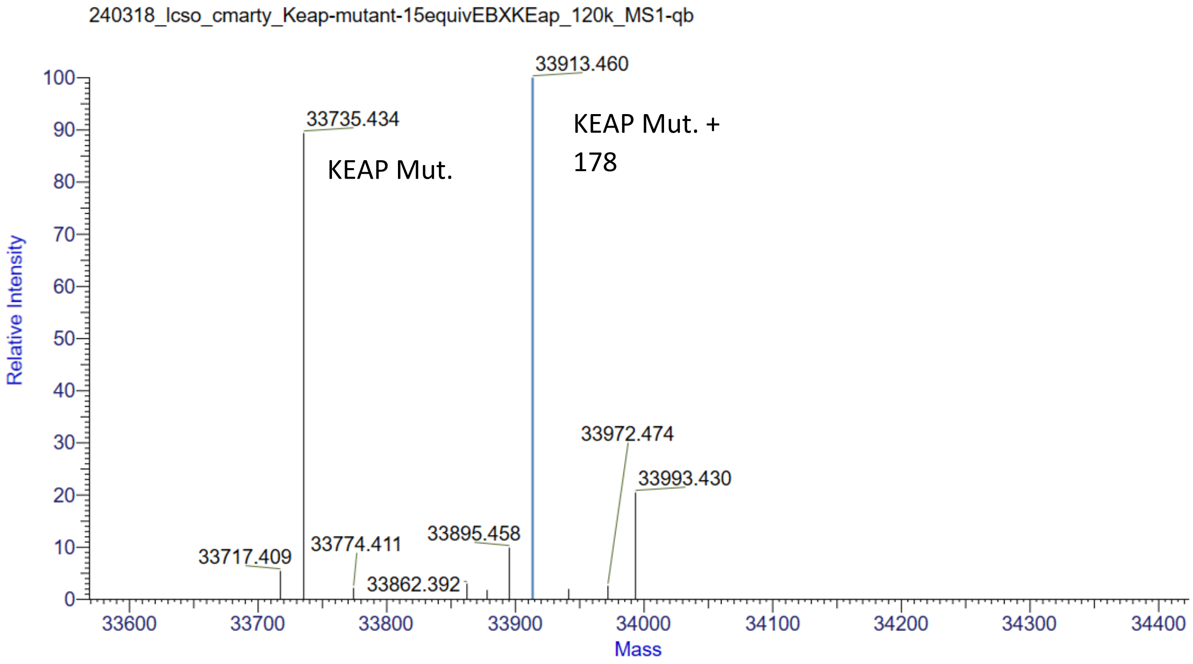

*Figure S15. Combined ions series and deconvoluted mass spectra of the modification with EBX **4aa** on KEAPC434A. No modification. (Monoisotopic mass)*

KEAP-EBX **4ab**

a. With KEAP WT, No conversion

Ions series:

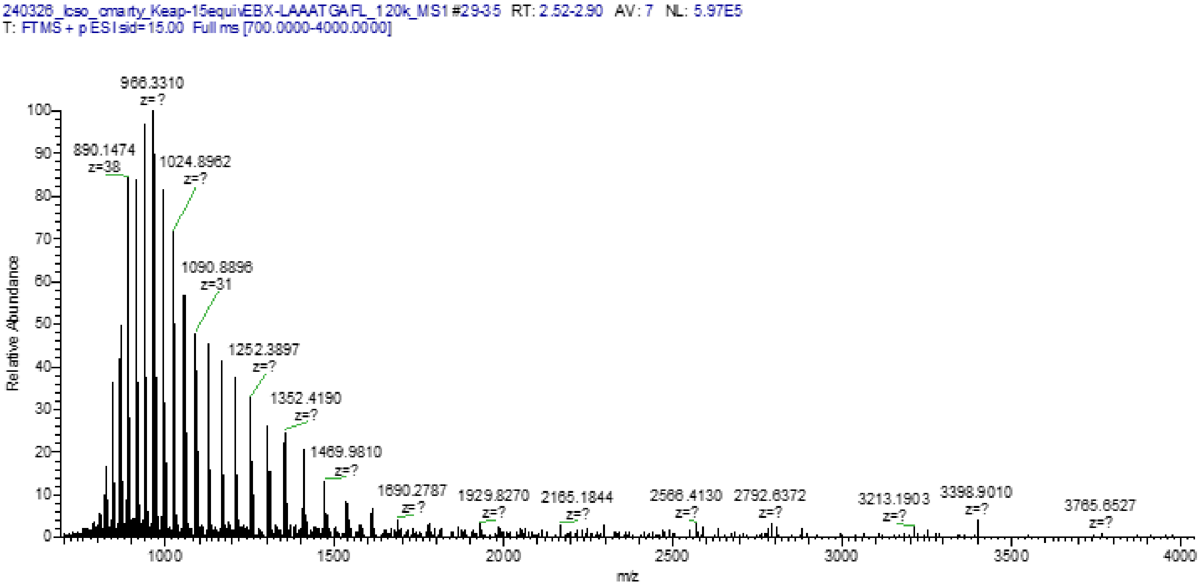

Deconvoluted mass spectrum:

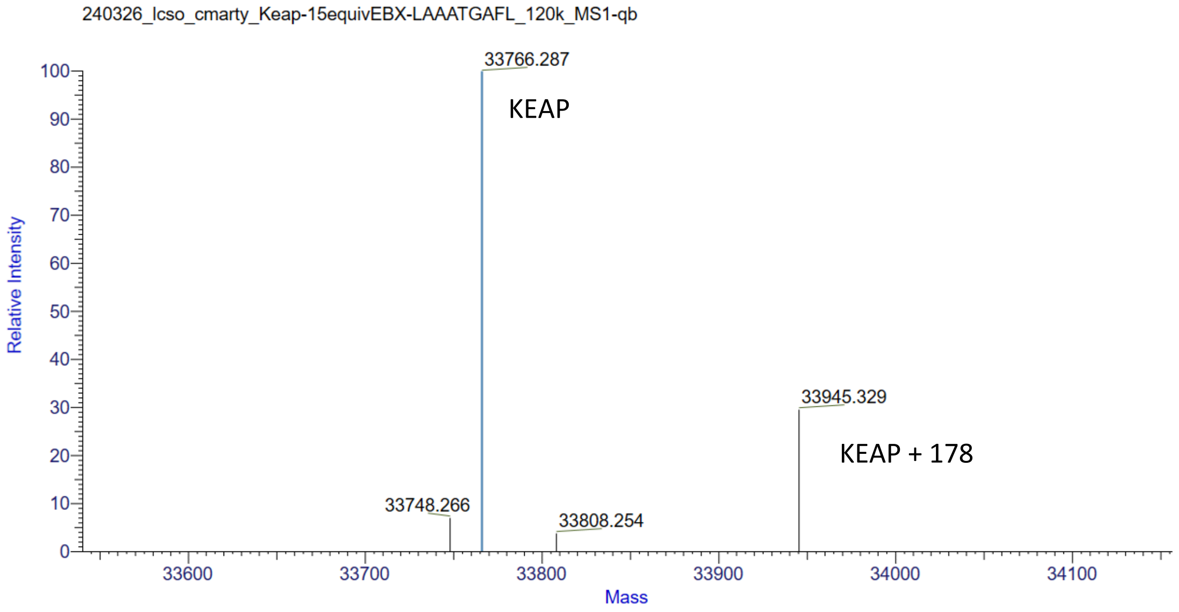

*Figure S16. Combined ions series and deconvoluted mass spectra of the modification with EBX **4ab** on KEAP WT. No modification. (Monoisotopic mass)*

KEAP-EBX **4ac**

a. With KEAP WT, No conversion

Ions series:

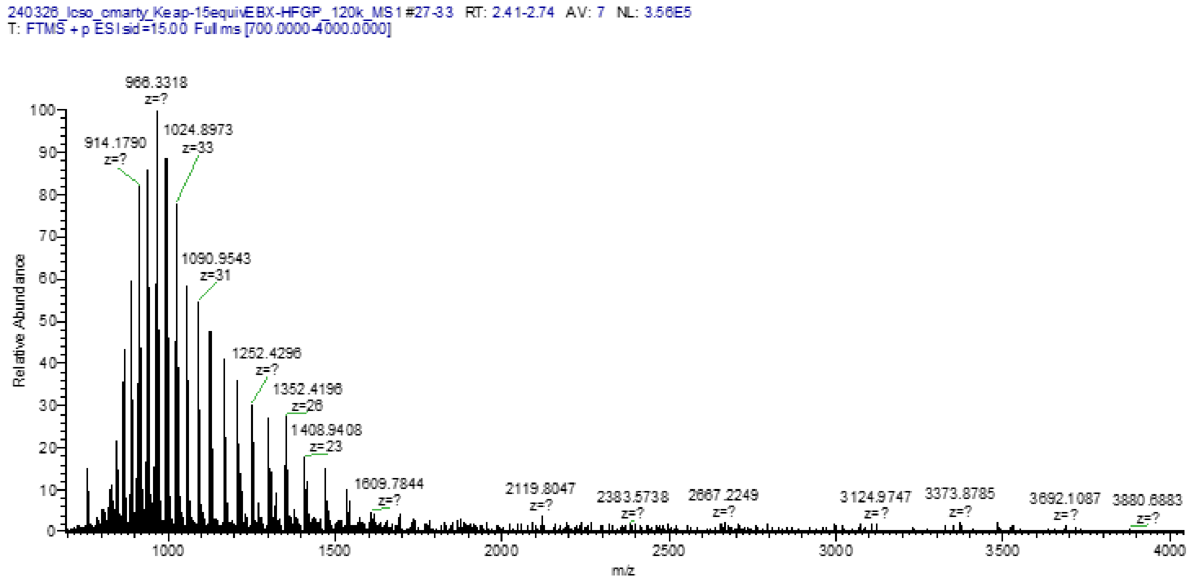

Deconvoluted mass spectrum:

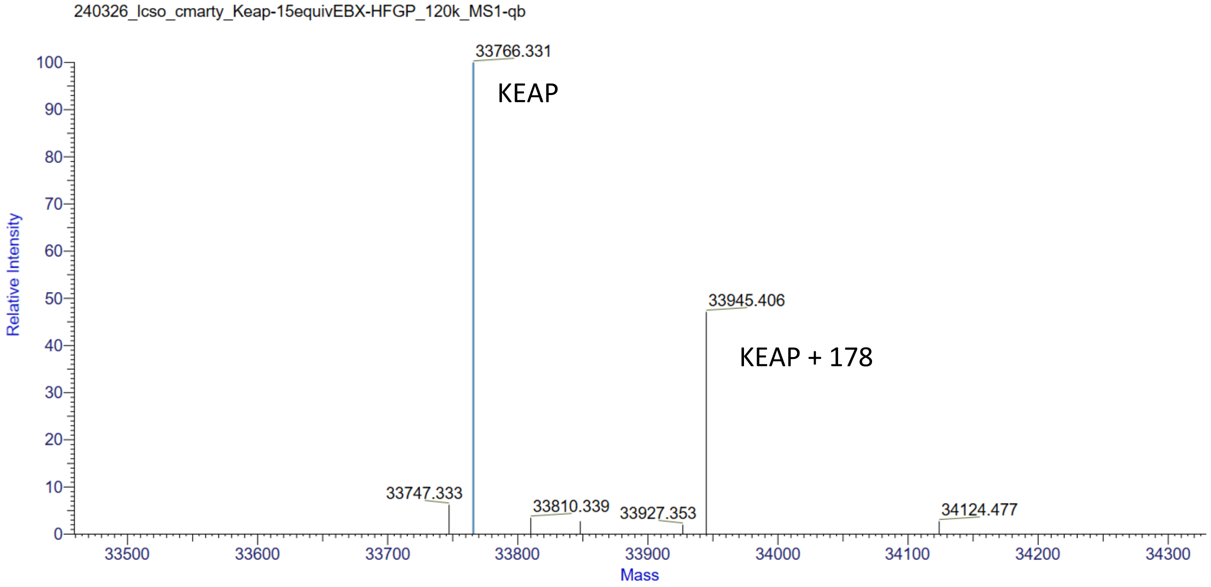

*Figure S17. Combined ions series and deconvoluted mass spectra of the modification with EBX **4ac** on KEAP WT. Conversion <10%. (Monoisotopic mass)*

b. With KEAP C434A, No conversion

Ions series:

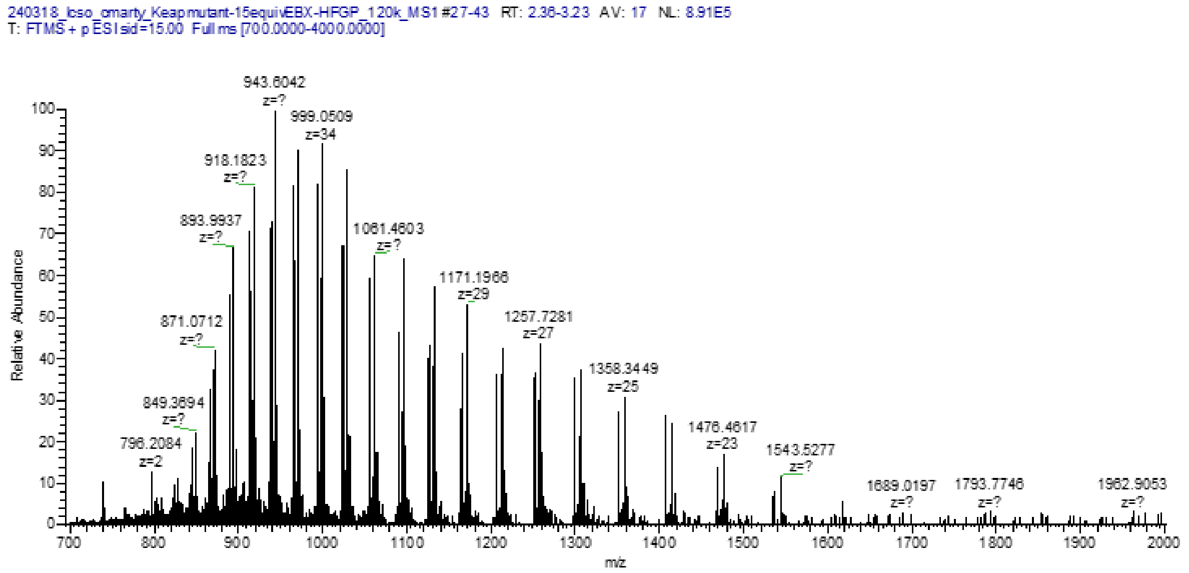

Deconvoluted mass spectrum:

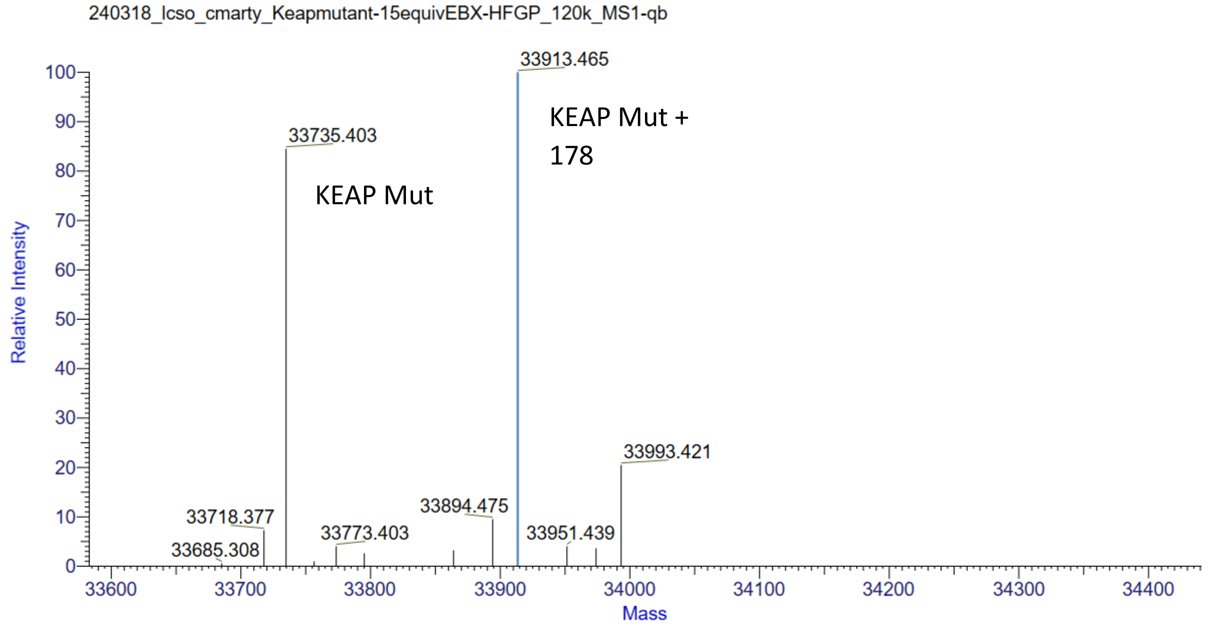

*Figure S18. Combined ions series and deconvoluted mass spectra of the modification with EBX **4ac** on KEAP C434A. No modification. (Monoisotopic mass)*

KEAP-EBX **10**

a. With KEAP WT,

Ions series:

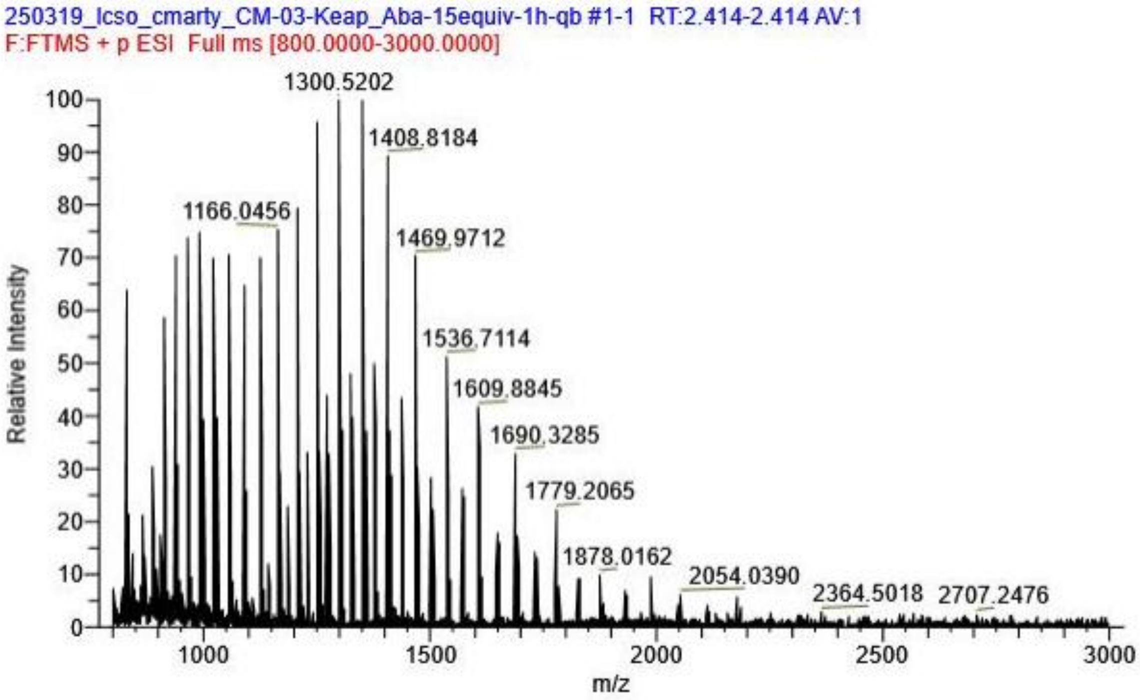

Deconvoluted mass spectrum:

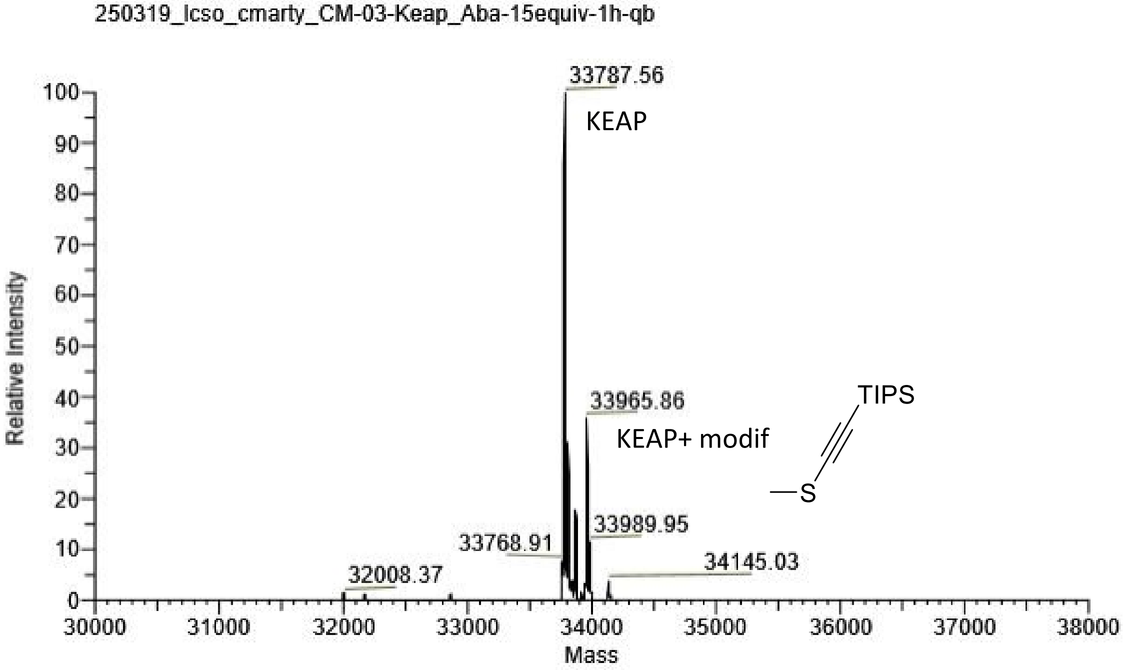

*Figure S19. Combined ions series and deconvoluted mass spectra of the modification with **10** on KEAP WT. Conversion <10%. (Average mass)*

b. With KEAP C434A, No conversion

Ions series:

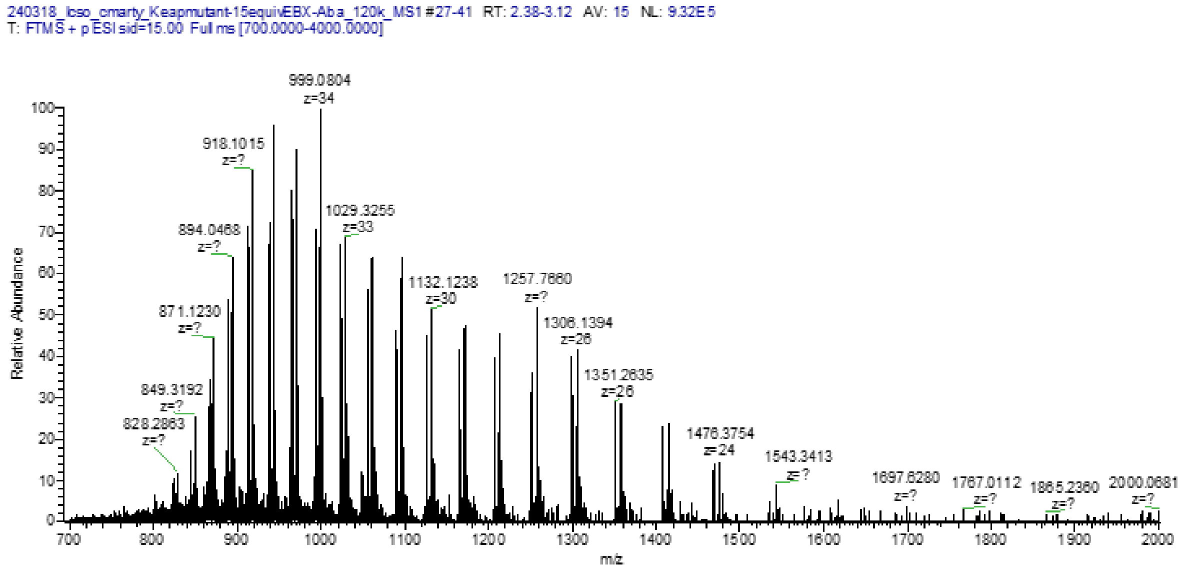

Deconvoluted mass spectrum:

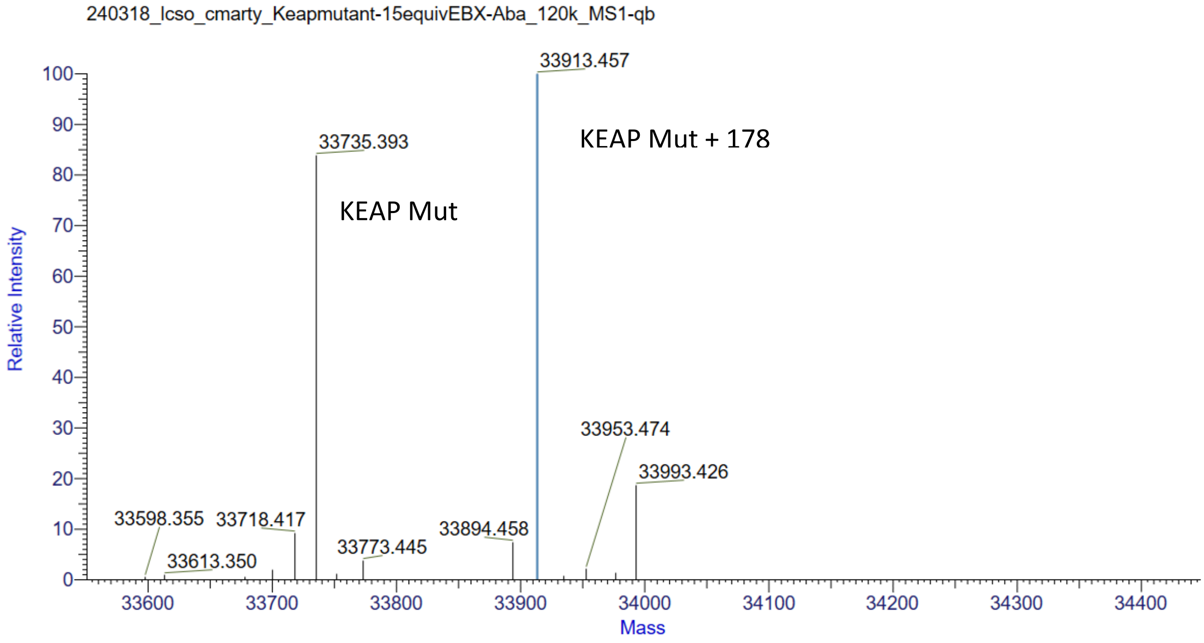

*Figure S20. Combined ions series and deconvoluted mass spectra of the modification with **10** on KEAP C434A. No modification. (Monoisotopic mass)*

Azido-EBX **2**

a. With KEAP WT, (15 equiv. of Azido-EBX **2**)

Ions series:

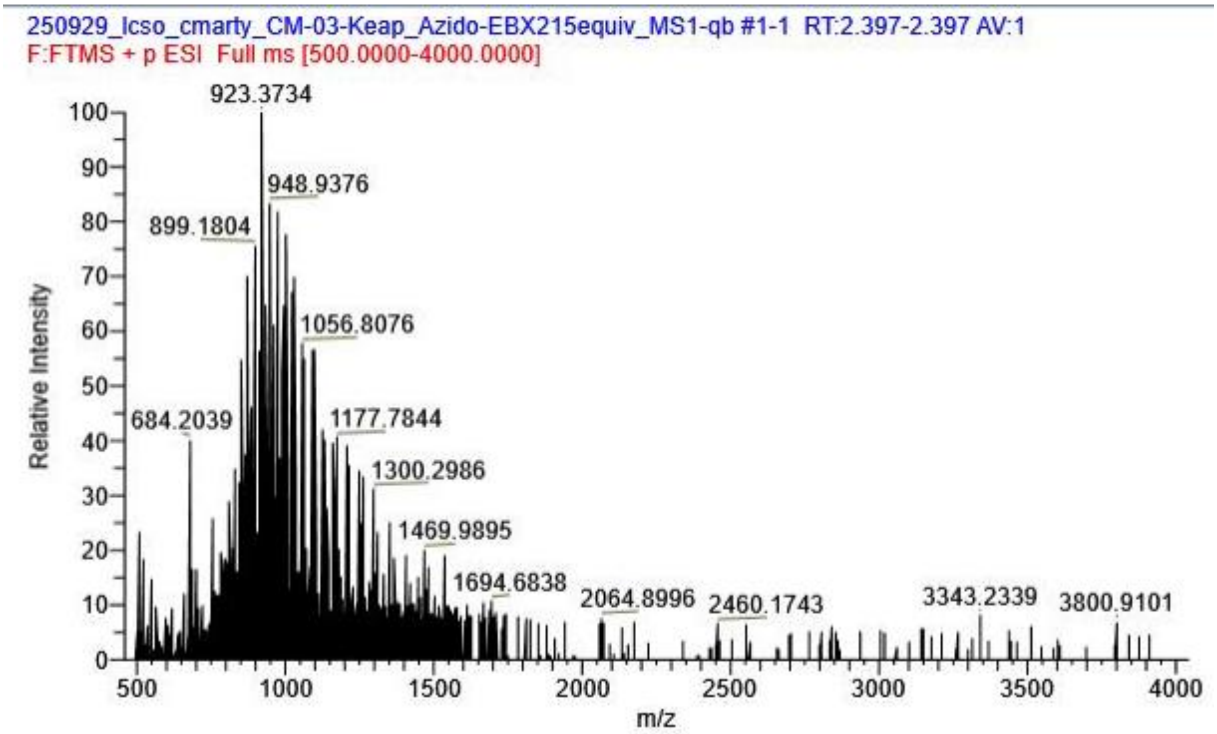

Deconvoluted mass spectrum:

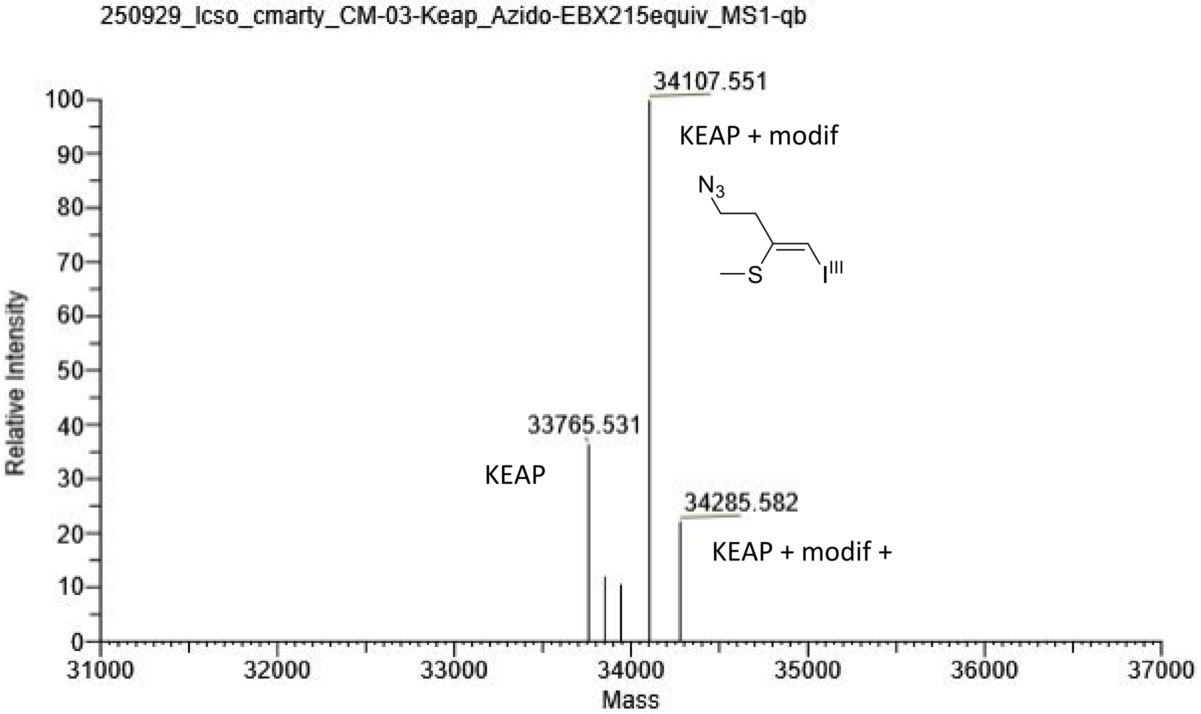

*Figure S21. Combined ions series and deconvoluted mass spectra of the modification with **2** on KEAP WT. Calcd. Mass, 34106 Da, found: 34107 Da. Conversion 67%. (Monoisotopic mass)*

b. With KEAP C434A, (15 equiv. of Azido-EBX **2**)

Ions series:

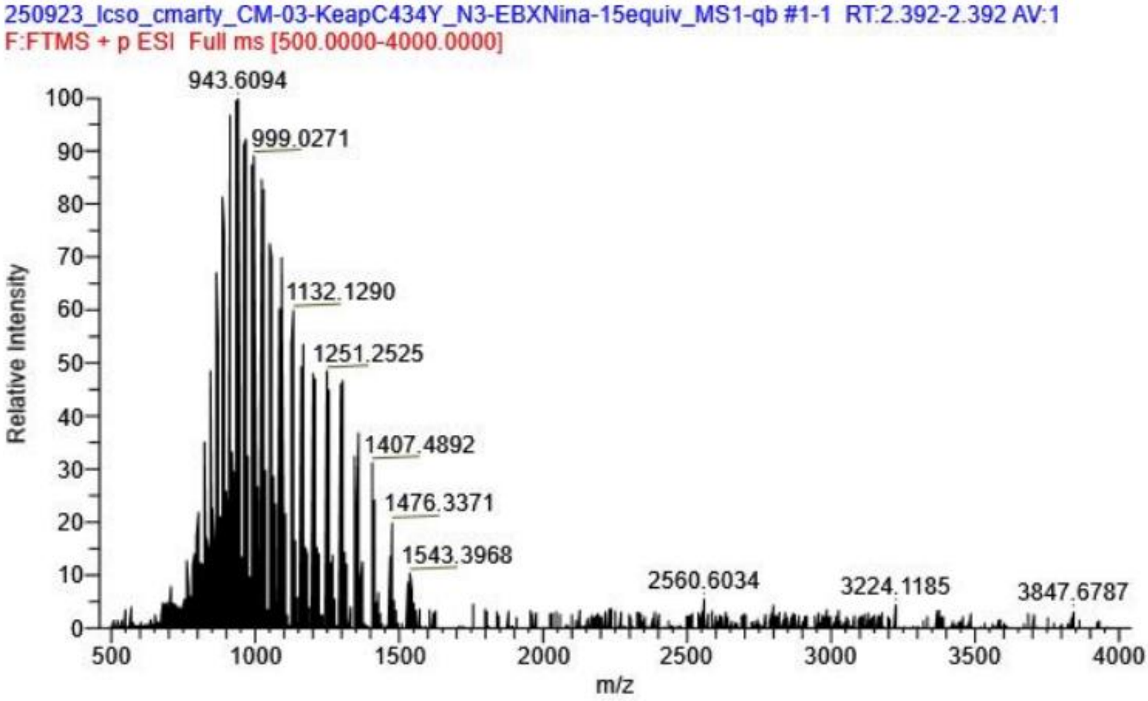

Deconvoluted mass spectrum:

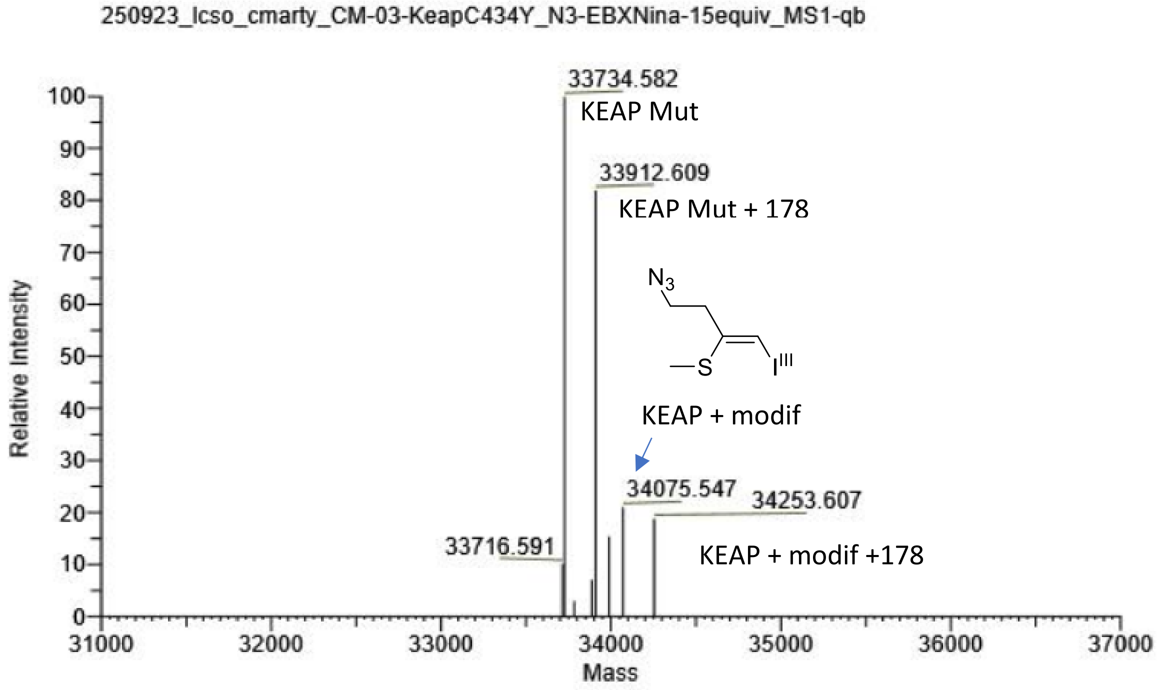

*Figure S22. Combined ions series and deconvoluted mass spectra of the modification with **2** on KEAP C434Y. Calcd. Mass, 34075 Da, found: 34075 Da. Conversion 15%. (Monoisotopic mass)*

b. Scope of EBX

KEAP-EBX **4b**

Ions series:

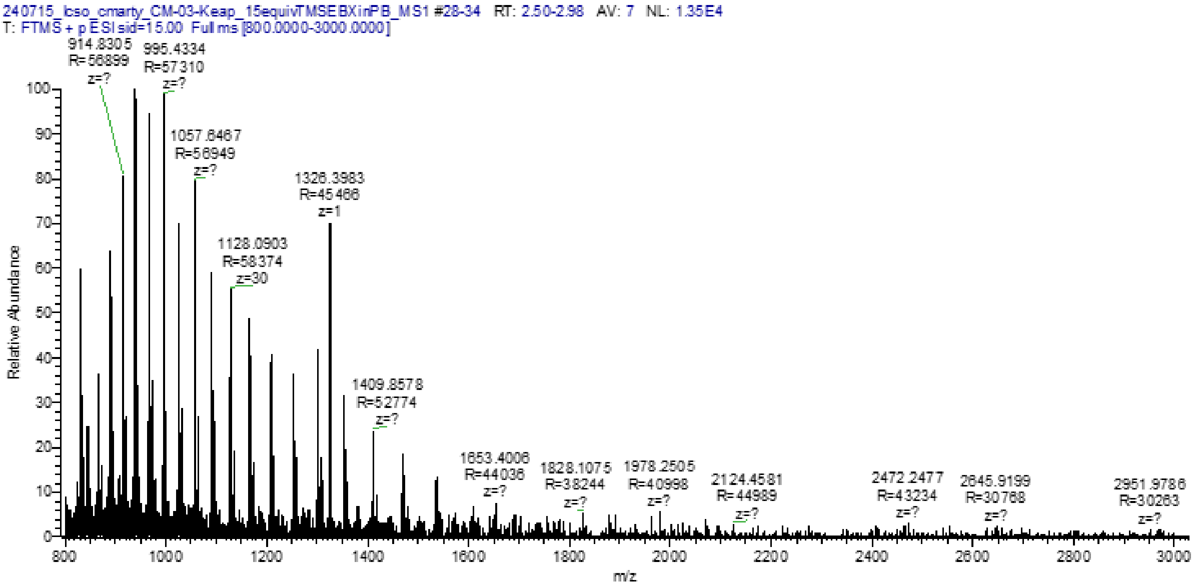

Deconvoluted mass spectrum:

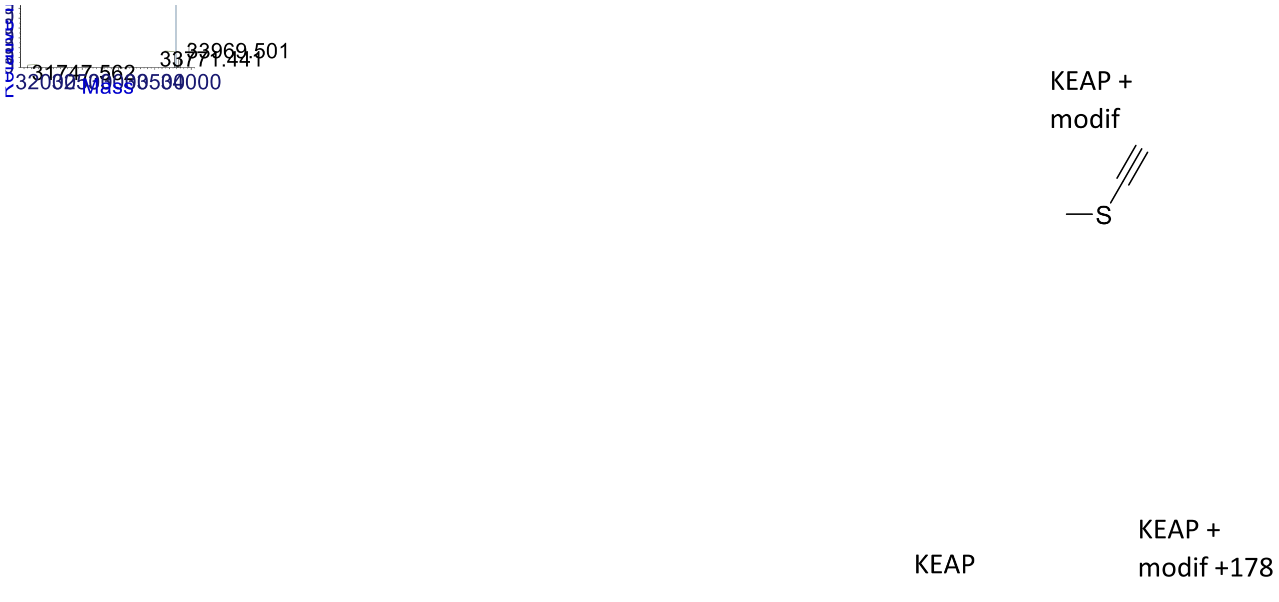

*Figure S23. Combined ions series and deconvoluted mass spectra of the modification with EBX **4b** on KEAP WT. Calcd. Mass, 33795 Da, found: 33791 Da. Conversion 77%. (Monoisotopic mass)*

KEAP-EBX **4ca**

Ions series:

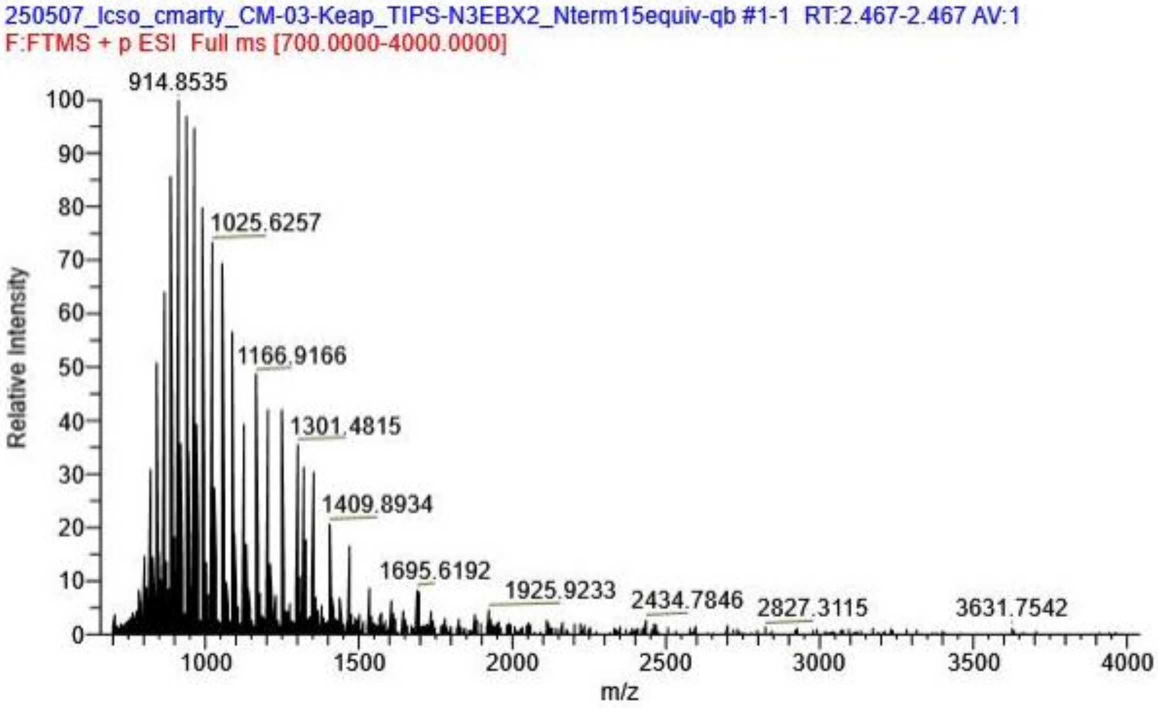

Deconvoluted mass spectrum:

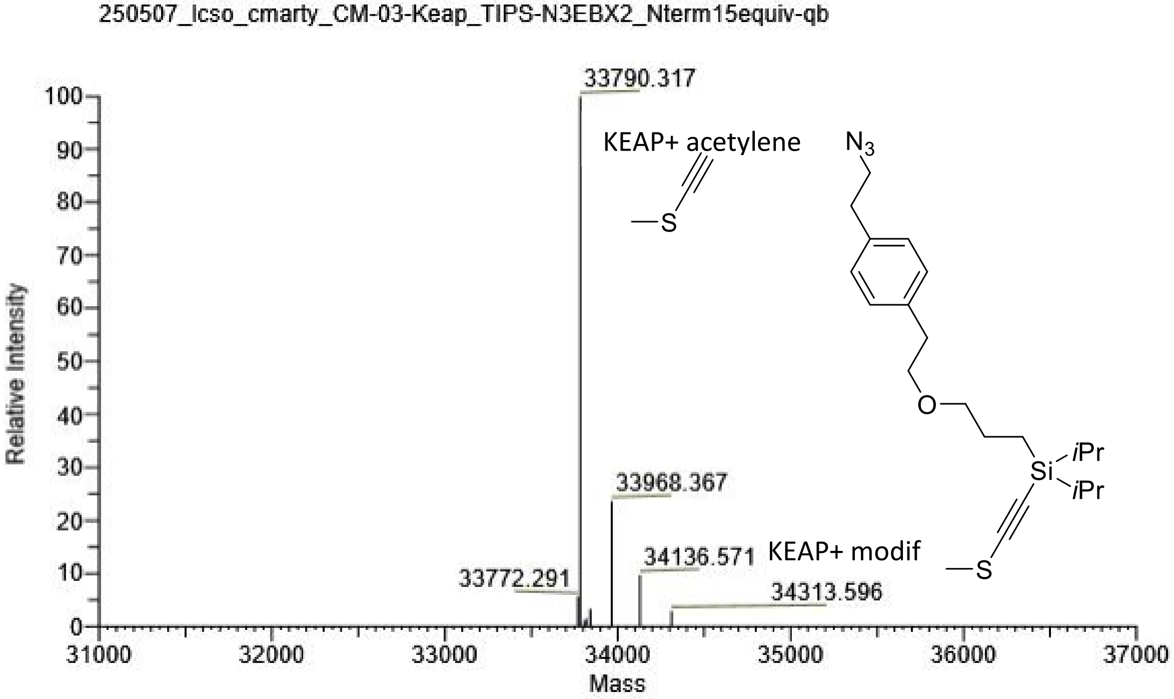

*Figure S24. Combined ions series and deconvoluted mass spectra of the modification with EBX **4ca** on KEAP WT. Calcd. Mass, 34136 Da, found: 34135 Da. Conversion 10% + by-product 83% conversion (Monoisotopic mass)*

KEAP-EBX **4cb**

Ions series:

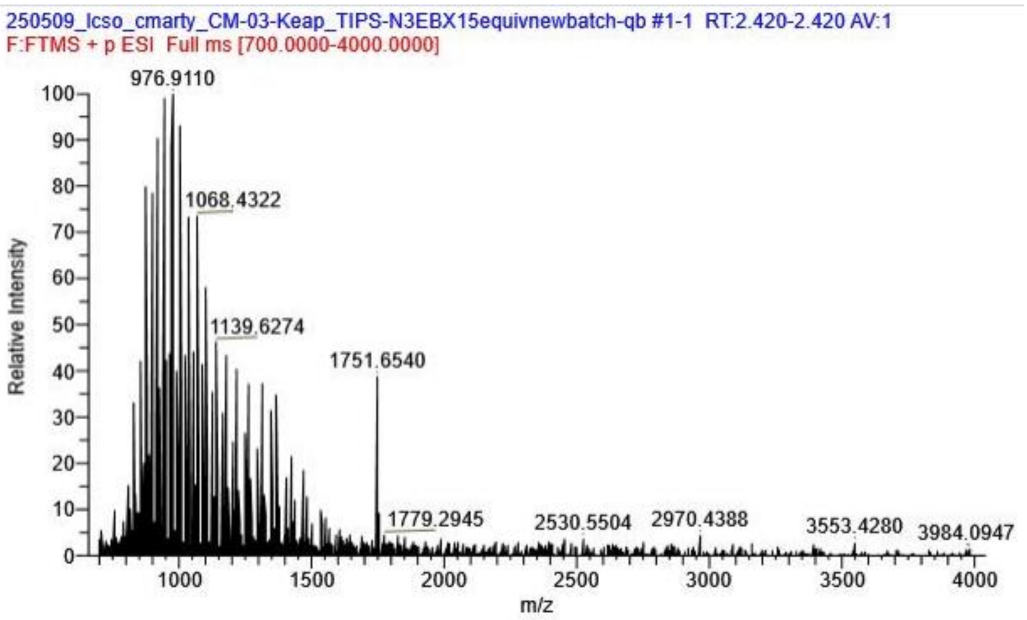

Deconvoluted mass spectrum:

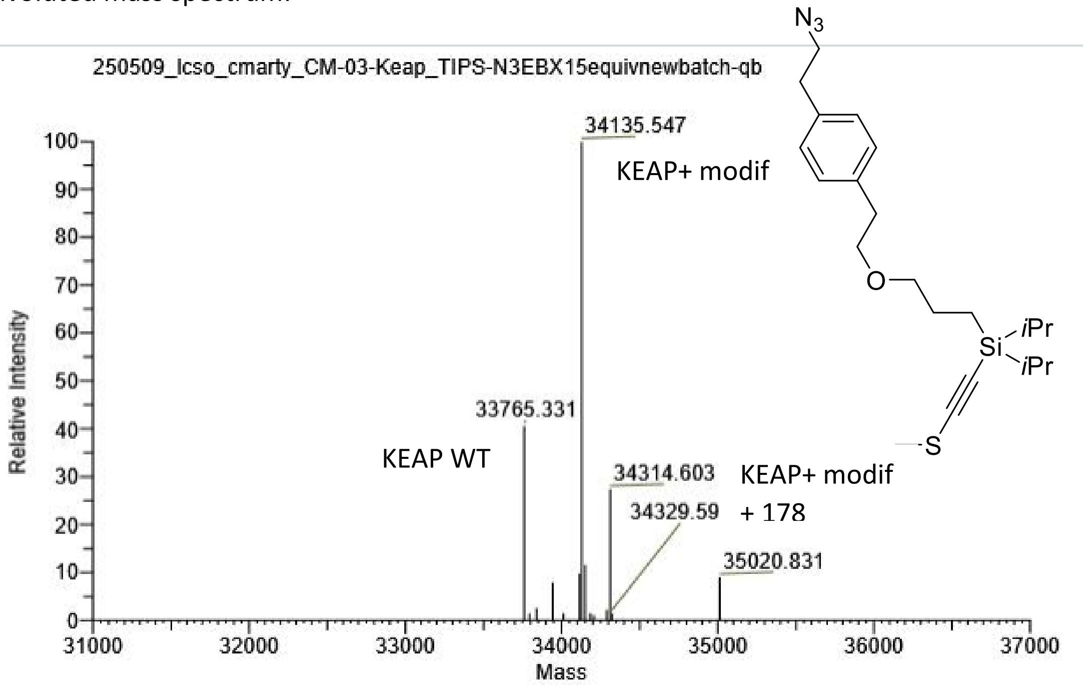

*Figure S25. Combined ions series and deconvoluted mass spectra of the modification with EBX **4cb** on KEAP WT. Calcd. Mass, 34136 Da, found: 34135 Da. Conversion 57%. (Monoisotopic mass)*

KEAP-EBX **4d**

Ions series:

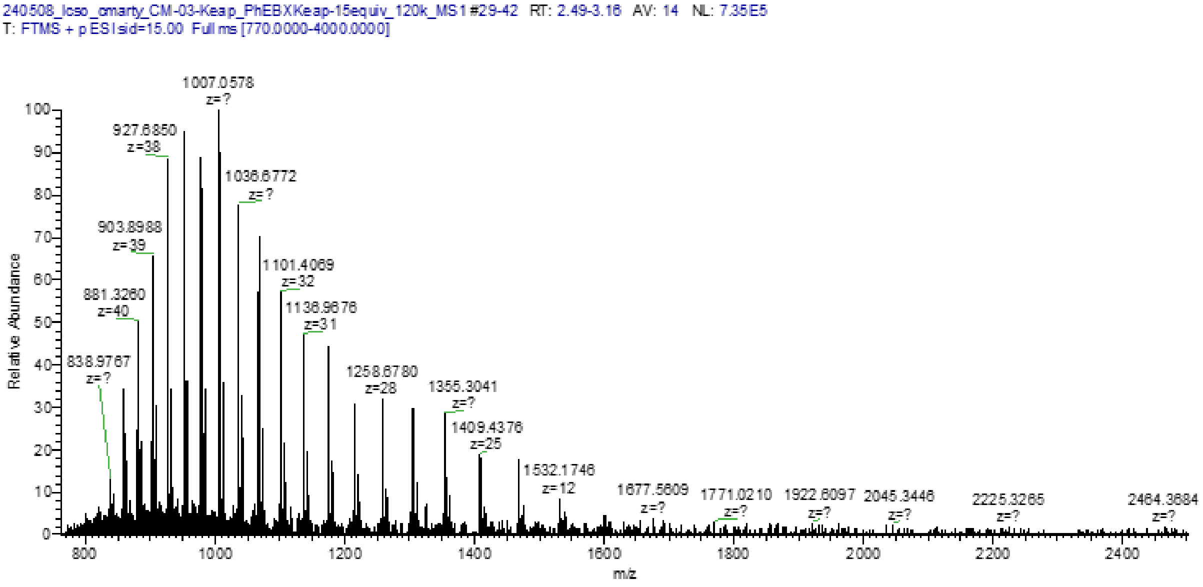

Deconvoluted mass spectrum:

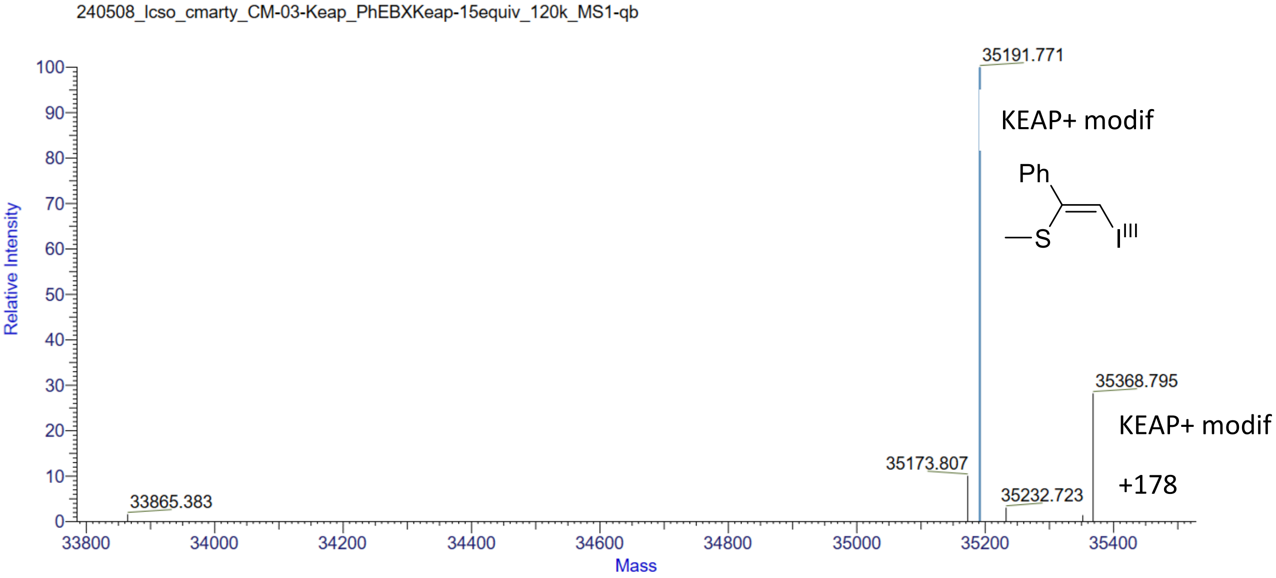

*Figure S26. Combined ions series and deconvoluted mass spectra of the modification with EBX **4d** on KEAP WT. Calcd. Mass, 35191 Da, found: 35192 Da. Conversion 88%. (Monoisotopic mass)*

KEAP-EBX **4e**

Ions series:

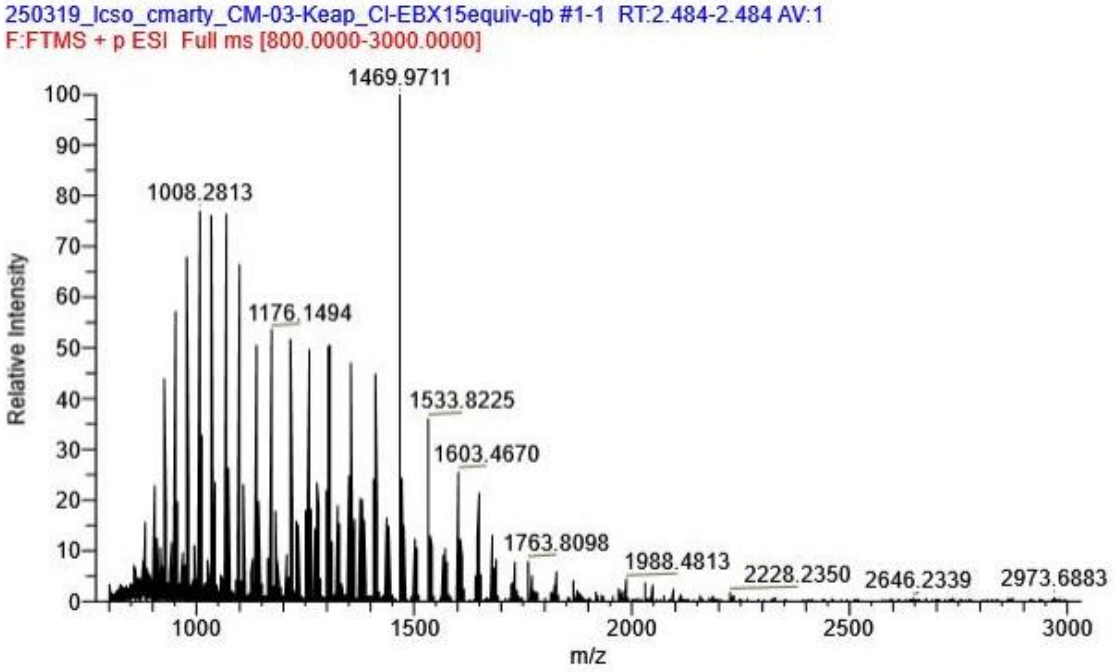

Deconvoluted mass spectrum:

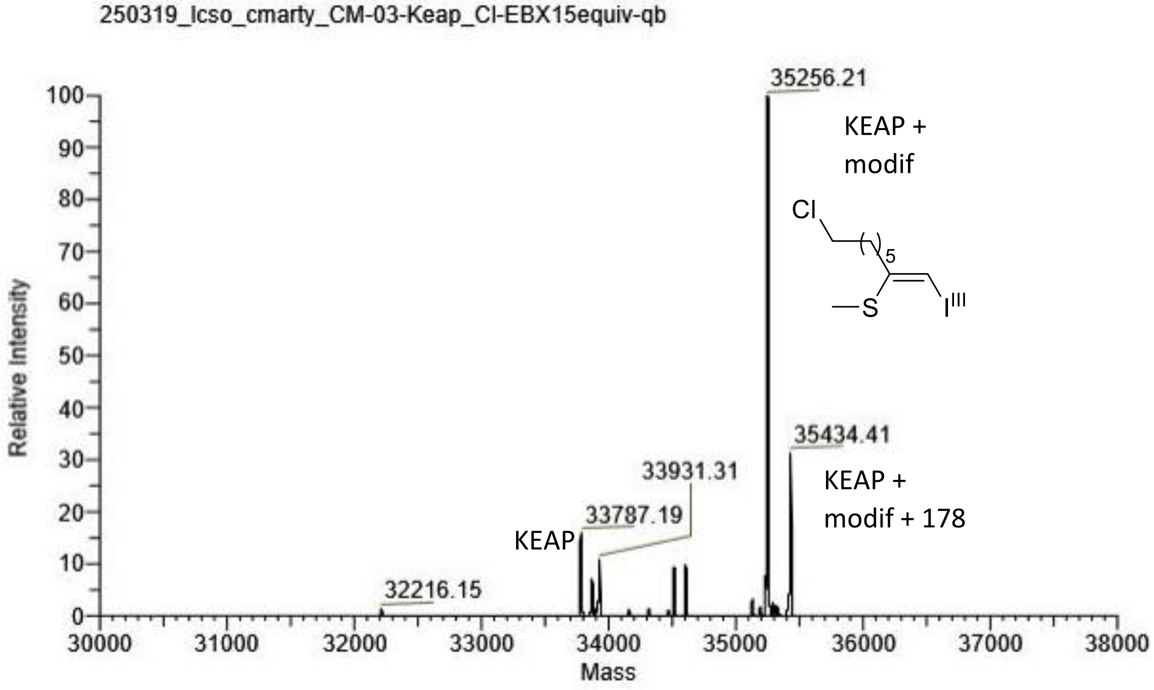

Figure S27. Combined ions series and deconvoluted mass spectra of the modification with EBX ***4e*** *on KEAP WT. Calcd. Mass, 35254 Da, found: 35256 Da. Conversion 61%. (Average mass)*

KEAP-EBX **4f**

Ions series:

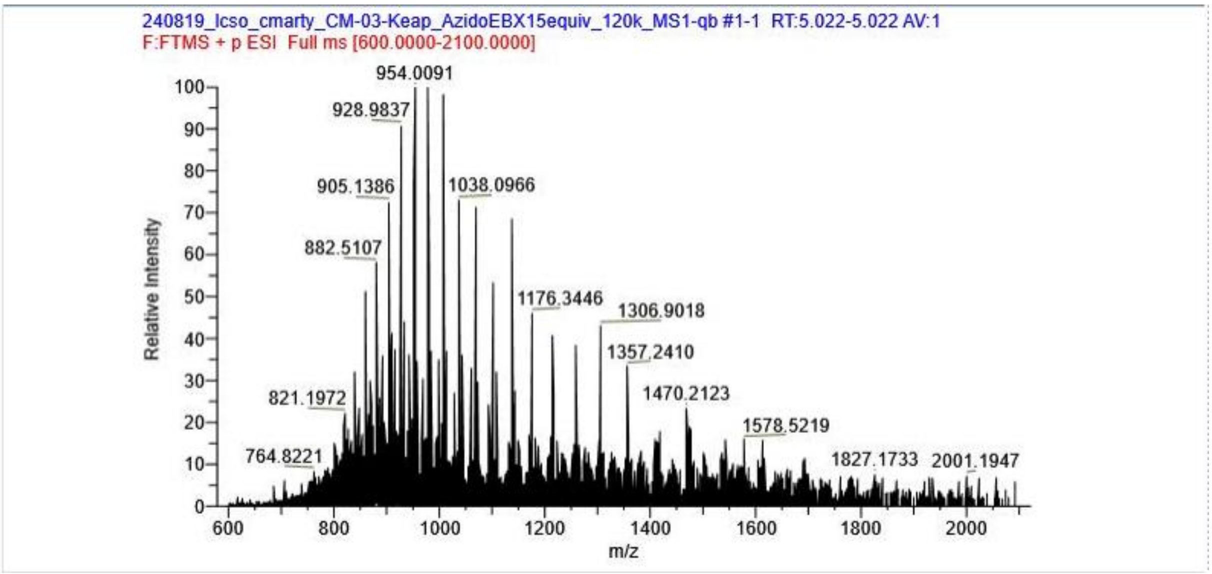

Deconvoluted mass spectrum:

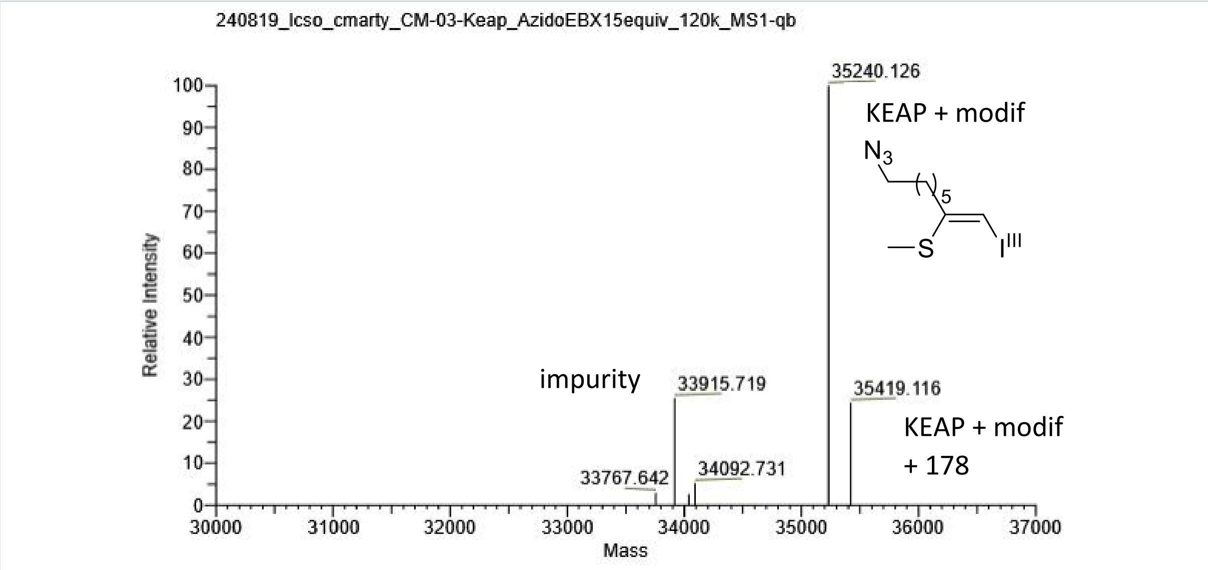

Figure S28. Combined ions series and deconvoluted mass spectra of the modification with EBX ***4f*** *on KEAP WT. Calcd. Mass, 35240 Da, found: 35240 Da. Conversion 77%. (Monoisotopic mass)*

7. Protein Post Modification

a. SPAAC

Synthesis of BCN reagent

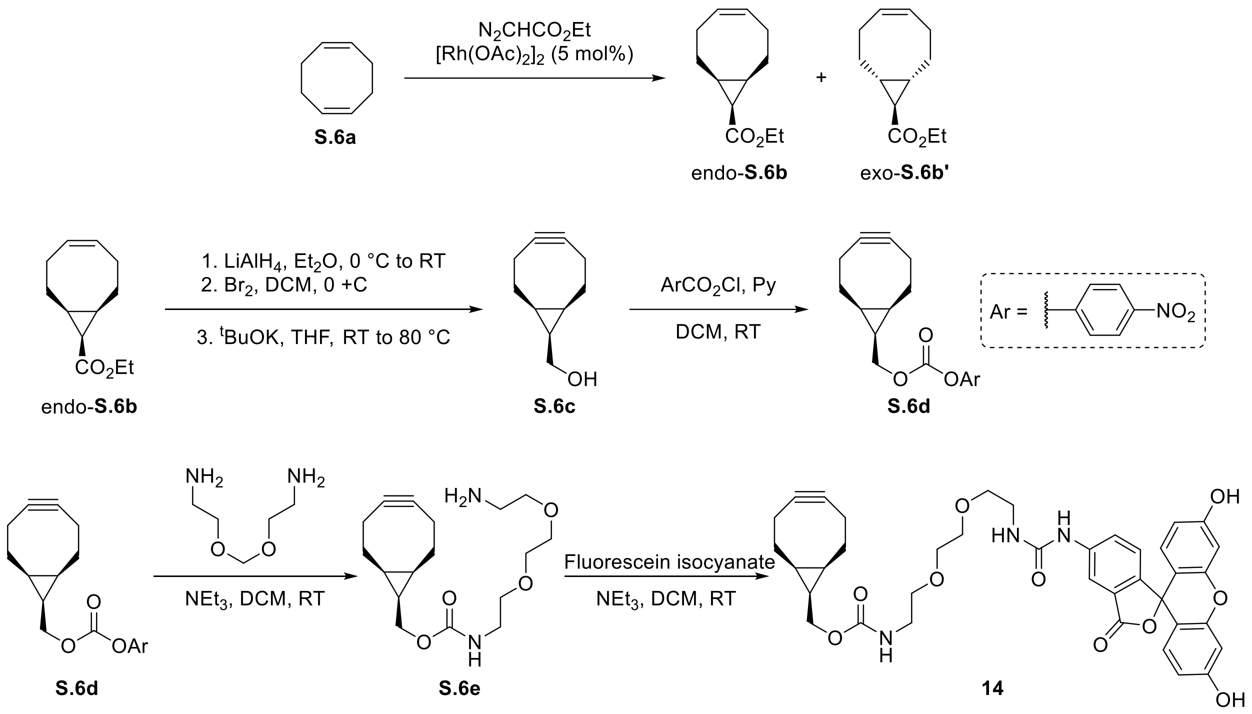

Following a reported procedure[10], to a solution of 1,5-cyclooctadiene **(S.6a)** (19.6 mL, 160 mmol) and $Rh_2(OAc)_4$ (380 mg, 0.86 mmol) CH2Cl2 (10 mL) was added dropwise in 3 h a solution of ethyl diazoacetate (2.1 mL, 20 mmol) in CH2Cl2 (10 mL). This solution was stirred for 40 h at rt. CH2Cl2 was evaporated and the excess of cyclooctadiene was removed by filtration over a glass filter filled with silica and elution with EtOAc:heptane, 1:200 (400 mL). The filtrate was concentrated in vacuo and the residue was purified by column chromatography on silica gel (EtOAc:heptane, 1:20) to afford **endo-S.6b** (1.10 g, 28%) and **exo-S.6b** (2.24 g, 58%) as colorless oils. R_F exo-5 0.24, R_F endo-5 0.33 (EtOAc:heptane, 1:20).

Following a reported procedure,[11] inside a glove-box, a 50 mL two-necked, round-bottomed flask was charged with $LiAlH_4$ (0.10 g, 2.6 mmol, 0.85 equiv.). After the flask was taken out of the glove box, Et_2O (dry; 11.5 mL) was added. The resulting off-white suspension was cooled to 0 °C (ice - water bath), followed by the drop-wise addition of a solution of (Z)-bicyclo[6.1.0]non-4-ene-9-carboxylate (**endo-S.6b**) (0.600 g, 3.09 mmol, 1.0 equiv.) in Et_2O (dry; 11.5 mL). Once the addition was completed, the suspension was stirred at room temperature for 1 hour. It was then cooled back to 0 °C, diluted with Et_2O (20 mL) and the reaction was quenched by addition of water (0.10 mL; release of gas!!), aq. NaOH (15% w/w; 0.10 mL), and water (0.30 mL). The mixture was stirred at room temperature for 30 minutes, becoming completely white. Na_2SO_4 was then added, and stirring was continued for another 10 minutes. The solids were filtered off through a pad of celite, which was then washed with ether and

[10] J. Dommerholt, S. Schmidt, R. Temming, L. J. A. Hendriks, F. P. J. T. Rutjes, J. C. M. van Hest, D. J. Lefeber, P. Friedl, F. L. van Delft, *Angew. Chem. Int. Ed.* **2010**, *49*, 9422–9425.

[11] T. Fiala, J. Wang, M. Dunn, P. Šebej, S. J. Choi, E. C. Nwadibia, E. Fialova, D. M. Martinez, C. E. Cheetham, K. J. Fogle, M. J. Palladino, Z. Freyberg, D. Sulzer, D. Sames, *J. Am. Chem. Soc.* **2020**, *142*, 9285–9301.

EtOAc (overall, 40 mL). The resulting clear filtrate was concentrated under reduced pressure to provide a pale oil, which was used directly in the following step.

The crude oil obtained from the previous step was dissolved in DCM (22 mL). The resulting colorless solution was cooled to 0 °C (ice - water bath) under stirring. A solution of bromine (0.20 mL, 3.7 mmol, 1.2 equiv.) in DCM (2.3 mL) was then added drop-wise: the resulting mixture rapidly decolored upon the addition of each drop until ca. 1.5 mL of the bromine solution was added; the solution then remained yellow-orange. The reaction was then quickly quenched by addition sat. aq. $Na_2S_2O_3$ (40 mL) under vigorous stirring, resulting in the solution becoming colorless. Upon the separation of the aqueous layer, the latter was extracted with DCM (2 x 30 mL). The combined organic layers were washed with brine, dried over Na_2SO_4, filtered and concentrated under vacuum to furnish an off-white solid. The latter was dissolved in THF (dry; 33 mL) inside a 100 mL single-necked, round-bottomed flask. At room temperature, a solution of potassium *tert*-butoxide (1.2 g, 11 mmol, 3.5 equiv.) in THF (11 mL) was added drop-wise to the solution, which immediately became yellow-orange, and then further turned into an orange-brown, turbid suspension as the addition continued. Once the addition was completed, the flask was rapidly equipped with an air-condenser, and the orange-brown suspension was refluxed (heating block at 85 °) for 4 hours. After allowing the mixture to cool down to room temperature, the reaction was quenched with sat. aq. NH_4Cl (20 mL), and the aqueous layer was extracted with DCM (3 x 20 mL). The combined organic layers were dried over Na_2SO_4, filtered, and concentrated *in vacuo*. The yellow-orange crude oil was submitted to column chromatography (SiO_2; EtOAc in pentane, 1/4 to 1/1) to provide bicyclo[6.1.0]non-4-yn-9-ylmethanol (**S.6c**) (0.193 g, 1.28 mmol, 36% yield over 3 steps) as a pale yellow solid.

Following a reported procedure,[8] under nitrogen, in a 50 mL two-necked, round-bottomed flask, bicyclo[6.1.0]non-4-yn-9-yl)methanol (**S.6c**) (0.095 g, 0.63 mmol, 1.0 equiv.) was dissolved in DCM (dry; 14.8 mL). p-Nitrophenyl chloroformate (0.16 g, 0.79 mmol, 1.25 equiv.) was added in a single portion, resulting in the formation of a homogeneous suspension. To the latter, pyridine (dry; 0.13 mL, 1.6 mmol, 2.5 equiv.) was finally added. The mixture was stirred at room temperature for 30 minutes. It was then quenched by addition of sat. aq. NH_4Cl (20 mL). After being separated, the aqueous layer was extracted with DCM (3 x 20 mL). The combined organic layers were washed with brine, dried over Na_2SO_4, filtered, and concentrated under vacuum. The pale yellow crude residue was submitted to column chromatography (SiO_2; Hexane/EtOAc 9/1 to 8/2) to provide bicyclo[6.1.0]non-4-yn-9-yl)methyl (4-nitrophenyl) carbonate (**S.6d**) (0.164 g, 0.520 mmol, 82% yield) as a colorless oil, which became a solid on standing.

Following a reported procedure,[12] under nitrogen, in a 50 mL two-necked, round-bottomed flask, bicyclo[6.1.0]non-4-yn-9-ylmethyl (4-nitrophenyl) carbonate (**S.6d**) (0.160 g, 0.507 mmol, 1.0 equiv.) and 2,2'-(ethane-1,2-diylbis(oxy))bis(ethan-1-amine) (0.74 mL, 5.1 mmol, 10 equiv.) were dissolved in DCM (dry; 13 mL). At room temperature, triethylamine (0.35 mL, 2.5 mmol, 5.0 equiv.) was added drop-wise: immediately, the solution turned from colorless to bright yellow. It was stirred at room temperature overnight. The solution was than washed with aq. NaOH (1.0 M; 2 x 15 mL), leading to the decoloring of the organic layer. The combined aqueous layers were extracted with DCM (3 x 20 mL). The combined organic layers were washed with water (2 x 15 mL), brine, dried over Na_2SO_4,

[12] G.-J. Boons, X. Liu WO2020/263943, **2020**, A1

filtered, and concentrated under vacuum. The resulting yellow crude oil was submitted to column chromatography (SiO_2; DCM/MeOH 95/5 to 15/85 + Et_3N 1% v/v) to provide bicyclo[6.1.0]non-4-yn-9-ylmethyl (2-(2-(2-aminoethoxy)ethoxy)ethyl)carbamate (**S.6e**) (0.154 g, 0.474 mmol, 94% yield) as a yellow oil.

Following a slightly modified procedure,[13] under a nitrogen atmosphere, to bicyclo[6.1.0]non-4-yn-9-ylmethyl (2-(2-(2-aminoethoxy)ethoxy)ethyl)carbamate (**S.6e**) (30.0 mg, 92.5.0 µmol, 1.00 equiv) in DMF (2.10 mL) was added Fluorescein isocyanate (39.6 mg, 102 µmol, 1.10 equiv). After 16 hours, the reaction was dilute with DCM and evaporated under reduced pressure. The residue was purified by reverse phase preparative HPLC (water 0.1 % TFA to 95:5 ACN:water 0.1 % TFA) to afford **14** (46.0 mg, 64.4 µmol, 70%) as an orange solid after lyophilization.

^{1}H NMR (CD_3OD, 400 MHz) δ 8.19 (d, *J* = 1.8 Hz, 1H, Ar*H*), 7.78 (dd, *J* = 7.8, 1.2 Hz, 1H, Ar*H*), 7.15 (d, *J* = 8.1 Hz, 1H, Ar*H*), 6.70 (s, 1H, Ar*H*), 6.68 – 6.67 (m, 2H, Ar*H*), 6.67 (s, 1H, Ar*H*), 6.56 (d, *J* = 2.4 Hz, 1H, Ar*H*), 6.53 (d, *J* = 2.4 Hz, 1H, Ar*H*), 4.09 (d, *J* = 8.1 Hz, 2H, CH_2BCN), 3.86 – 3.77 (m, 2H, CH_2-PEG), 3.71 (t, *J* = 5.1 Hz, 2H, CH_2-PEG), 3.68 – 3.63 (m, 4H, CH_2-PEG), 3.53 (t, *J* = 5.6 Hz, 2H, CH_2-PEG), 3.27 (t, *J* = 5.6 Hz, 2H, CH_2-PEG), 2.24 – 2.18 (m, 2H, CH_2-BCN), 2.10 – 2.06 (m, 1H, C*H*-BCN), 1.65 – 1.46 (m, 2H, CH_2-BCN), 1.40 – 1.23 (m, 4H, CH_2-BCN, C*H*-BCN), 0.97 – 0.81 (m, 2H, CH_2-BCN).
HRMS (ESI): m/z calculated $[M+H]^+$ = 714.0, found $[M+H]^+$ = 714.2.
Spectroscopic data was consistent with the values reported in literature.[14]

General procedure for SPAAC:
In a 1.5 mL Eppendorf Safe-Lock microcentrifuge, KEAP1 (**8a**) (32 µM in PBS stock solution, 25 µL) was diluted in Tris buffer or PBS buffer (25 mM, pH 8, 55 µL). Then a 1 mM solution of EBX in Tris buffer (25 mM, pH 8, 1.2 µL, 1.5 equiv.) was added in one portion and the resulting mixture was shaken at RT for 1 h. Then, a solution of BCN-dye **14** in DMSO (2.4 µL, 3.0 equiv., 1 mM) was added. The resulting solution was vortexed few seconds to ensure proper reagent mixing and incubated at RT for 1 h. No effort was made to exclude oxygen.

[13] S. M. DeGuire, D. C. Earl, Y. Du, B. A. Crews, A. T. Jacobs, A. Ustione, C. Daniel, K. M. Chong, L. J. Marnett, D. W. Piston, B. O. Bachmann, G. A. Sulikowski, Angewandte Chemie International Edition 2015, 54, 961–964.
[14] P. M. S. D. Cal, R. F. M. Frade, C. Cordeiro, P. M. P. Gois, Chem. Eur. J. 2015, 21, 8182–8187.

EBX **4f**

Ions series:

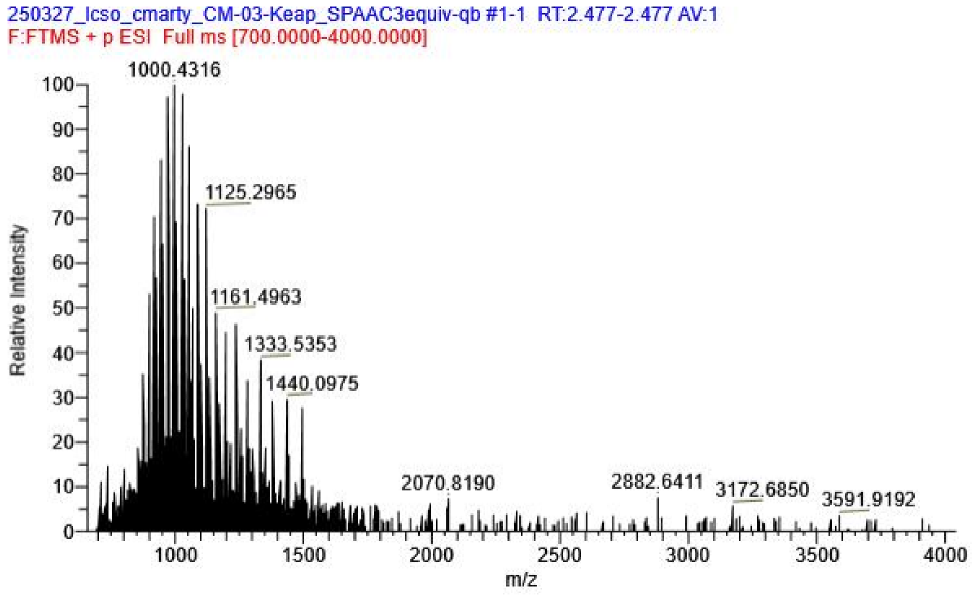

Deconvoluted mass spectrum:

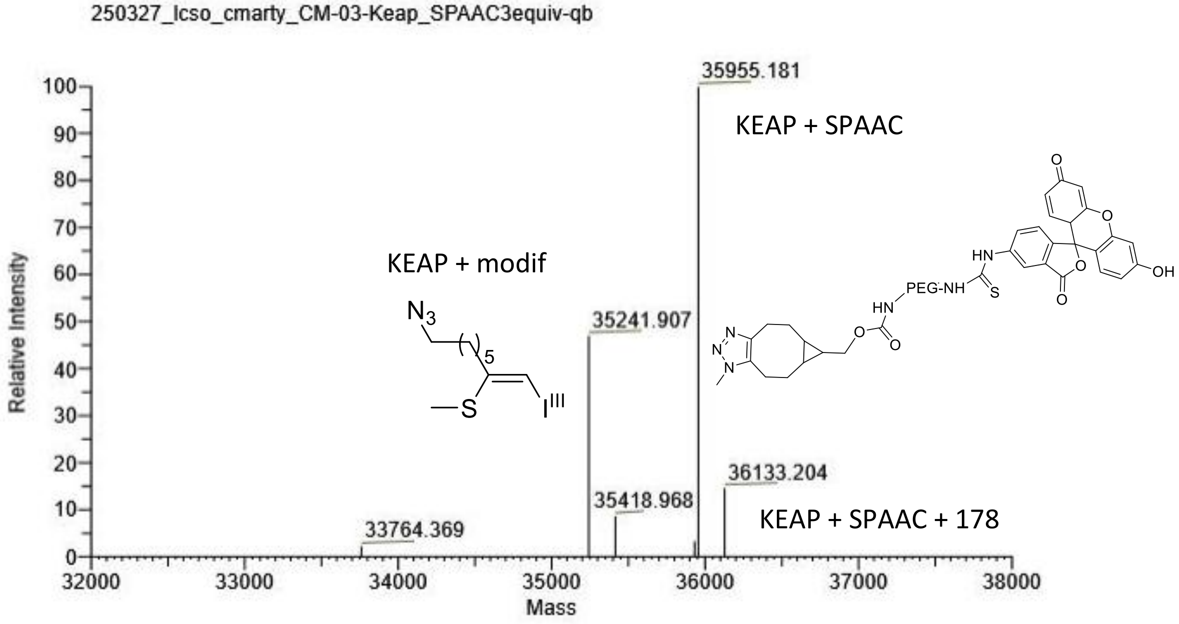

*Figure S29. Combined ions series and deconvoluted mass spectra of the modification with EBX **4f** followed by BCN-dye **14** for SPAAC reaction on KEAP WT. Calcd. Mass, 35954 Da, found: 35955 Da. Conversion 64% from the S-VBX form. (Monoisotopic mass)*

EBX **4cb**

Ions series:

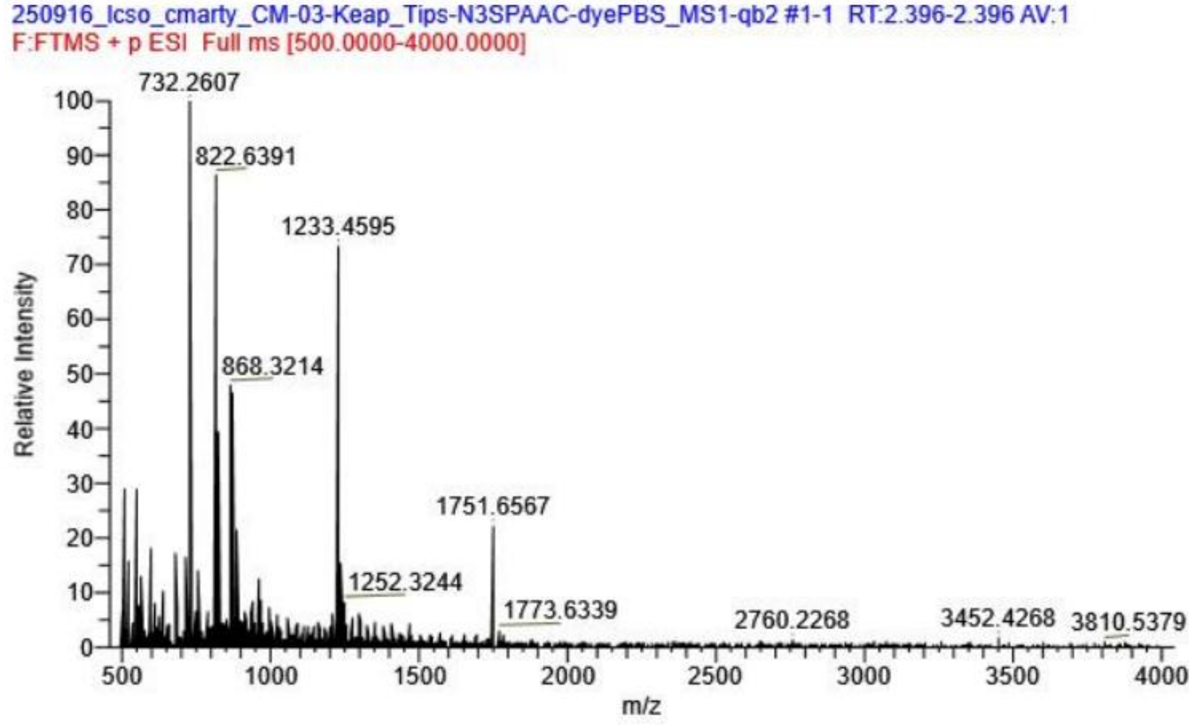

Deconvoluted mass spectrum:

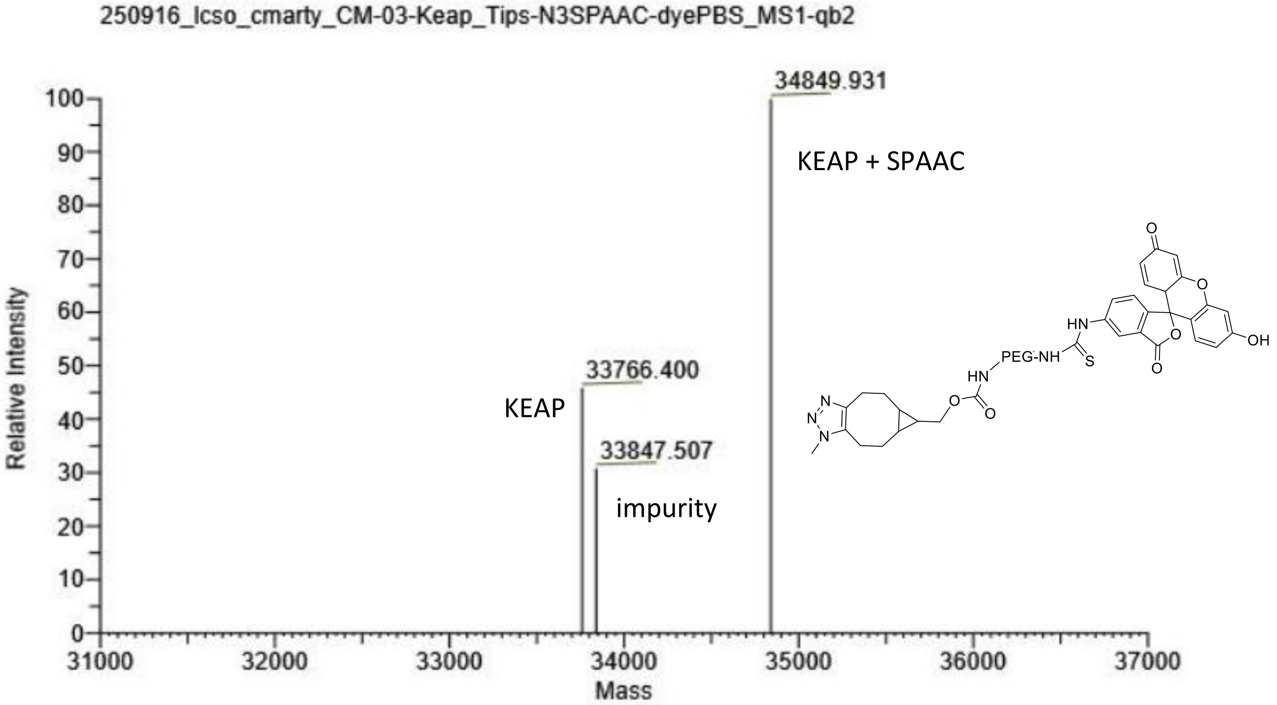

*Figure S30. Combined ions series and deconvoluted mass spectra of the modification with EBX **4cb** followed by BCN-dye **14** for SPAAC reaction on KEAP WT. Calcd. Mass, 34851Da, found: 34849 Da. Conversion 76 % from the alkynylated intermediate. (Monoisotopic mass).*

Note: This conversion was measured following the equation

Conversion = peak (SPAAC)+ peak (SPAAC +178)/ [All peaks – Peak (KEAP WT)]

b. Reduction

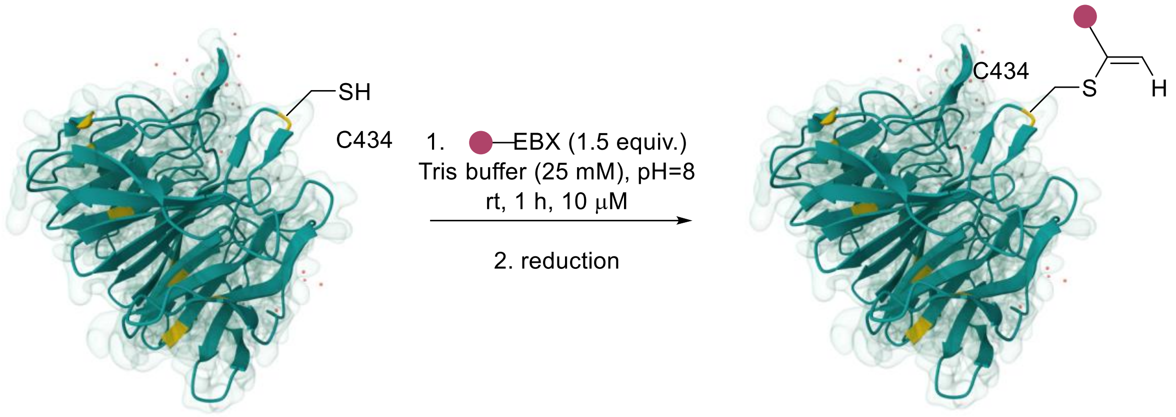

<u>Preparation of palladium complexe **Pd-1**:</u>

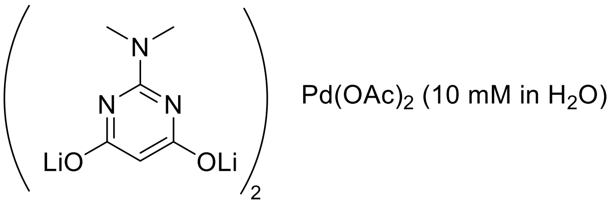

Palladium catalyst **Pd-1** was synthesized following a reported procedure.[15] The 2-amino-4,6dihydroxypyrimidine ligand (20.0 µmol) was dissolved in an aqueous solution of LiOH (0.4 mL, 0.1 M) in an ultrasonic bath for 2 minutes. Then $Pd(OAc)_2$ (10.0 µmol) was added and the mixture was magnetically stirred at 65 °C for 30 minutes, deionized water (0.6 mL) was then added to afford a 10.0 mM catalyst solution of **Pd-1**.

<u>Optimization procedure:</u>

In a 1.5 mL Eppendorf Safe-Lock microcentrifuge, KEAP (**8a**) (32 μM in PBS stock solution, 25 μL) was diluted in buffer (25 mM, pH 8, 55 μL). Then a solution of EBX (1 mM) in buffer (25 mM, pH 8, 1.2 μL) was added in one portion and the resulting mixture was shaken at RT for 1 h. Then, a solution of hydride source (in water) was added followed by the addition of a solution of palladium complex (**Pd-1**, 1 mM on water, 8.0 μL, 10 equiv.). The resulting solution was vortexed few seconds to ensure proper reagent mixing and incubated at 37 °C for 1 hour. No effort was made to exclude oxygen. The reaction was quenched with a 10 mM solution of 3-mercaptopropionic acid in water (5.00 equiv. per equiv. of palladium) and shaken at room temperature for 10 minutes.

[15] Chalker, J.; Wood, C.; Davis, B. J. Am. Chem. Soc. 2009, 131, 16346

Table 2. Optimization of the ligand removal

Entry	Pd (equiv.)	Hydride source	Reduction[a]	Over-reduction (WT again)	Conversion
1	10	NaCOOH (20 equiv.)	23 %	-	34 %
2	10	NEt_3/HCOOH (30 equiv.)	16 %		35 %
3	20	NaCOOH (60 equiv.)	16 %		36 %
4	5	TES (15 equiv.)	16 %	-	38 %
5	5	NaAsc (15 equiv.) + $NaBH_4$ (30 equiv.)	16 %	16 %	47 %
6	10	NaCOOH (30 equiv.) in PB	- -	71 %	91%

a. Yield was determined by HRMS

a. In tris buffer

Procedure:

In a 1.5 mL Eppendorf Safe-Lock microcentrifuge, KEAP (**8a**) (32 μM in PBS stock solution, 25 μL) was diluted in Tris buffer (25 mM, pH 8, 55 μL). Then a 1 mM solution of EBX in Tris buffer (25 mM, pH 8, 1.2 μL) was added in one portion and the resulting mixture was shaken at RT for 1 h. Then, a solution of NaCOOH (in water, 3.2 μL, 20 equiv., 5 mM) was added followed by the addition of a solution of **Pd-1** (8 μL, 10 equiv., 1mM in water). The resulting solution was vortexed few seconds to ensure proper reagent mixing and incubated at 37 °C for 1 hour. No effort was made to exclude oxygen. The reaction was quenched with a 10 mM solution of 3-mercaptopropionic acid in water (4 μL, 5.00 equiv. per equiv. of palladium) and shaken at room temperature for 10 minutes.

Ions series:

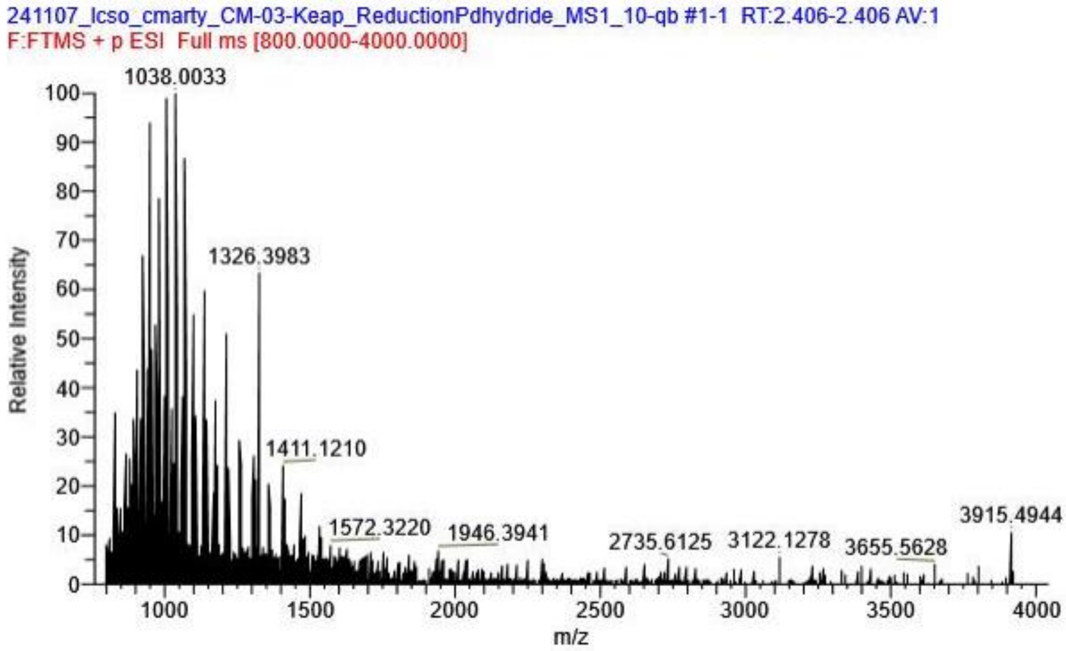

Deconvoluted mass spectrum:

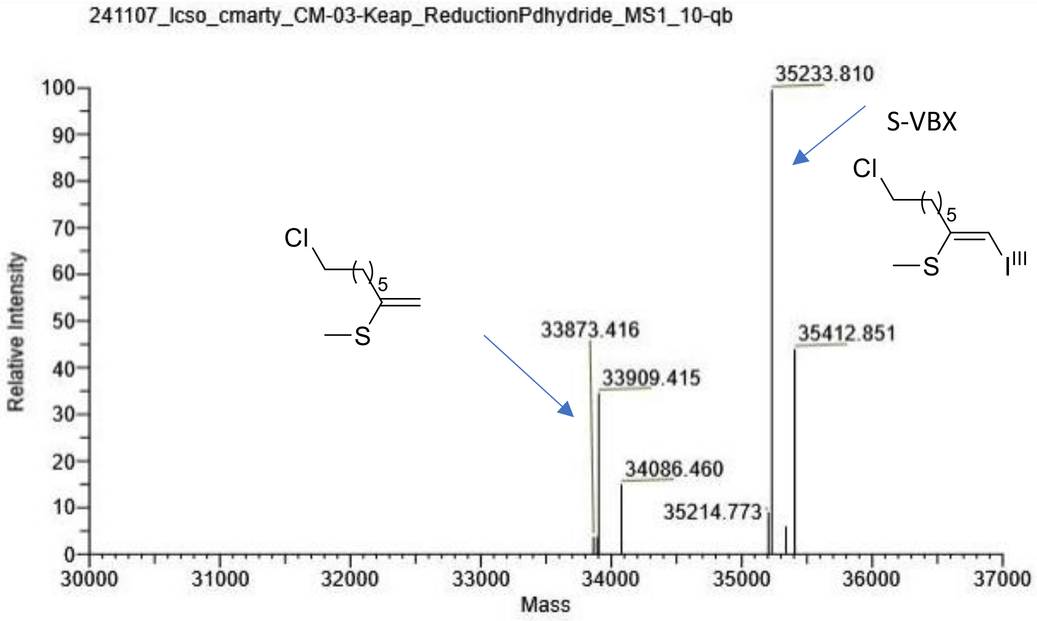

*Figure S31. Combined ions series and deconvoluted mass spectra of the modification with EBX **4e** on KEAP WT. Calcd. Mass, 33909 Da, found: 33909 Da. Conversion 23% from the S-VBX form. (Monoisotopic mass)*

b. In PB buffer

Procedure:
In a 1.5 mL Eppendorf Safe-Lock microcentrifuge, KEAP (**8a**) (32 µM in PBS stock solution, 25 µL) was diluted in PB buffer (25 mM, pH 8, 55 µL). Then a 1 mM solution of EBX in PB buffer (25 mM, pH 8, 1.2 µL) was added in one portion and the resulting mixture was shaken at RT for 1 h. Then, a 10 mM solution of NaCOOH (2.4 µL, 30 equiv., 10 mM in water) was added followed by the addition of a solution of **Pd-1** (8 µL, 10 equiv., 1 mM in water). The resulting solution was vortexed few seconds to ensure proper reagent mixing and incubated at 37 °C for 1 hour. No effort was made to exclude oxygen. The reaction was quenched with a 10 mM solution of 3-mercaptopropionic acid in water (4 µL, 5.00 equiv. per equiv. of palladium) and shaken at room temperature for 10 minutes.

Ions series:

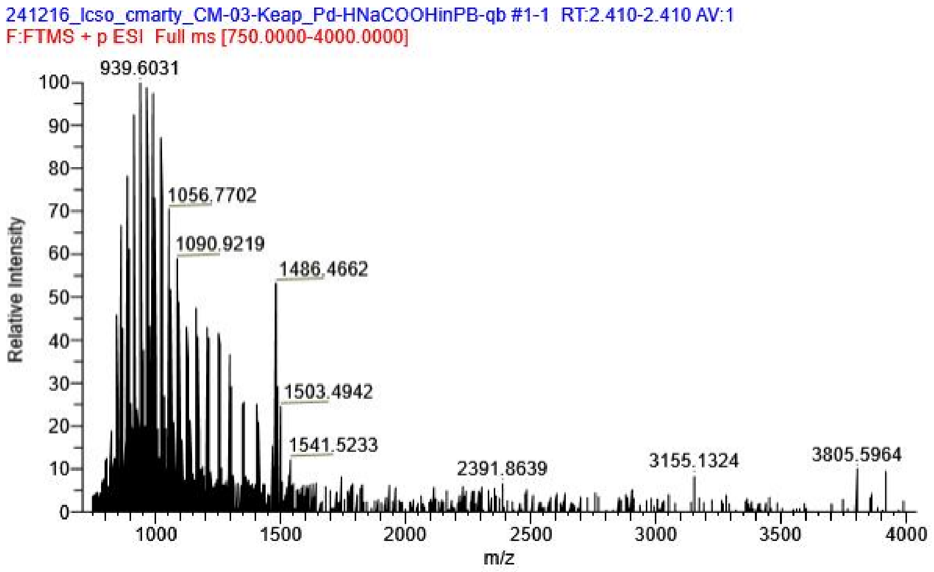

Deconvoluted mass spectrum:

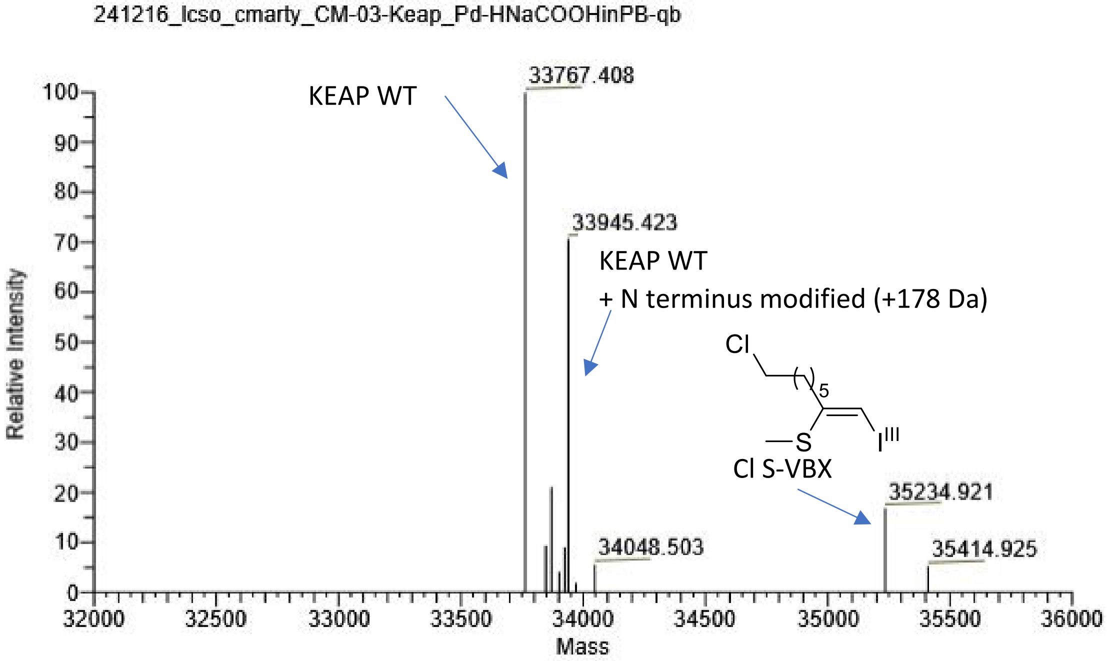

*Figure S32. Combined ions series and deconvoluted mass spectra of the modification with EBX **4e** on KEAP WT. Calcd. Mass, 35240 Da, found: 35240 Da. Conversion 69% from the S-VBX form. (Monoisotopic mass)*

c. Bioconjugation in a mixture 1:1 BSA/KEAP1, competition experiment

Procedure:

In a 1.5 mL Eppendorf Safe-Lock microcentrifuge, KEAP1 (**8a**) (32 μM in PBS stock solution, 25 μL) and BSA (33 μM in PBS stock solution, 25 μL) was diluted in buffer (25 mM, pH 8, 55 μL). Then a solution of EBX (1 mM) in Tris buffer (25 mM, pH 8, 1.2 μL) was added in one portion. The resulting solution was vortexed few seconds to ensure proper reagent mixing and incubated at RT for 1 hour. No effort was made to exclude oxygen.

Ions series:

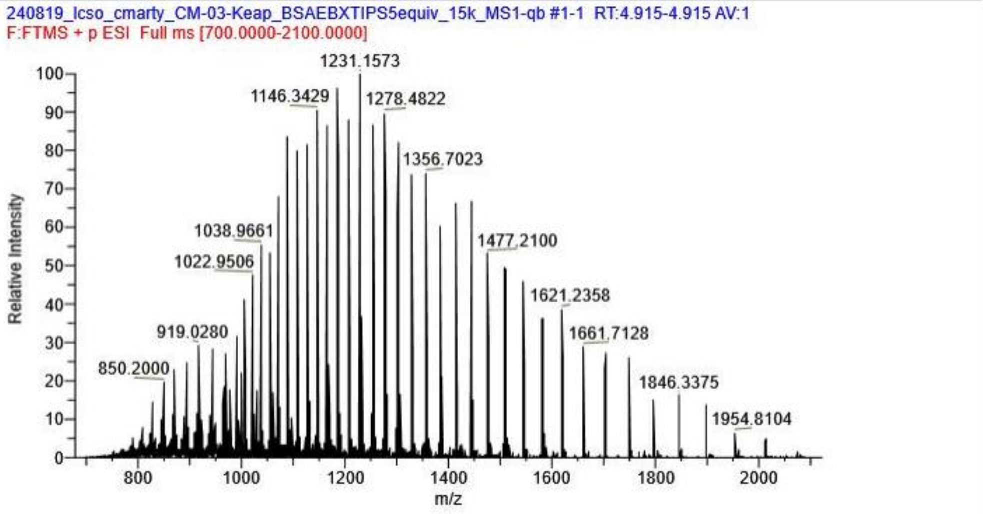

Deconvoluted mass spectrum:

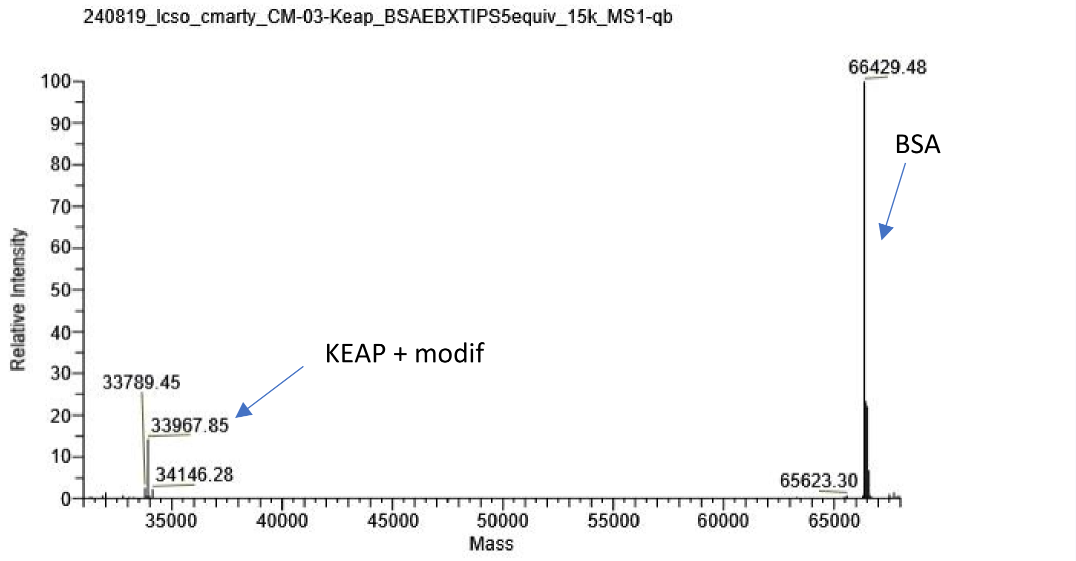

*Figure S33. Combined ions series and deconvoluted mass spectra of the modification with EBX **4aa** on KEAP WT. Calcd. Mass, 33965 Da, found: 33967 Da. (Average Mass) Conversion 77% for KEAP, no conversion for BSA.*

8. Cell lysate

Procedure:

Pierce IP lysis Buffer

The culture medium was removed from cells (U2OS). The cells were washed once with ice cold phosphate-buffered saline. 3.5 million cells were counted manually by hemocytometer therefore 350uL of lysis buffer (25 mM Tris-HCl pH=7.4, 150 mM NaCl, 1% NP-40, 1 mM EDTA, 5% glycerol, and protease inhibitors) was added to the cell and incubated on ice for 5 minutes. Then the lysate was transferred to a microcentrifuge tube and centrifuge at ~13,000 × g for 10 minutes to pellet the cell debris at 4 °C. Finally, the supernatant was transferred to a new tube for protein concentration determination.

Bradford

The cell lysate was diluted by 10 with the lysis buffer. Stock solutions of BSA were prepared in the lysis buffer (Set of 7 concentrations of BSA (2, 1.5, 1, 0.75, 0.5, 0.25, 0.125 mg/mL) in 2 ml tubes. In a microplate, in each well was added 250uL of Dye reagent + 5uL volume of sample (BSA stock solution and cell lysate with several dilution). Triplicate were made for the calibration curve. The microplate was centrifuged. Then, the microplate was incubated at room temperature for at least 5 min and the microplate was read by a plate ridder with the spectrophotometer set to 595 nm.

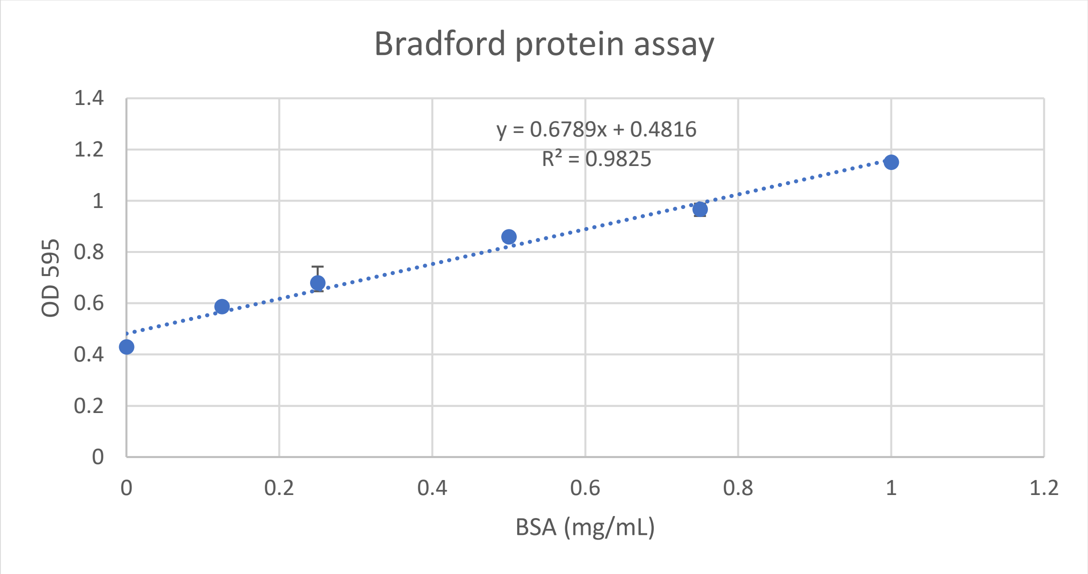

Figure S34. Bradford protein assay curve.

The cell lysate gave an OD of 0.6811 and 0.6907 which correspond to 1.85 mg/mL after removing the absorbance coming from the Pierce IP buffer.

Cell lysate

In a 1.5 mL Eppendorf Safe-Lock microcentrifuge, KEAP (32 μM in PBS stock solution, 25 μL) was diluted in buffer (25 mM, pH 8, 55 μL) or cell lysate (diluted by 2 with Tris buffer, 0.9 mg/mL). Then a solution of EBX **4cb** (1 mM) in buffer (25 mM, pH 8, 1.2 μL) was added in one portion and the resulting mixture was shook at RT for 1 h. Then, a 1 solution of BCN-dye **(14)** (in DMSO, 1 mM, 2.4 μL, 3 equiv.) was added. The resulting solution was vortexed few seconds to ensure proper reagent mixing and incubated at 37 °C for 1 hour. No effort was made to exclude oxygen.

KEAP (**8a**)	+	+	-	+	+
EBX (**4cb**)	+	+	+	-	-
Cell lysate	+	-	+	+	-

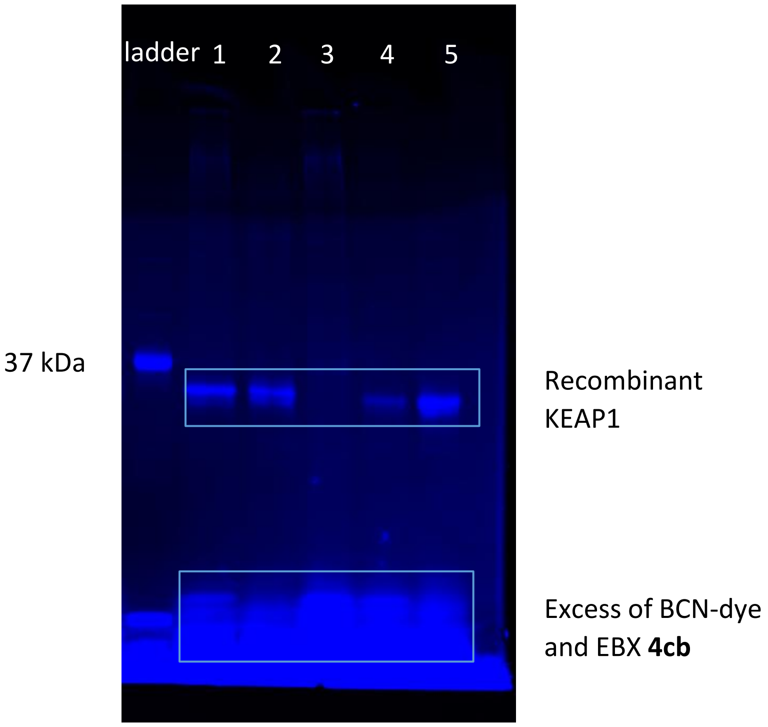

*Figure S35. Fluorescence image (FL) of sodium dodecyl sulfate-polyacrylamide gel electrophoresis (SDS-PAGE) of the reaction mixture between Kelch domain of KEAP1 and EBX **4cb** in 0.9 mg/mL U2OS cell lysate. The ladder used was precision Plus Protein Kaleidoscope Standards from BioRad.*

Reaction conditions: 25 mM Tris buffer, pH 8, 21 °C, 0.9 mg/mL HeLa cell lysate.

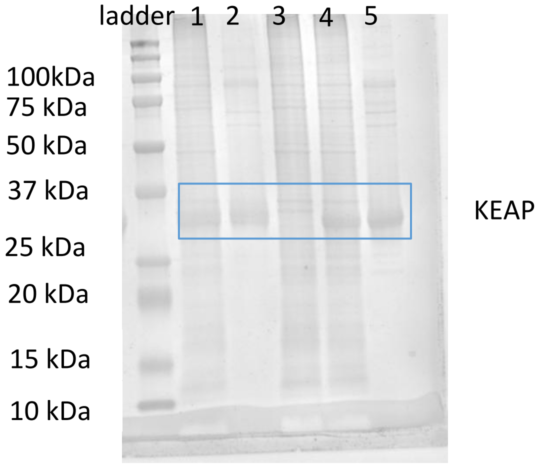

*Figure S36. Coomassie stained of sodium dodecyl sulfate-polyacrylamide gel electrophoresis (SDS-PAGE) of the reaction mixture between KEAP and EBX **4cb** in 0.9 mg/mL U2OS cell lysate. The ladder used was precision Plus Protein Kaleidoscope Standards from BioRad.*

Reaction conditions: 25 mM Tris buffer, pH 8, 21 °C, 0.9 mg/mL HeLa cell lysate.

Mass of well 2

Ions series:

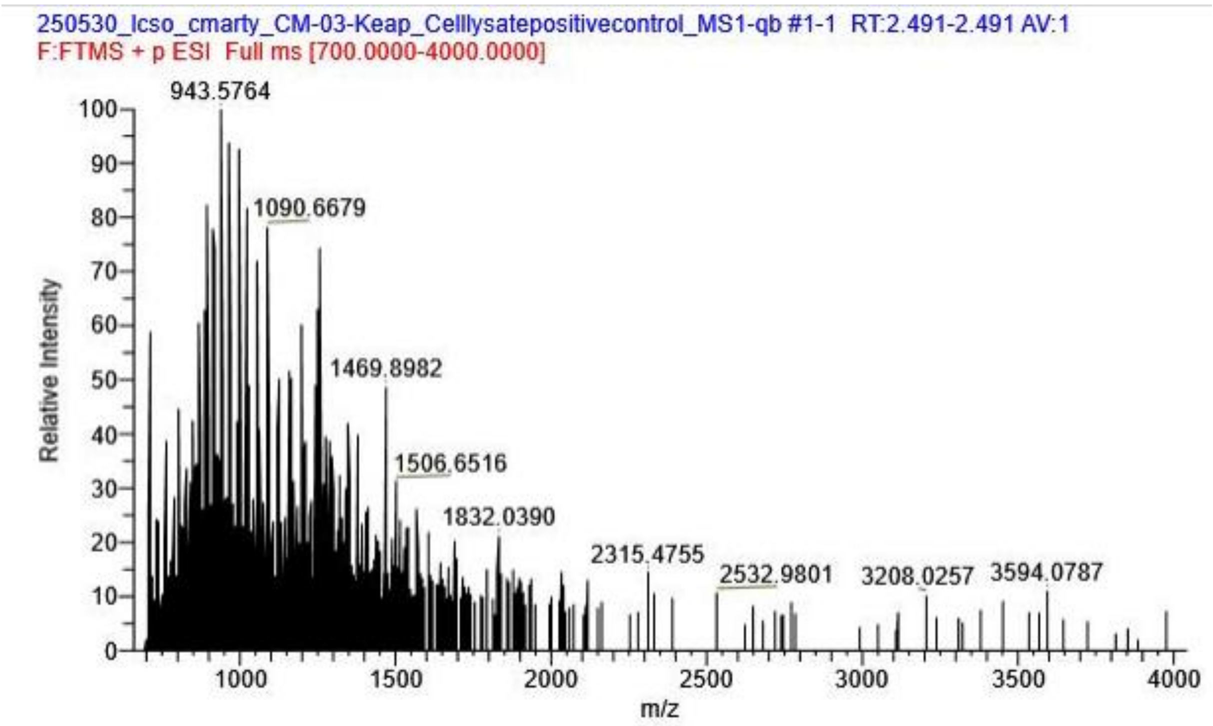

Deconvoluted mass spectrum:

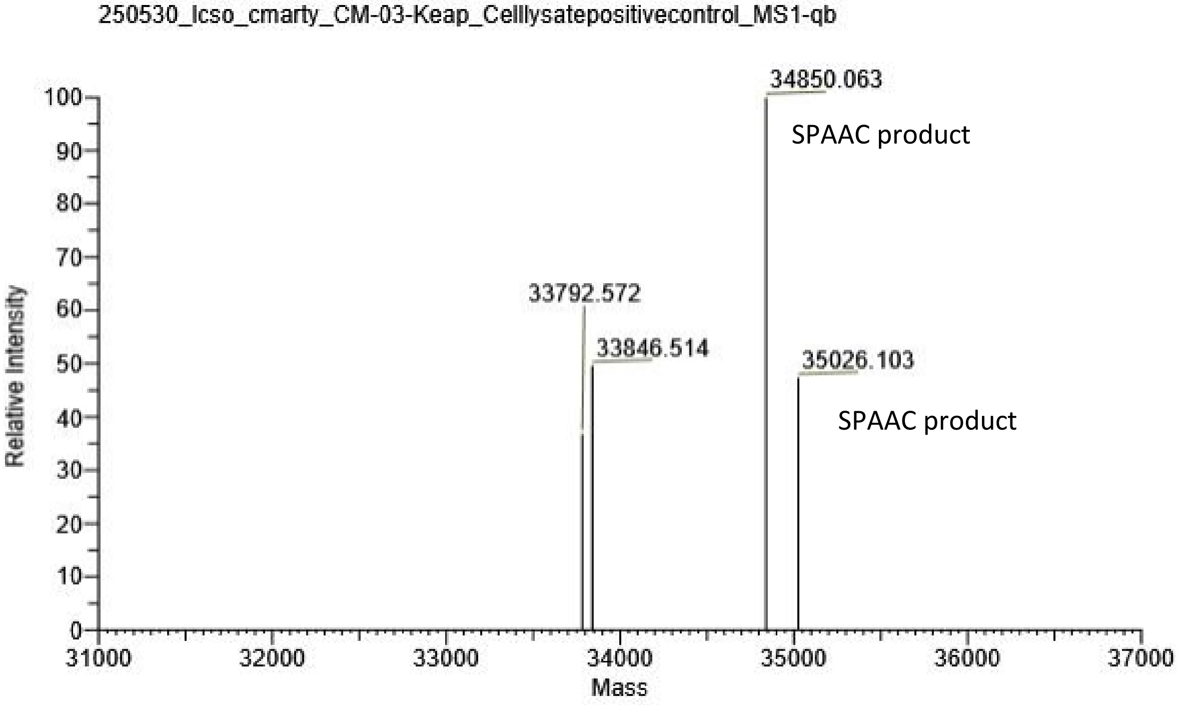

Figure S37. Combined ions series and deconvoluted mass spectra of the positive control of the cell lysate. Modification with EBX ***4cb*** *followed by BCN-dye (**14**) SPAAC reaction on KEAP WT. Calcd. Mass, 34851Da, found: 34850 Da (Monoisotopic mass)*

Mass of well 5

Ions series:

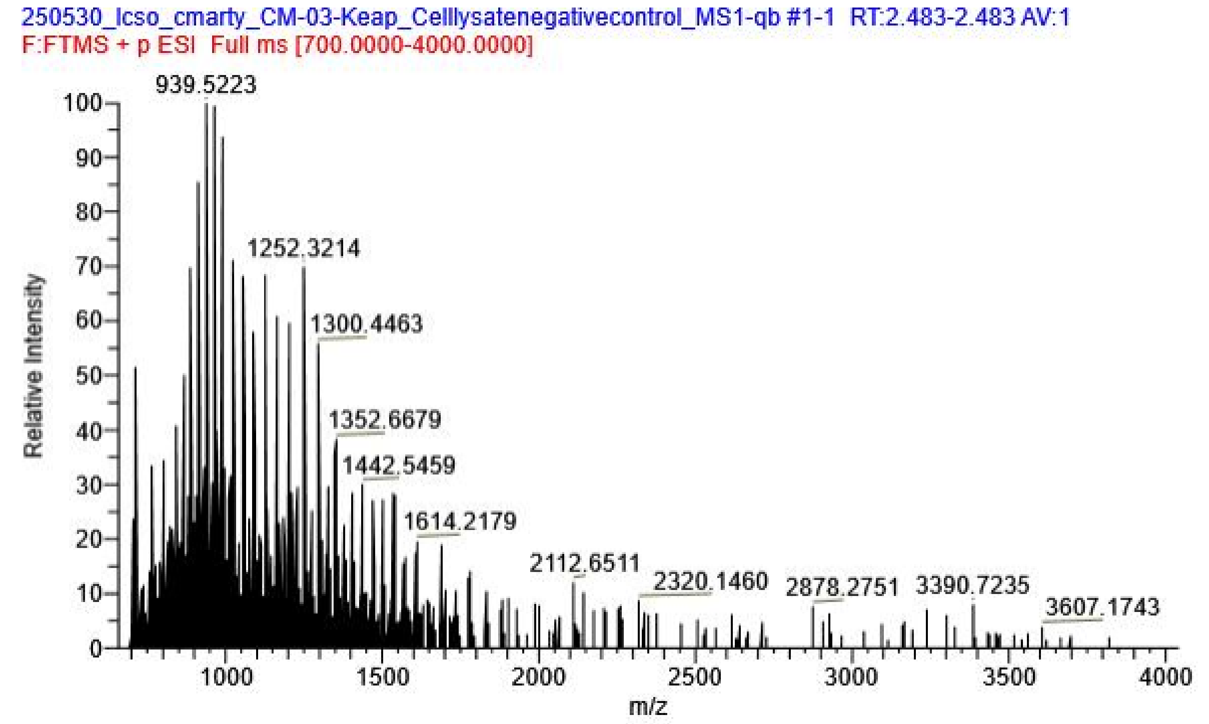

Deconvoluted mass spectrum:

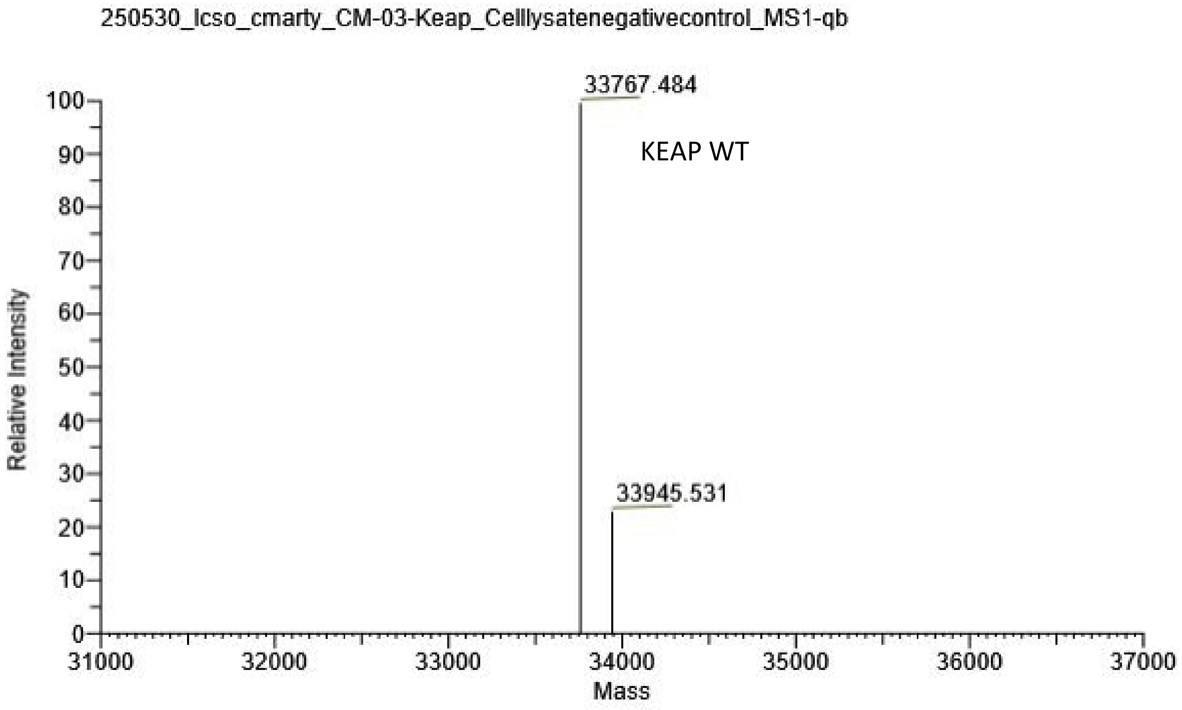

*Figure S38. Combined ions series and deconvoluted mass spectra of the negative control with cell lysate. Modification with BCN-dye (**14**) on KEAP WT. No modification observed. (Monoisotopic mass)*

9. Top-down experiment mass spectrometry analysis

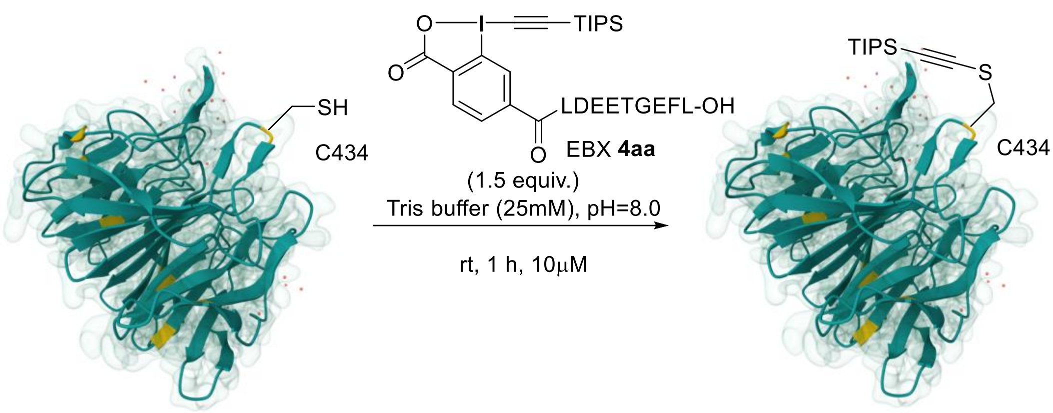

To localize the modification sites, sample was submitted to top-down mass spectrometry analysis using higher energy collision induced dissociation (HCD). Sample was diluted to a final protein concentration of 1.5 uM with milliQ water containing 0.1% formic acid and were injected into an Acquity UPLC Protein column BEH C4 VanGuard (300 Å, 1.7 µm, 2.1 x 5 mm, Waters, Milford, MA, U.S.A.) using a Vanquish analytical LC system (Thermo Fisher Scientific, Germany) coupled to an OptaMax NG ion source (Thermo Fisher Scientific, Bremen, Germany). The sample desalting was performed with a flow rate of 400 µl/min by applying a gradient of solvent B from 15 to 60 % in 2.5 min, followed by column washing and re-equilibration steps. Solvent A was composed of milliQ water with 0.1 % formic acid, while solvent B consisted of acetonitrile with 0.1 % formic acid. Eluting proteoforms were analyzed on a Orbitrap Exploris 240 FT-MS benchtop instrument (Thermo Fisher Scientific, Bremen, Germany) using Intact Protein mode with low pressure settings, positive polarity and targeted MS/MS approach with ion multiplexing. Six precursor ions corresponding to different charge states of the same proteoform carrying one EBX modification were included into the targeted inclusion mass list. Isolation window was set to 1.2 m/z, AGC target as standard, maximum IT set as Auto, 240’000 resolution at 200 m/z and averaging 10 microscans. Top-down analysis was repeated using 6 different values (between 20-50 %) of normalized collision energy (NCE). Top-down MS data were processed using Peak-by-Peak software with MS deconvolution workflow (Spectroswiss, Lausanne, Switzerland).

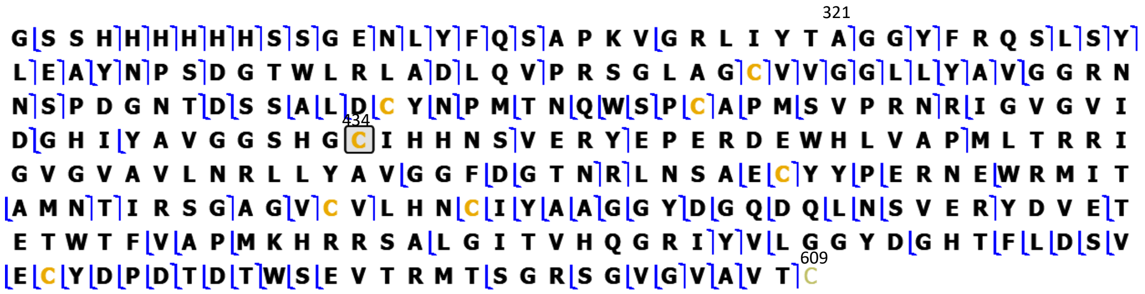

Figure S39. Fragmentation map with assigned b- and y-fragment ions, obtained from top-down analysis by LC-MS/MS with 6 different NCEs of HCD. Achieved sequence coverage is 44.8 % with 10 ppm mass accuracy for fragment assignment. The modified residues are boxed by a black square, in this case only C434 was modified.

10. NMR spectrum

(3-(4-(2-Azidoethyl)phenethoxy)propyl)di*iso*propyl((trimethylsilyl)ethynyl)silane (S1)

^{1}H NMR (400 MHz, $CDCl_3$)

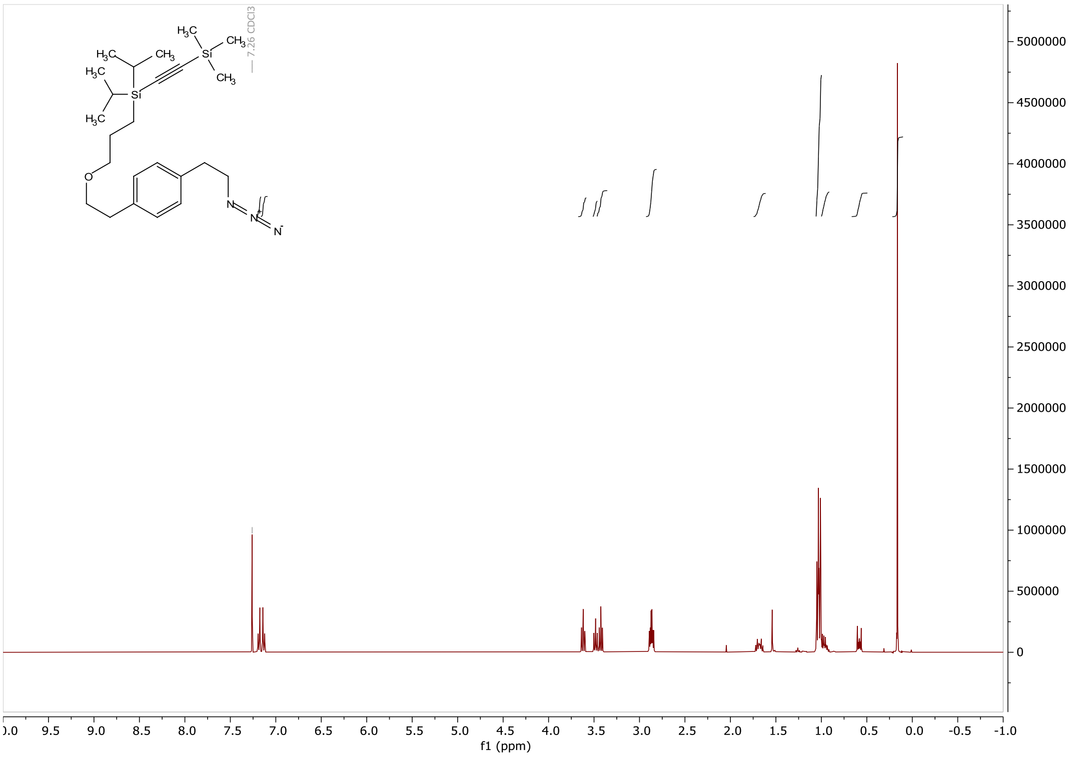

^{13}C NMR (101 MHz, $CDCl_3$)

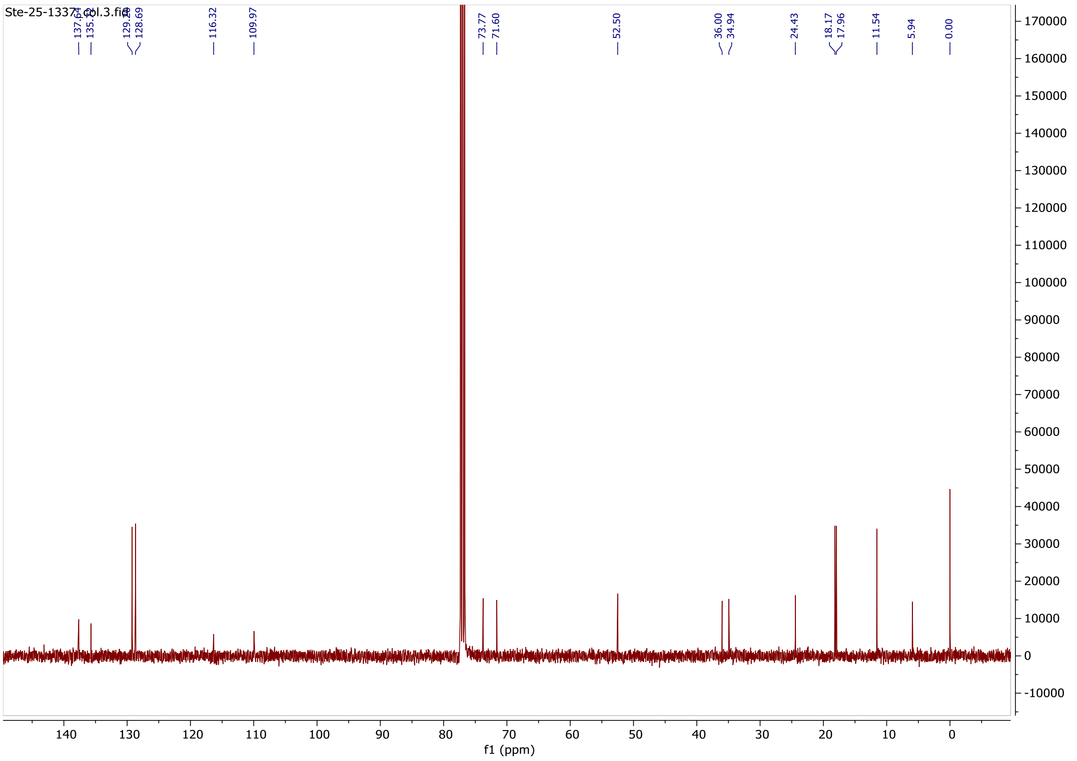

8-chlorooct-1-ynyl(trimethyl)silane (S2)

^1H NMR (400 MHz, $CDCl_3$)

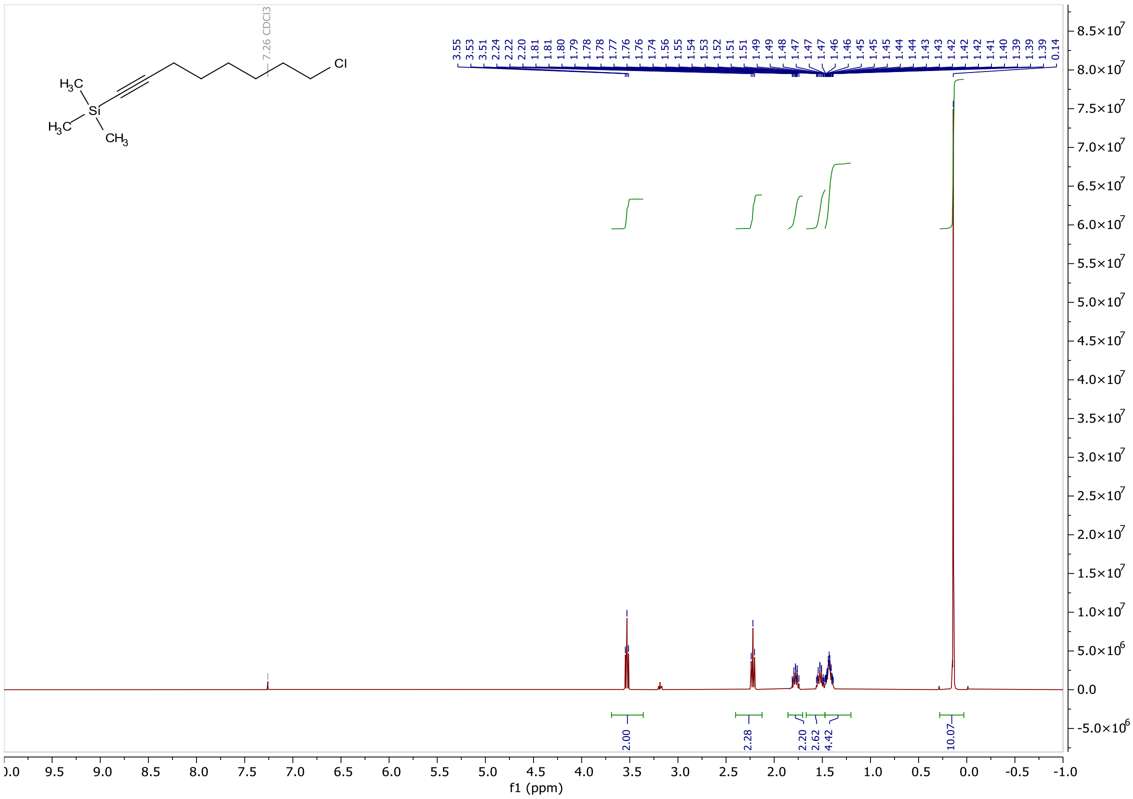

^{13}C NMR (101 MHz, $CDCl_3$)

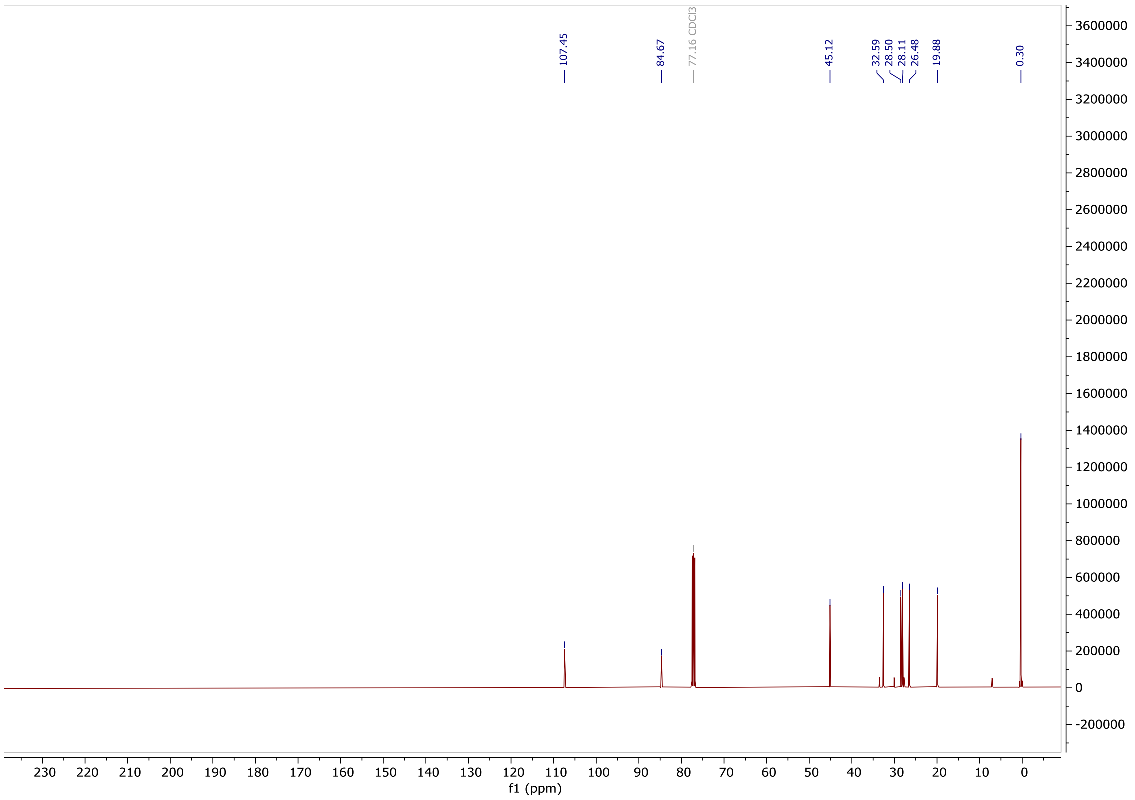

8-(trimethylsilyl)oct-7-yn-1-ol (S3)

^1H NMR (400 MHz, $CDCl_3$)

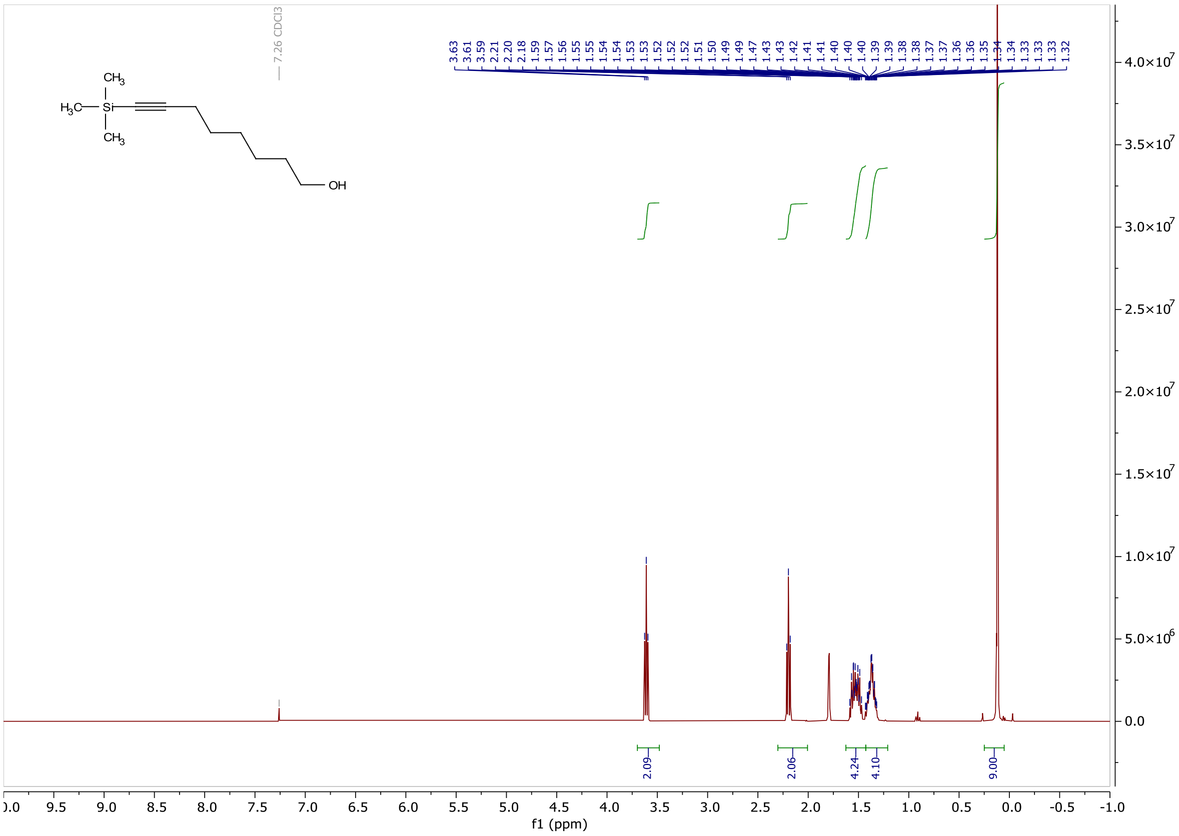

^{13}C NMR (101 MHz, $CDCl_3$)

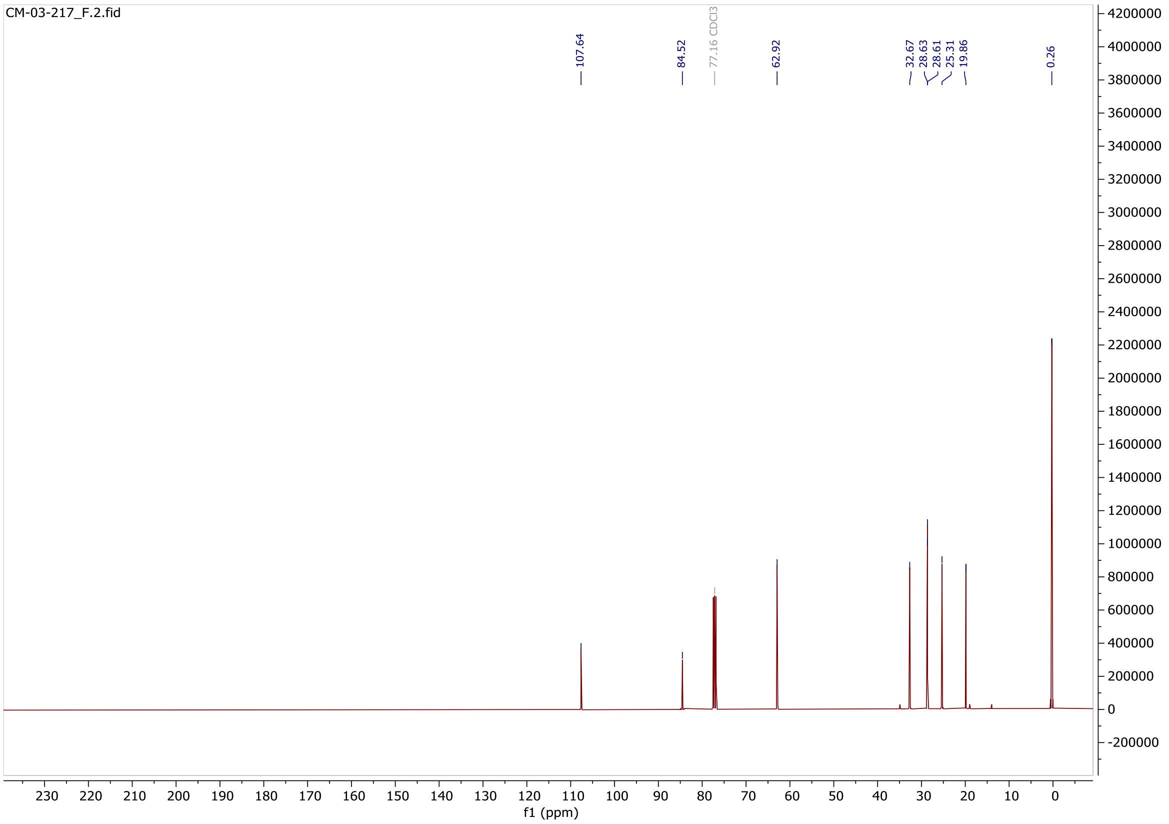

(8-iodooct-1-yn-1-yl)trimethylsilane (S4)

^{1}H NMR (400 MHz, $CDCl_3$)

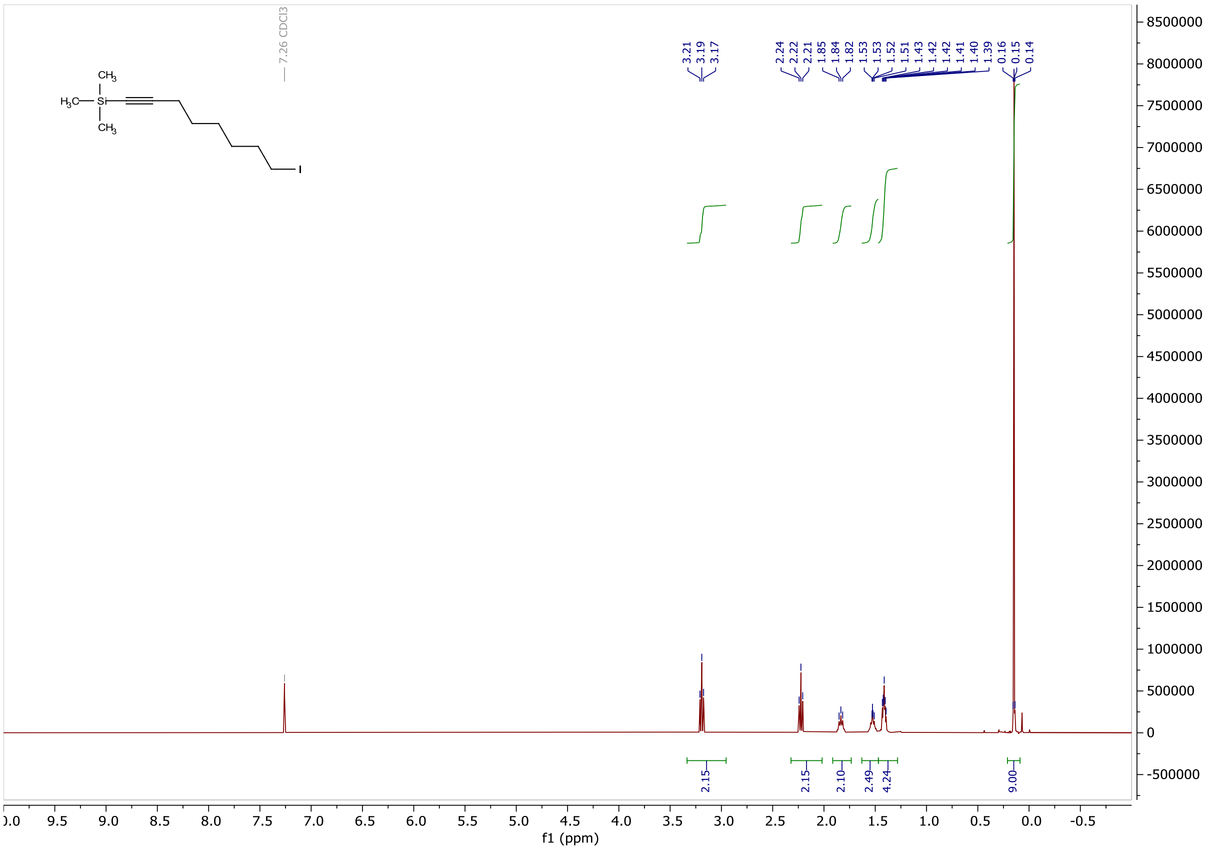

^{13}C NMR (101 MHz, $CDCl_3$)

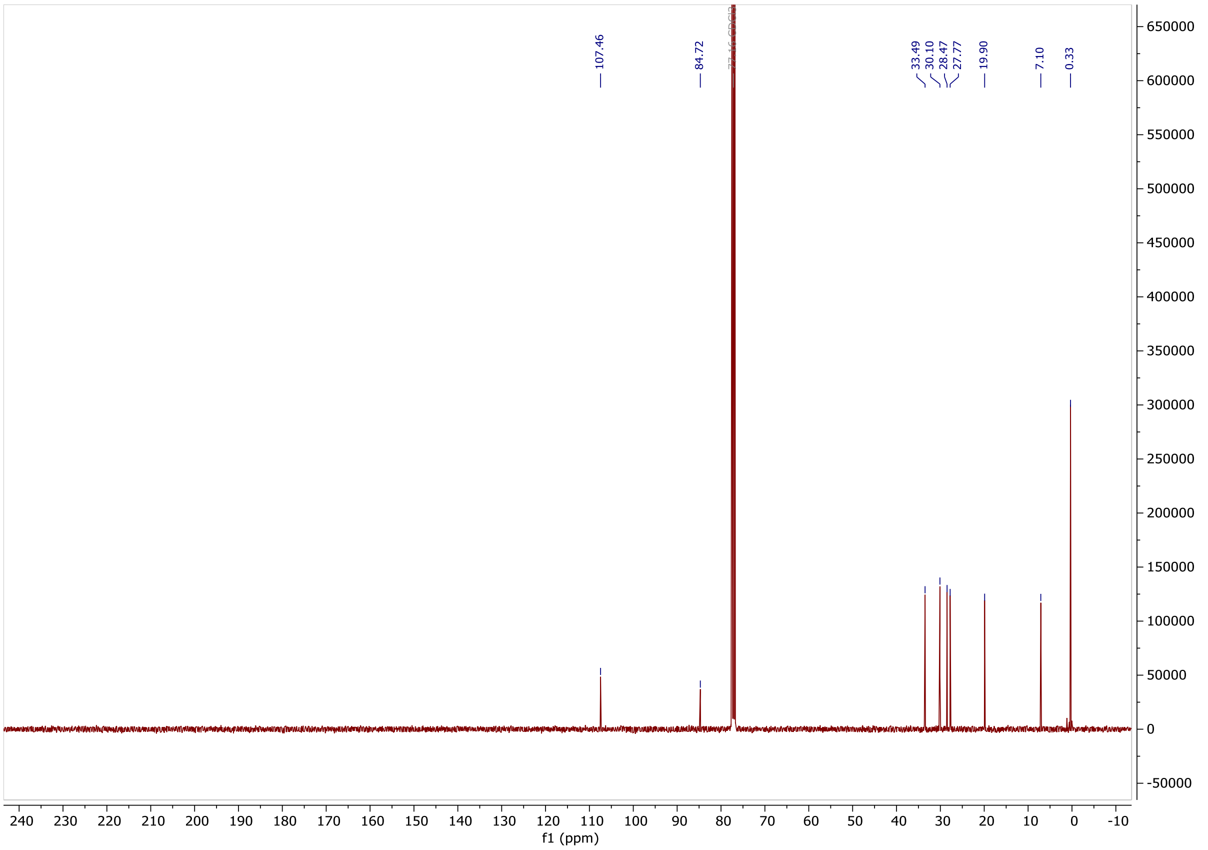

(8-azidooct-1-yn-1-yl)trimethylsilane (S5)

^1H NMR (400 MHz, $CDCl_3$)

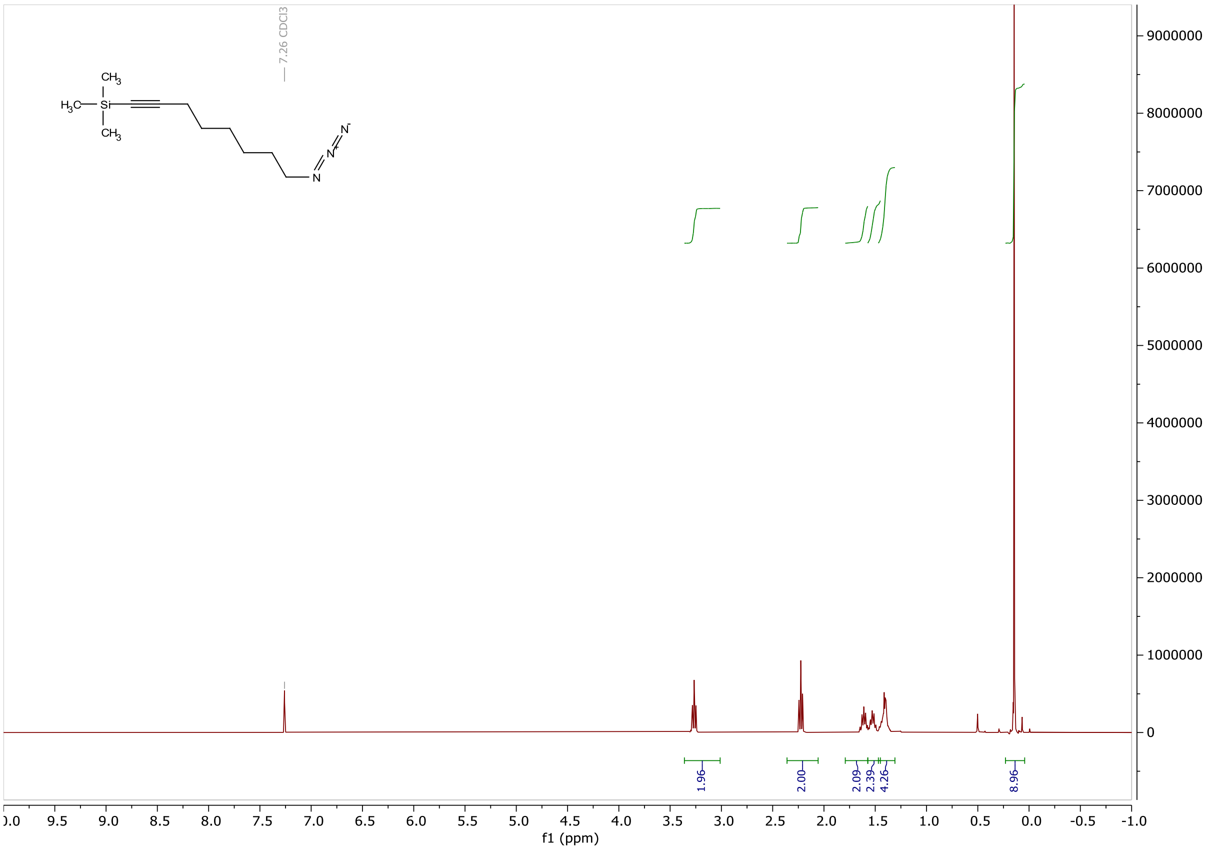

^{13}C NMR (101 MHz, $CDCl_3$)

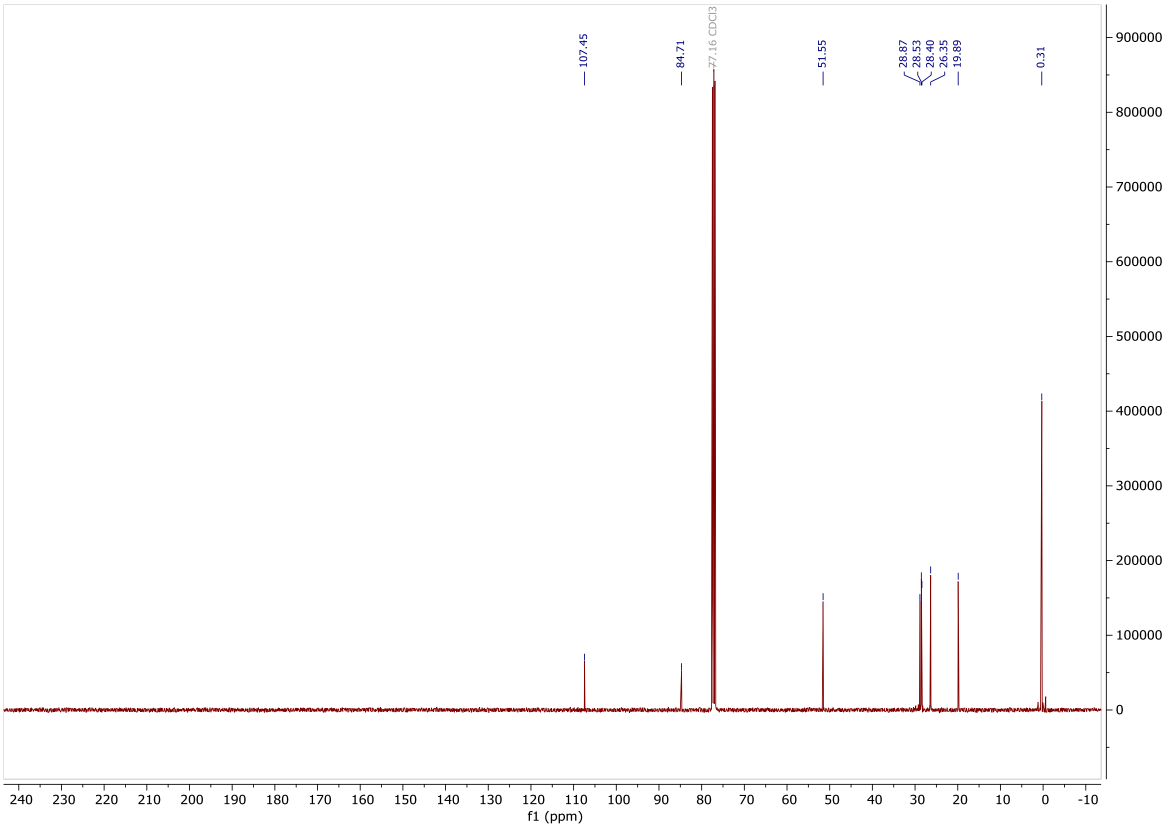

2-iodoterephthalic acid (6)

^{1}H NMR (400 MHz, DMSO)

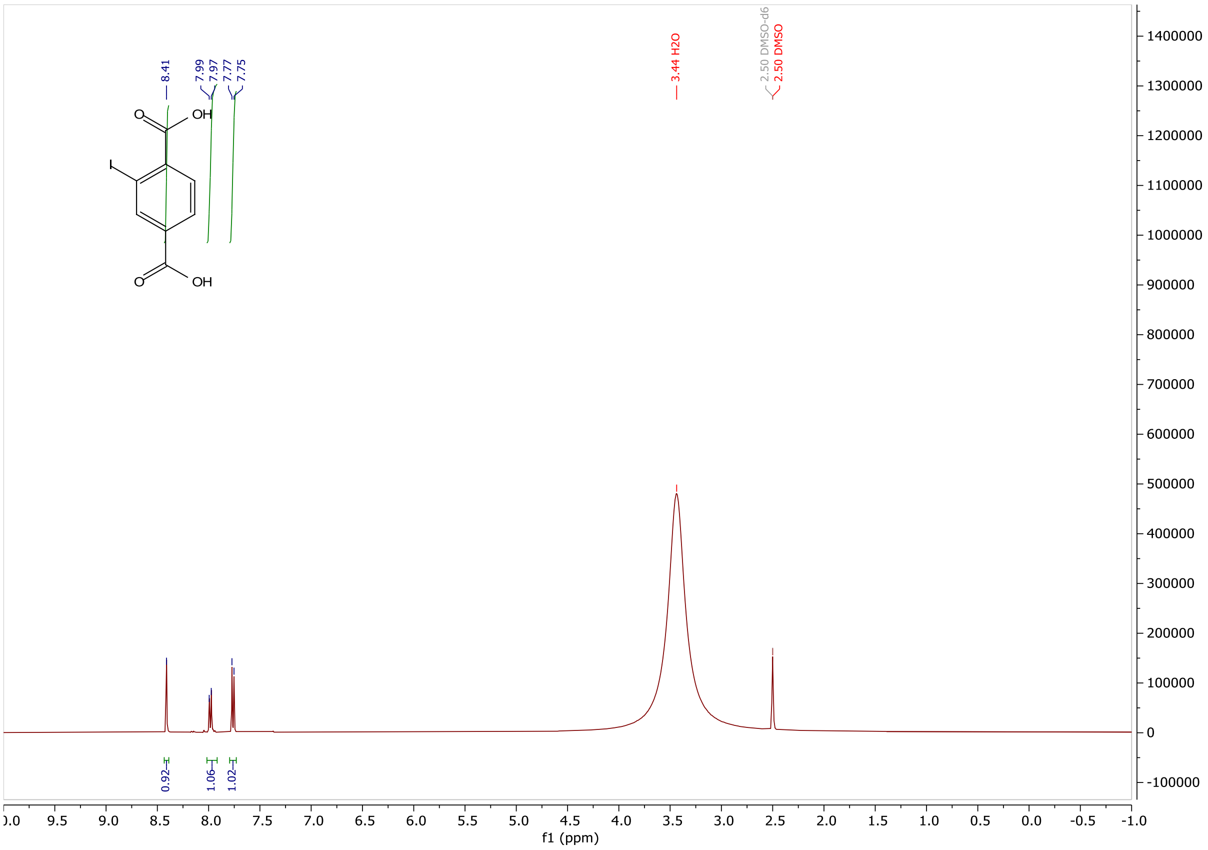

4-oxo-2-iodosylbenzoic acid (16)

^{1}H NMR (400 MHz, DMSO)

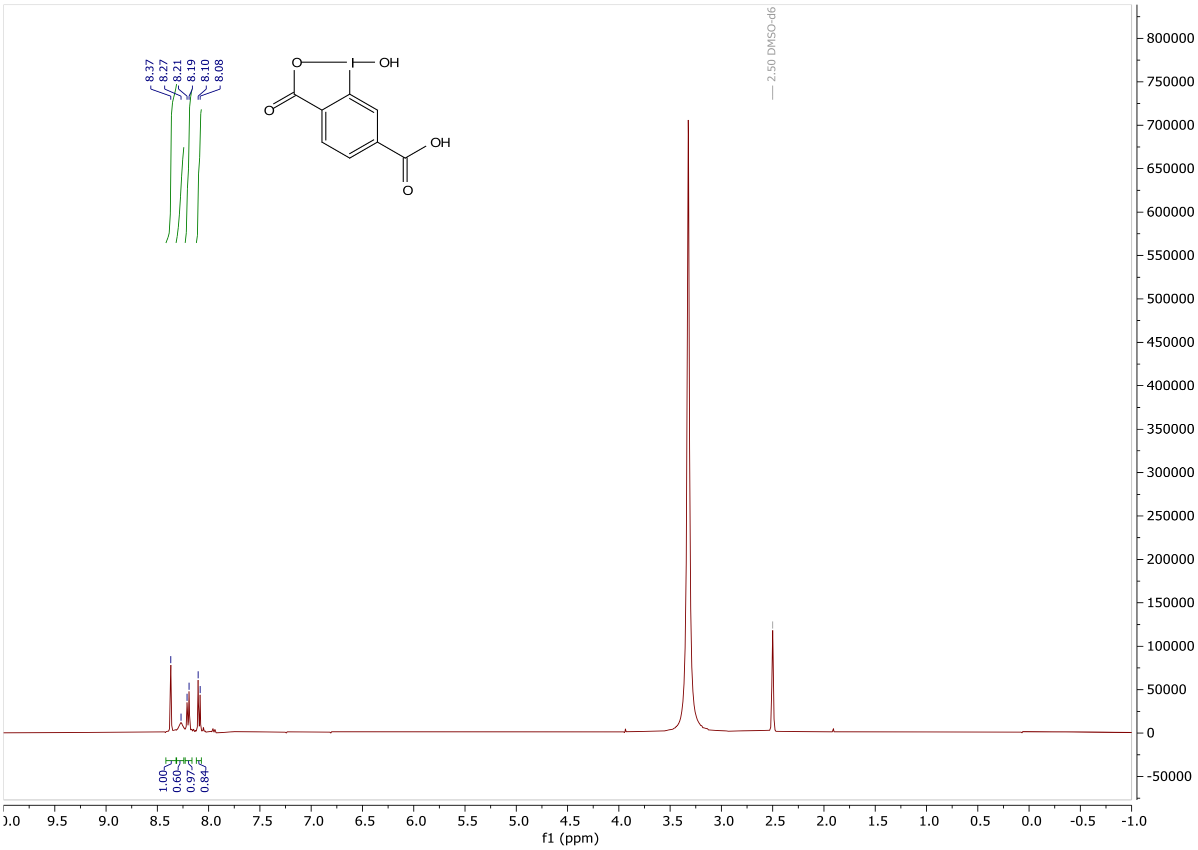

3-Oxo-1-((tri*iso*propylsilyl)ethynyl)-1,3-dihydro-1λ^3-benzo[*d*][1,2]iodaoxole-6-carboxylic (7a)

^{1}H NMR (400 MHz, MeOD)

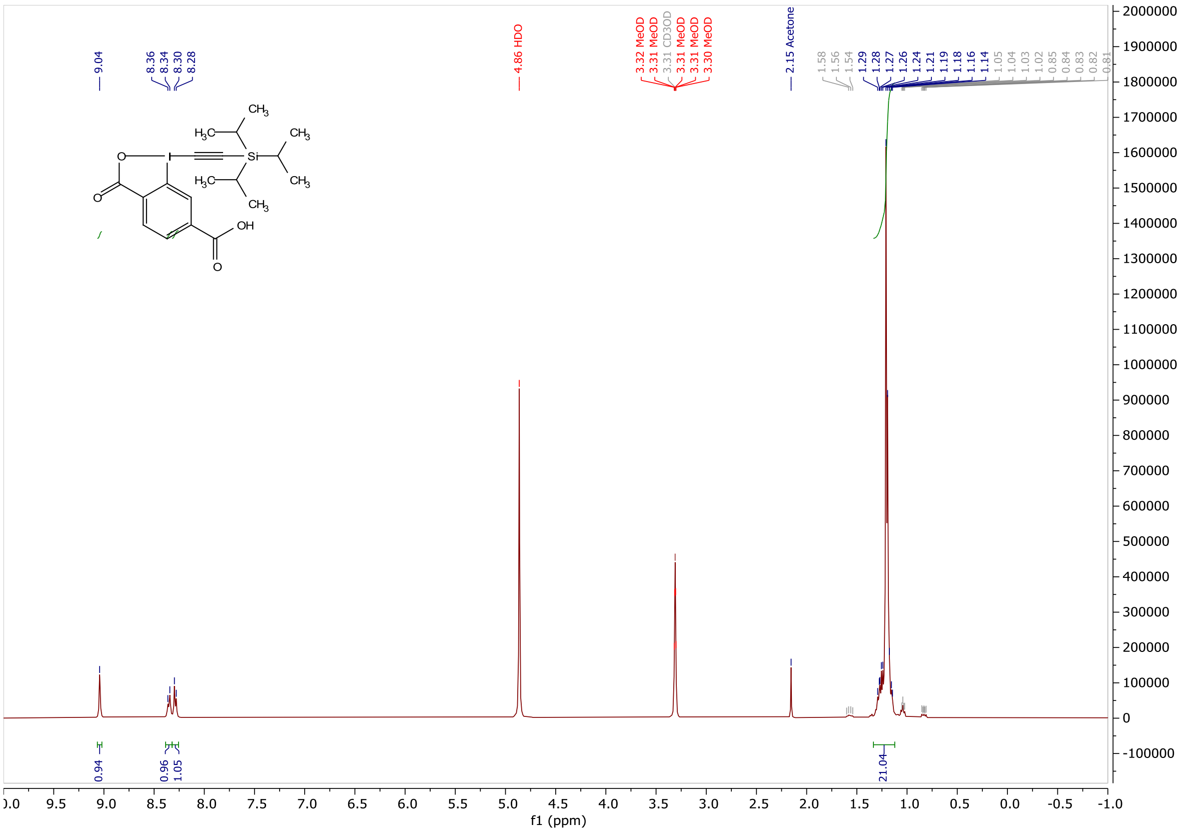

^{13}C NMR (101 MHz, DMSO)

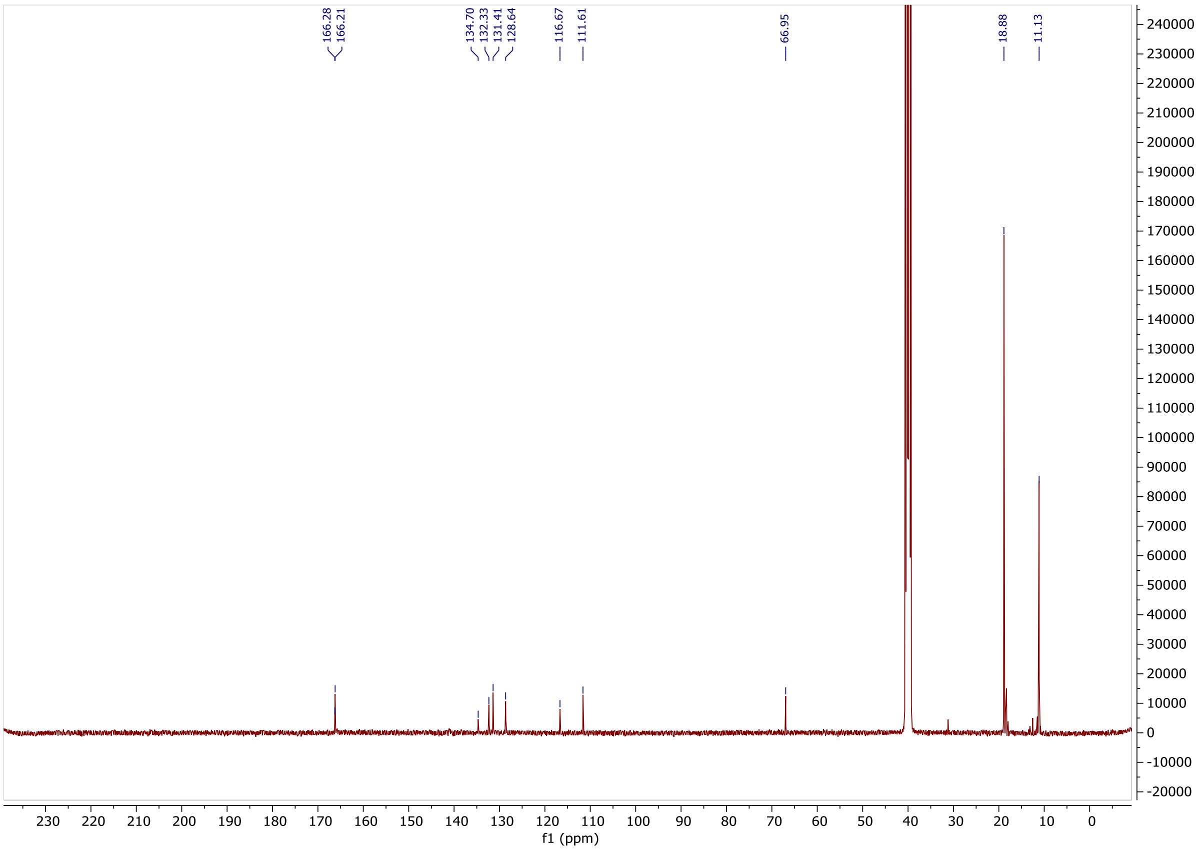

3-oxo-1-((trimethylsilyl)ethynyl)-1,3-dihydro-1λ^3-benzo[*d*][1,2]iodaoxole-6-carboxylic (7b)

^{1}H NMR (400 MHz, DMSO)

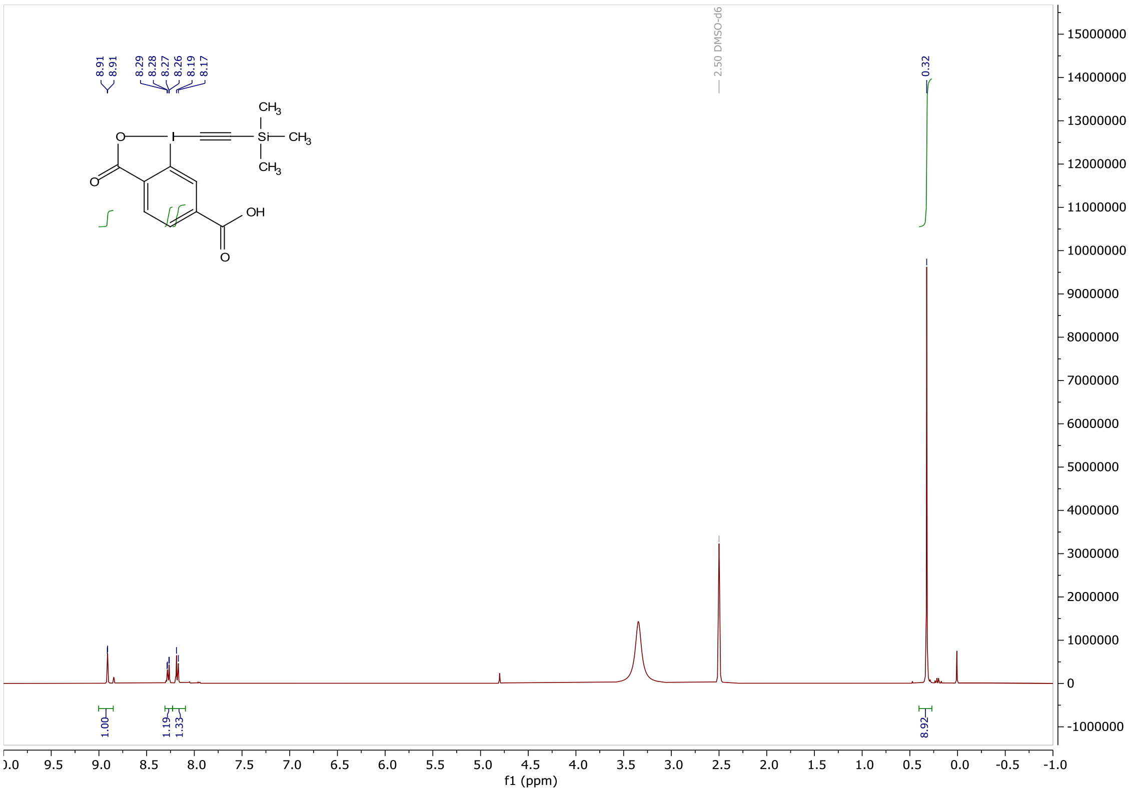

^{13}C NMR (101 MHz, DMSO)

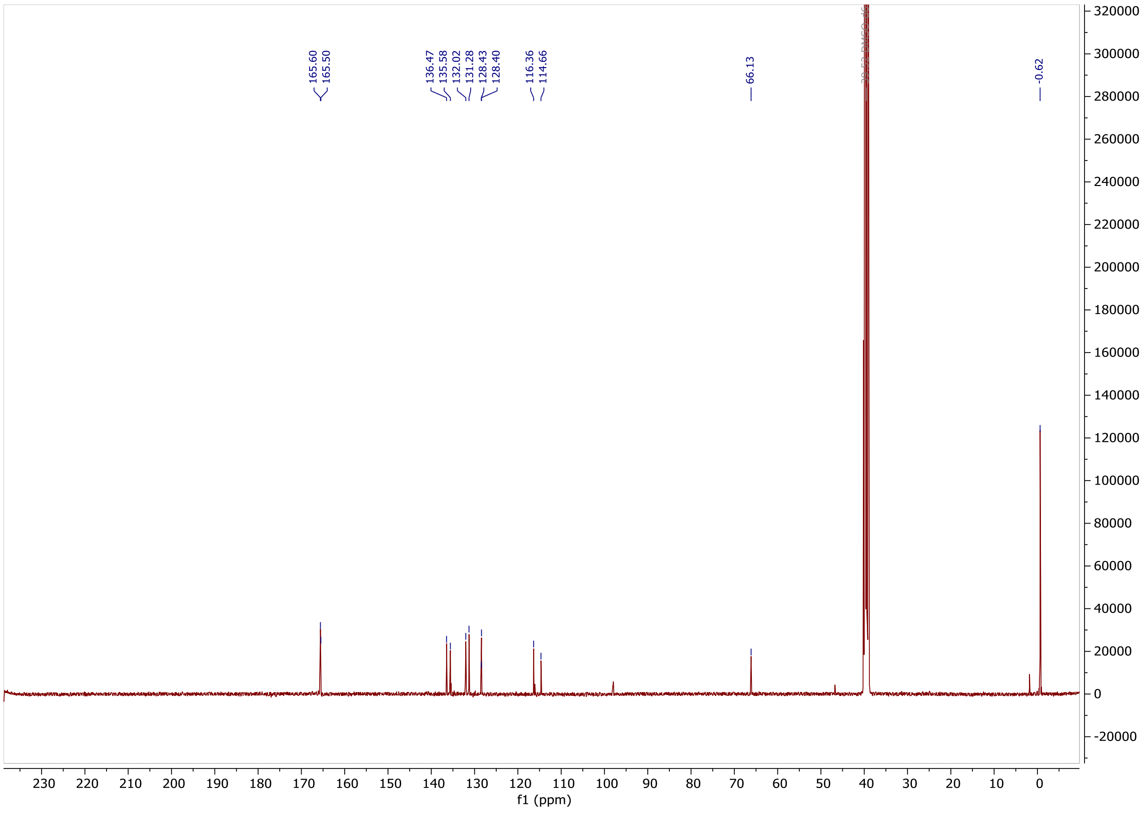

1-(((3-(4-(2-azidoethyl)phenethoxy)propyl)diisopropylsilyl)ethynyl)-3-oxo-1,3-dihydro-1λ³-benzo[*d*][1,2]iodaoxole-6-carboxylic acid (7c)

^{1}H NMR (400 MHz, DMSO)

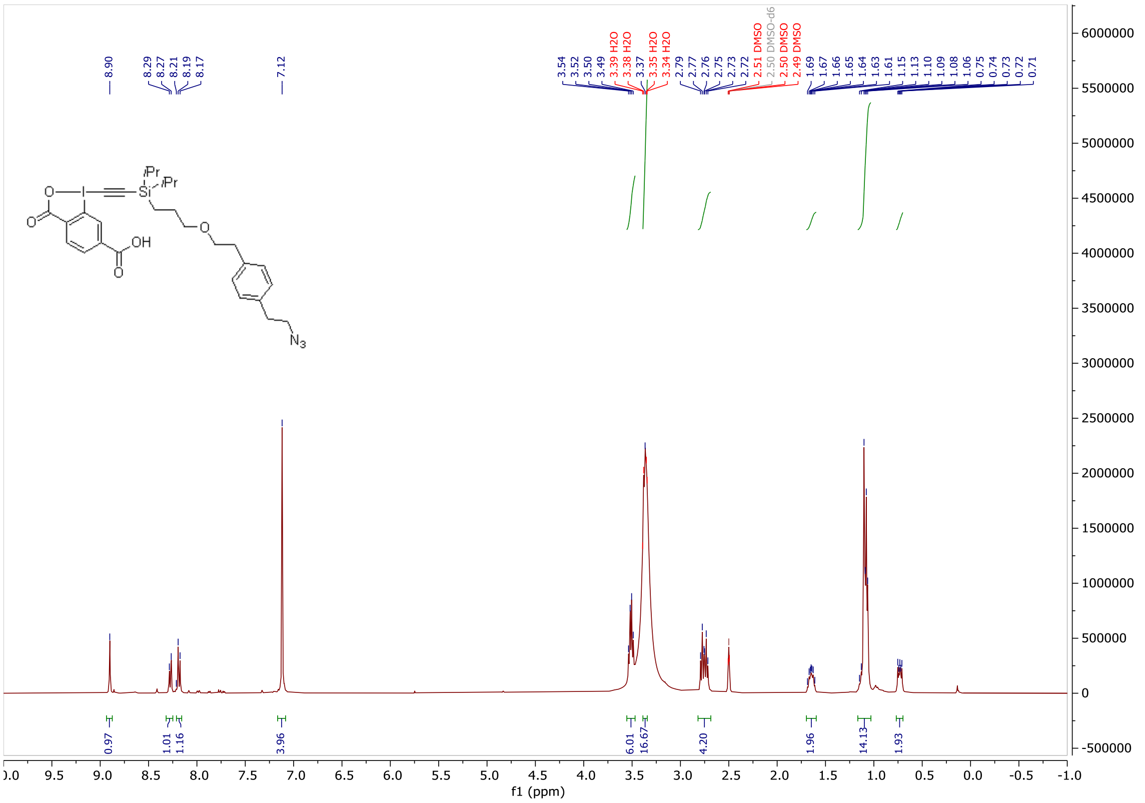

^{13}C NMR (101 MHz, DMSO)

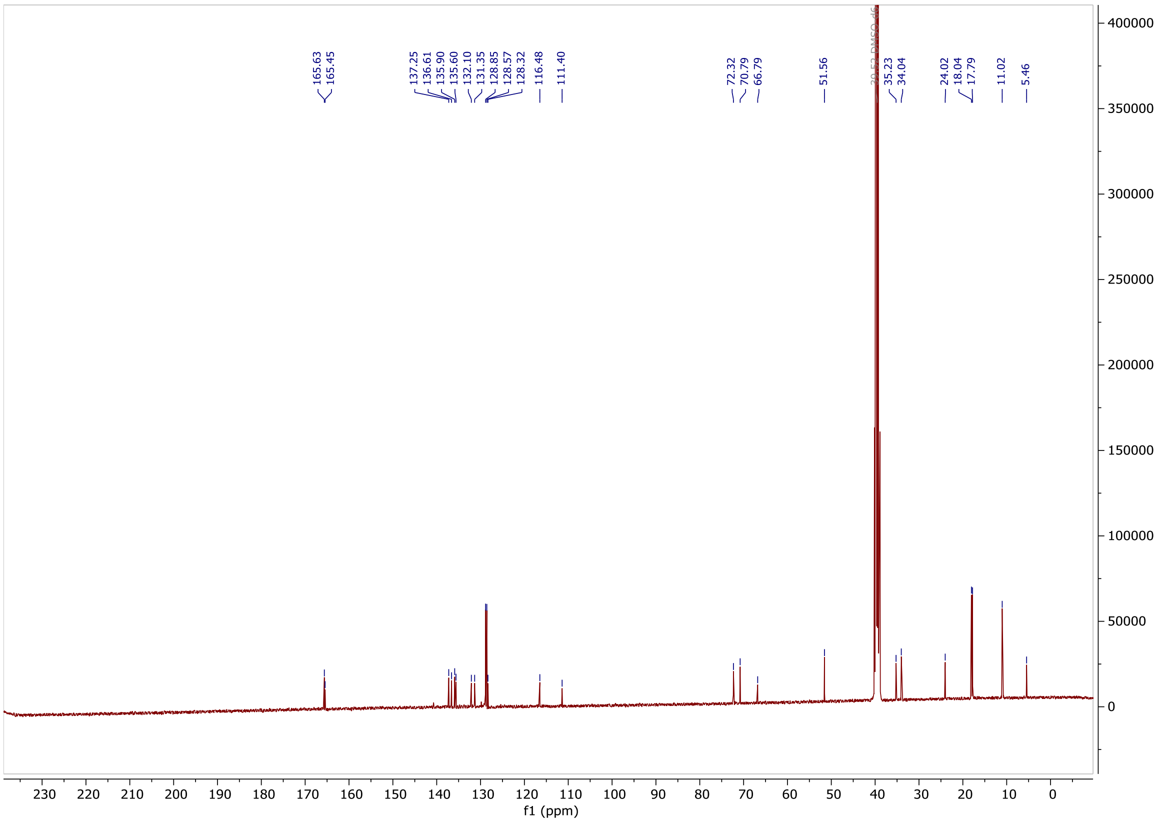

3-oxo-1-((phenylethynyl)-1,3-dihydro-1λ^3-benzo[*d*][1,2]iodaoxole-6-carboxylic (7d)

^{1}H NMR (400 MHz, DMSO)

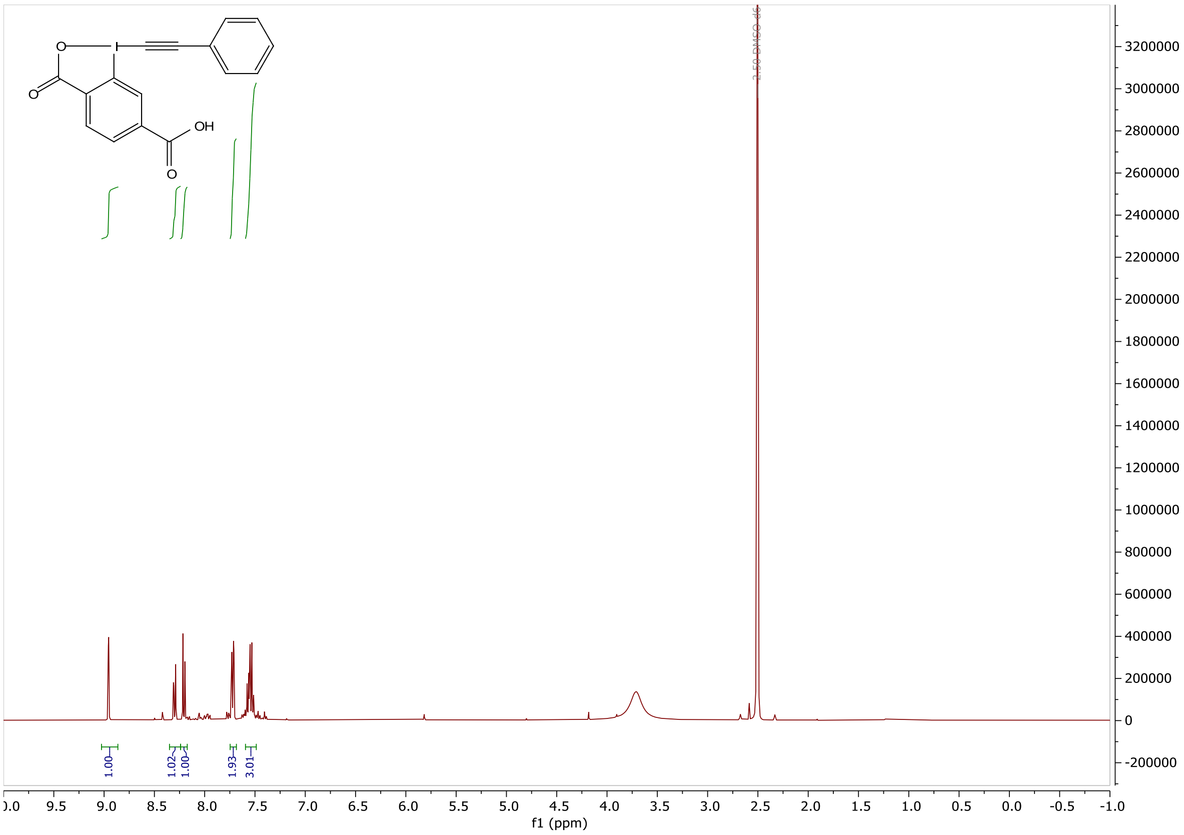

^{13}C NMR (101 MHz, DMSO)

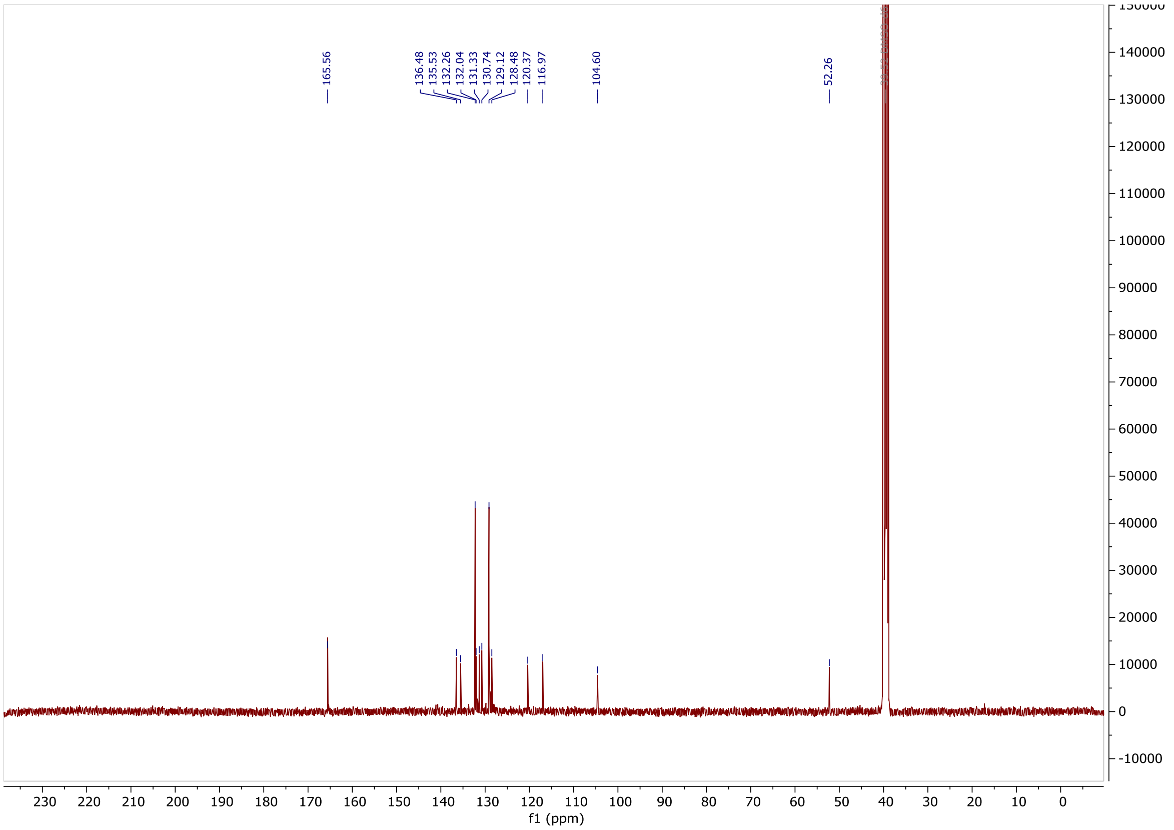

1-(8-chlorooct-1-yn-1-yl)-3-oxo-1,3-dihydro-1λ^3-benzo[*d*][1,2]iodaoxole-6-carboxylic acid (7e)

^{1}H NMR (400 MHz, DMSO)

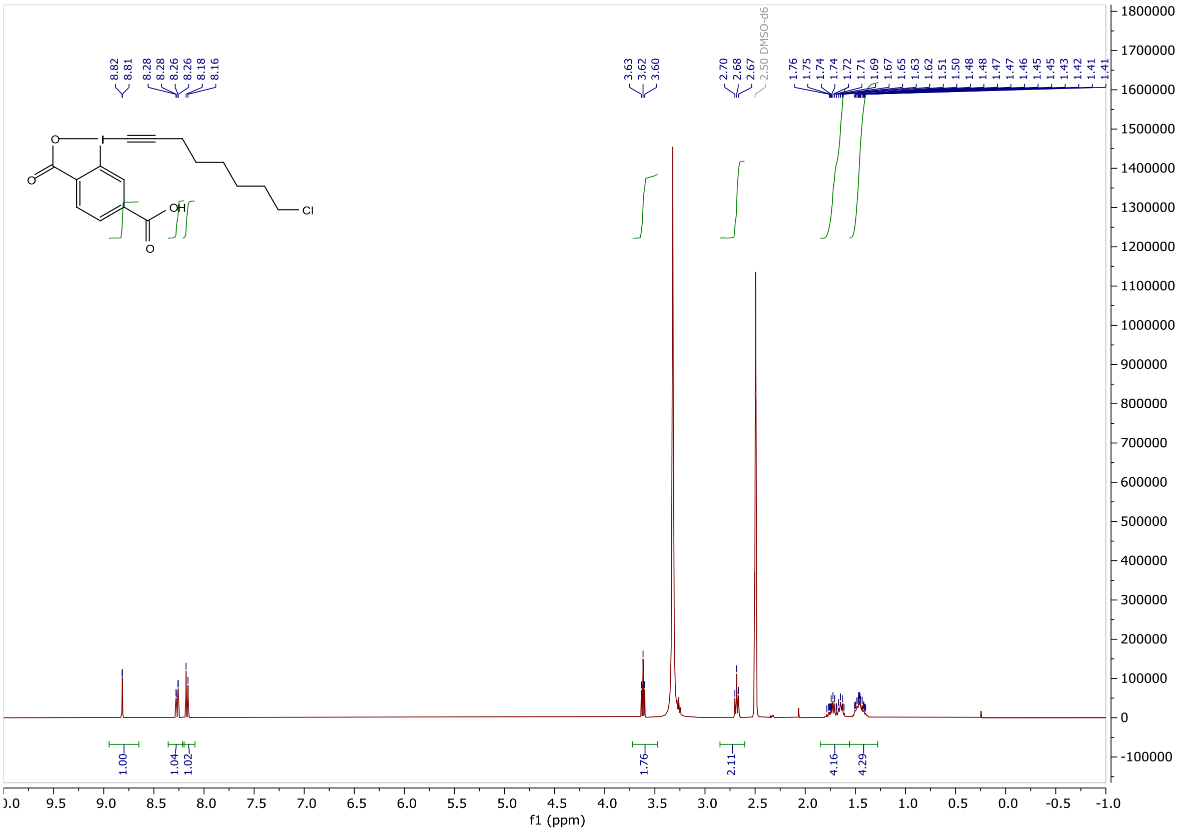

^{13}C NMR (101 MHz, DMSO)

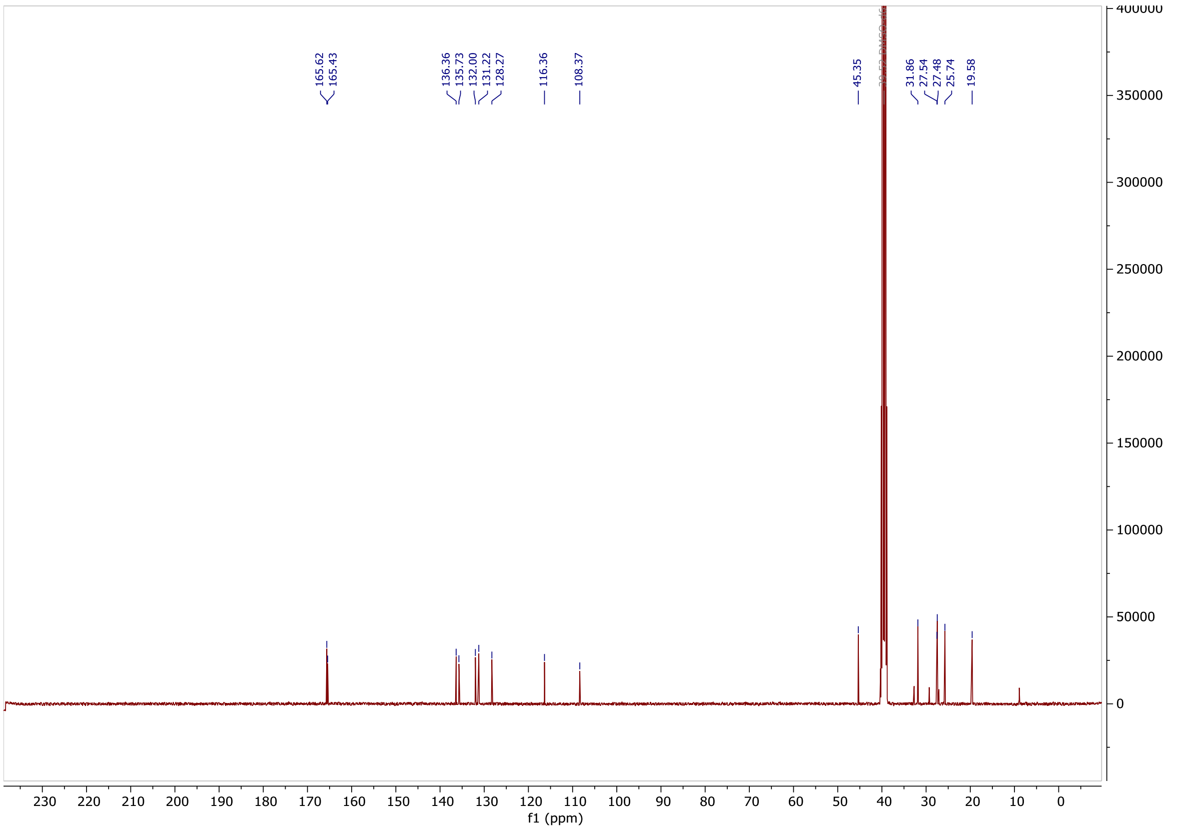

1-(8-azidooct-1-yn-1-yl)-3-oxo-1,3-dihydro-1λ³-benzo[*d*][1,2]iodaoxole-6-carboxylic acid (7f)

^{1}H NMR (400 MHz, DMSO)

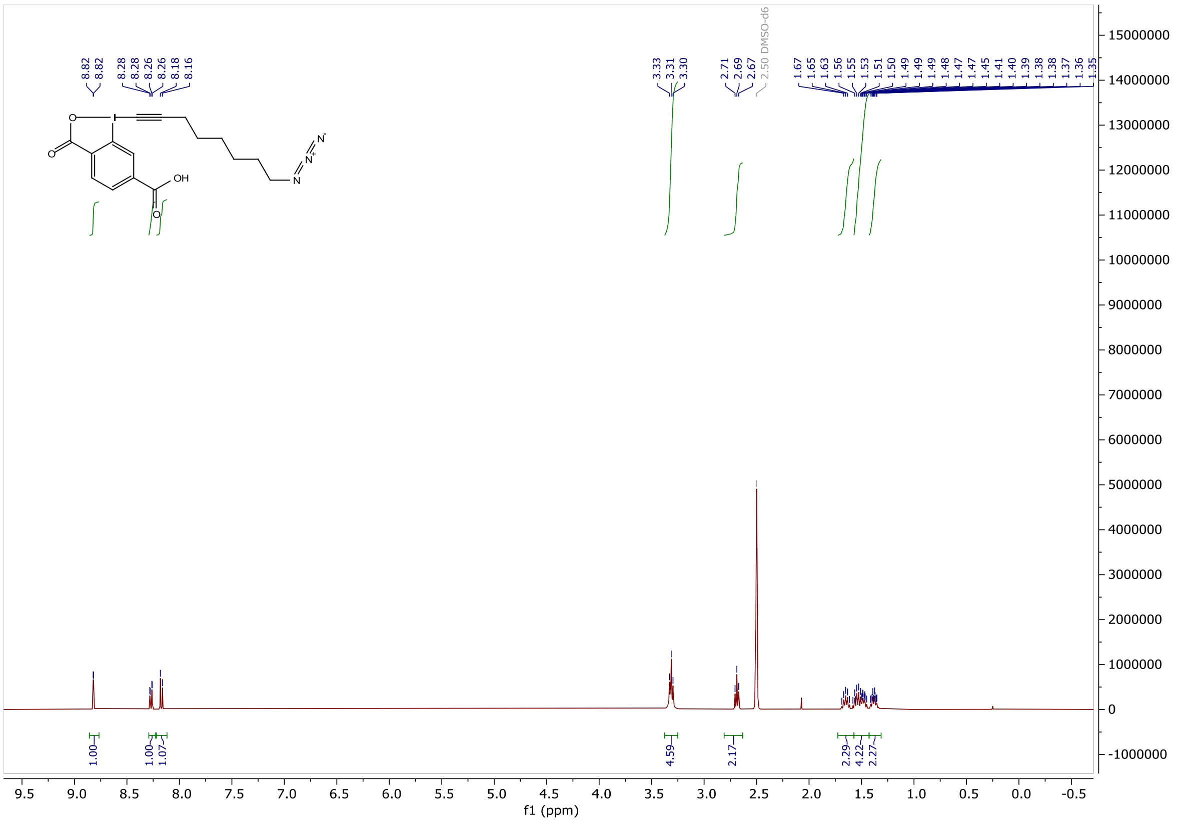

^{13}C NMR (101 MHz, DMSO)

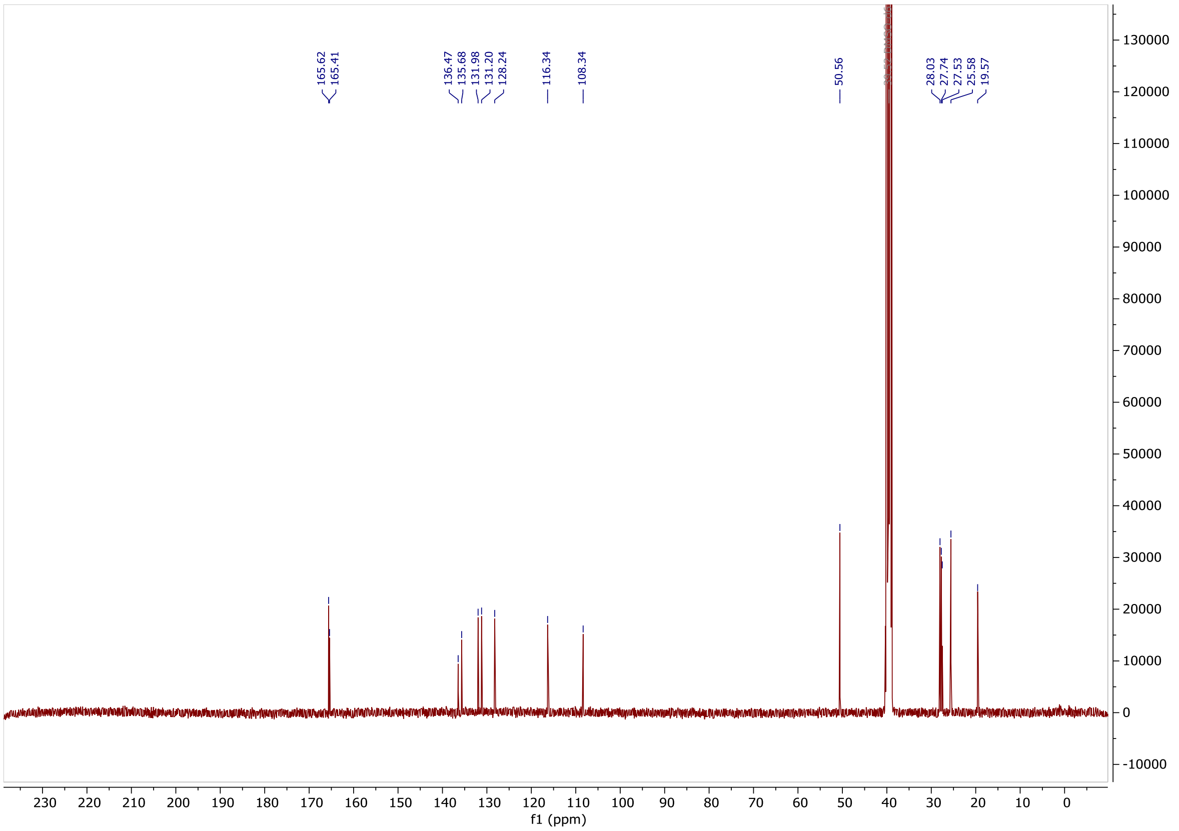

Supplement: Supplementary file 1 [file ja5c13391_si_001.pdf]
